# A Manganese-Chlorella Hydrogel for an Integrated “Remove–Remodel–Repair” Strategy in Pancreatic Cancer Therapy

**DOI:** 10.1007/s40820-026-02344-z

**Published:** 2026-09-09

**Authors:** Tinglin Zhang, Zhu Peng, Shijin Zhao, Xin Jin, Yiqi Du, Zhaoshen Li, Kaixuan Wang, Jie Gao

**Affiliations:** 1 Changhai Clinical Research Unit, Shanghai Changhai Hospital, Naval Medical University, Shanghai, 200433 People’s Republic of China; 2 Department of Gastroenterology, Shanghai Changhai Hospital, Naval Medical University, Shanghai, 200433 People’s Republic of China; 3 College of Life Science, Mudanjiang Medical University, Mudanjiang, 157011 People’s Republic of China; 4 Shanghai Key Laboratory of Nautical Medicine and Translation of Drugs and Medical Devices, Shanghai, 200433 People’s Republic of China; 5 National Key Laboratory of Immunity and Inflammation, Naval Medical University, Shanghai, 200433 People’s Republic of China; 6 Department of Hepatic Surgery, Fudan University Shanghai Cancer Center, Shanghai Medical College, Fudan University, Shanghai, 200032 People’s Republic of China

**Keywords:** Pancreatic cancer, Pancreatic fistula, Integrative therapy, Hydrogels, Photodynamic therapy, cGAS-STING pathway, Immunotherapy

## Abstract

Pancreatic cancer faces the challenges of tumor progression and postoperative pancreatic fistula during the perioperative period, which severely affect patient prognosis. In this study, an injectable self-healing multifunctional hydrogel (CMCGel) was developed based on dynamic cross‑linking between manganese ions (Mn^2+^) and carboxymethyl chitosan loaded with chlorella extract (CE). This platform implements an integrated “Remove**–**Remodel**–**Repair” (3R) strategy for comprehensive perioperative management. CMCGel exhibits distinctive pH/laser dual-responsive behavior: In the acidic tumor microenvironment, the accelerated release of CE and Mn^2+^ enhances photodynamic therapy and chemodynamic therapy (CDT). This combination, activated by laser and gated by endogenous pH, achieves remarkable tumor suppression rates of 85.81% in vitro and 96.01% in vivo. As pH normalizes, Mn^2+^ shifts from CDT to activate the cyclic GMP-AMP synthase stimulator of interferon genes (cGAS-STING) pathway, remodeling the immune microenvironment. Crucially, CMCGel’s adhesion strength in artificial pancreatic fluid at 48 h was 4703.80 ± 50.36 Pa, only 8.17% lower than that at 10 min. This enzyme-resistant property enables durable mechanical sealing at the fistula site, reducing amylase leakage by 55.71% and creating favorable conditions for tissue repair. By integrating pH/laser-triggered combination therapy, immunomodulation, and enzyme-resistant barrier functionality into a single platform, CMCGel presents a clinically promising strategy for perioperative management of pancreatic cancer.

**Supplementary Information:**

The online version contains supplementary material available at https://doi.org/10.1007/s40820-026-02344-z.

## Highlights

- An injectable, self-healing hydrogel (CMCGel) is engineered via dynamic cross-linking of manganese ions and carboxymethyl chitosan to load chlorella extract.
- The pH/laser dual-responsive platform with endogenous pH gating and exogenous laser switching enables a programmed “Remove**–**Remodel**–**Repair” (3R) strategy.
- CMCGel achieves remarkable tumor suppression (96.01% in vivo), effectively remodels the antitumor immune microenvironment, and promotes postoperative pancreatic fistula repair through durable sealing and anti-inflammatory effects.

**Supplementary Information:**

The online version contains supplementary material available at https://doi.org/10.1007/s40820-026-02344-z.

## Graphical abstract

**Figure:**
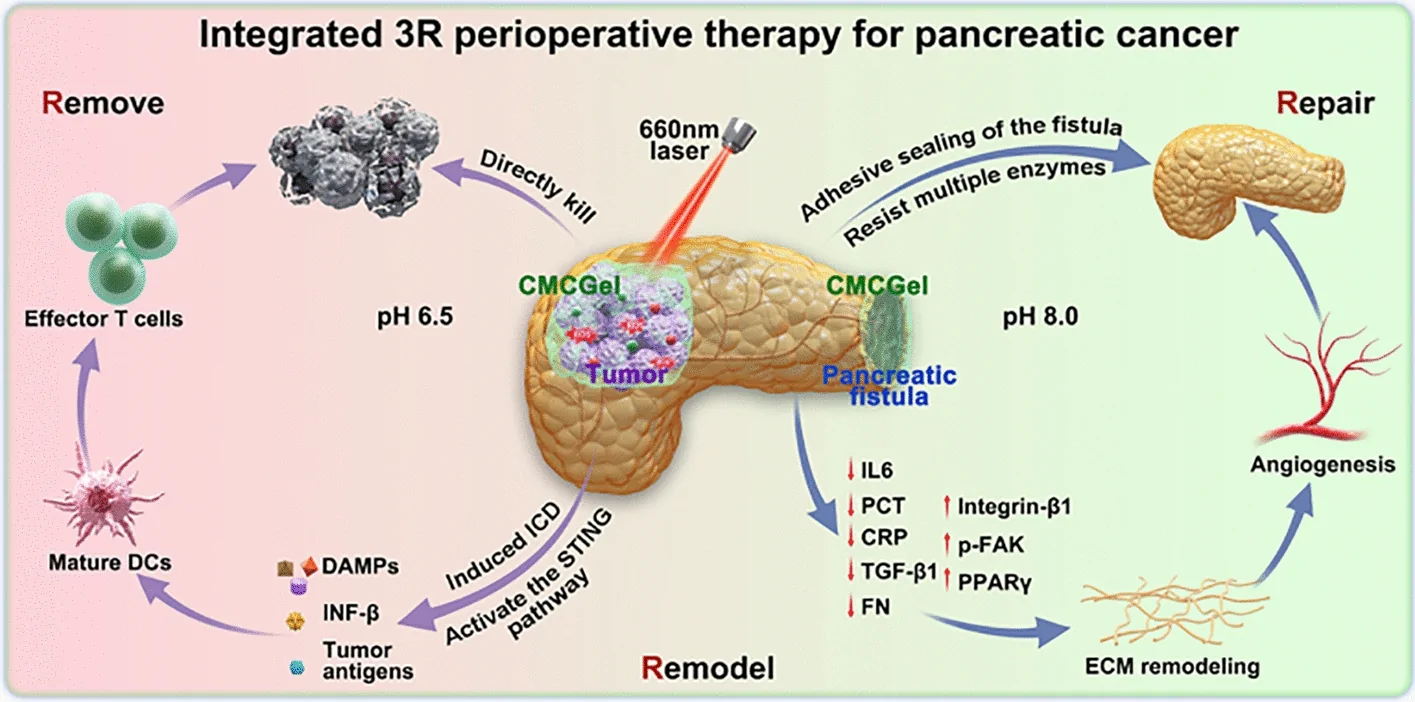

## Introduction

Pancreatic cancer is highly aggressive and lethal [1]. According to the GLOBCAN 2020 data from the International Agency for Research on Cancer (IARC), pancreatic cancer ranks as the seventh leading cause of cancer‑related deaths worldwide and is projected to increase to the second position by 2030 [2]. Currently, surgical resection remains the only potentially curative treatment for pancreatic cancer, yet its efficacy is frequently limited by two major challenges: tumor recurrence and postoperative pancreatic fistula (POPF). The recurrence rate within two years after surgery is approximately 50% [3], while the incidence of POPF ranges from 13% to 64% [4]. Our analysis of six studies [5–10] consistently revealed that POPF significantly shortened patients’ disease-free survival (DFS) and overall survival (OS) (Fig. S1 and Table S1). Moreover, owing to frequent occult vascular invasion or positive resection margins in pancreatic cancer, 7%–50% of patients who are initially deemed “resectable” ultimately undergo only R1 (microscopically incomplete resection) or R2 (macroscopically incomplete resection) resection [11]. These patients not only face a significantly higher risk of recurrence but are also more susceptible to POPF [12]. Therefore, there is an urgent need for an integrated treatment strategy to effectively control perioperative tumor progression while reducing the incidence of POPF.

Currently, effective clinical strategies that simultaneously address both perioperative tumor progression and POPF are lacking [13, 14]. Chemoradiotherapy fails to improve the immunosuppressive microenvironment and is associated with significant systemic toxicity [13]. Fibrin sealant serves merely as a physical barrier and cannot target residual tumors [14]. Existing biomaterials also have limitations: antitumor materials struggle to achieve stable fistula closure in complex enzymatic environments, whereas synthetic gels lack bioactivity and microenvironment-responsive capabilities [13, 14]. Therefore, there is an urgent need to develop biomaterials that integrate stable sealing with spatiotemporally controlled therapeutic functions, enabling the concurrent achievement of tumor clearance, immune modulation, and POPF repair during the perioperative period. This aligns with the holistic medical perspective advocated by the Chinese Anti-Cancer Association (CACA) and represents a critical direction in current translational medicine [15].

Photodynamic therapy (PDT) is an emerging anti‑tumor technique that exerts its therapeutic effects by generating reactive oxygen species (ROS) through the photo‑catalyzed activation of oxygen (O_2_) [16]. Recent phase I/II clinical trials (NCT01770132 and NCT03033225) have confirmed that endosonography-guided PDT is safe and effective for controlling advanced pancreatic cancer [17–19]. This therapy is characterized by precise spatiotemporal controllability, enabling targeted tumor ablation, and ROS-induced cell death is less likely to elicit drug resistance [16–19]. Furthermore, PDT can induce immunogenic cell death (ICD), which subsequently activates antitumor immune responses [16]. Importantly, owing to the significantly greater sensitivity of tumor cells to ROS than normal tissue cells are, PDT can eradicate tumors while maximally preserving surrounding normal tissues [16]. In summary, PDT holds promise as a novel integrated therapeutic approach with the potential for both pancreatic cancer treatment and pancreatic tissue preservation.

Chlorella is a unicellular green alga rich in chlorophyll with PDT effects and polyphenolic compounds that possess anti-inflammatory activity [20]. We have established a systematic chlorella-based biomedical material system [20–22]. We developed a molybdenum nanozyme-chlorella phototherapeutic hydrogel (MC@PHDA), achieving integrated therapy for hepatocellular carcinoma and liver injury [20]. To facilitate translational application, we developed a novel process for preparing chlorella extract (CE) [23]. This process simplifies the traditional preparation workflow for chlorella-based materials, reduces costs, and creates conditions for scaled-up production [23–25]. CE not only has excellent storage stability but also fully retains the integrated therapeutic activity of Chlorella [23–25]. The curdlan-based CE phototherapeutic hydrogel (PCCUR) enables the integrated treatment of melanoma and its postoperative wounds [23]. These studies demonstrate that CE not only has potential as a next-generation PDT photosensitizer but also provides key technological support for the development of novel strategies that combine pancreatic cancer therapy with POPF repair.

Although PDT has demonstrated potential in comprehensive cancer therapy, its ability to independently induce antitumor immune responses remains insufficient [26]. The ability of PDT to activate the cyclic GMP–AMP synthase–stimulator of interferon genes (cGAS–STING) pathway via the release of cytosolic double‑stranded DNA (dsDNA) is limited [26]. Metal ions such as iron (Fe^3+^), magnesium (Mg^2+^), zinc (Zn^2+^), copper (Cu^2+^), and manganese (Mn^2+^) can significantly increase the activity of this pathway through mechanisms that involve improving cGAS catalytic efficiency and promoting STING activation [27–29]. Furthermore, in the acidic tumor microenvironment (TME), Fe^2+^, Cu^2+^, Mn^2+^, and other ions can catalyze the generation of hydroxyl radicals (·OH) from hydrogen peroxide (H_2_O_2_) via chemodynamic therapy (CDT), thereby exerting antitumor effects [30, 31]. On this basis, we propose a strategy that utilizes metal ions to potentiate PDT: combining PDT with CDT to release immunogenic signals such as dsDNA while leveraging metal ions to increase the sensitivity of the cGAS–STING pathway to these signals. This combined strategy is expected to elicit a robust systemic antitumor immune response that is difficult to achieve with monotherapies.

Hydrogels are polymer materials with three-dimensional network structures that are widely applied in the fields of oncology and postoperative wound treatment [32, 33]. Water-soluble polyanionic polysaccharides such as alginate (ALG), carboxymethyl cellulose (CMC), and carboxymethyl chitosan (CMCS) are typically used as scaffolds to form hydrogels via cross-linking between carboxyl groups and metal ions [34–36]. Therefore, in this study, five metal ions (Fe^3+^, Mg^2+^, Zn^2+^, Cu^2+^, and Mn^2+^) capable of activating the cGAS-STING pathway were selected for cross-linking with three polysaccharides (ALG, CMC, and CMCS) to construct hydrogel systems. Adhesiveness and resistance to trypsin were used as the primary evaluation criteria for screening, ultimately determining the hydrogel prepared from CMCS and Mn^2+^ for loading CE. This hydrogel exhibits injectability, adhesiveness, and trypsin resistance. Its unique COO–Mn–OOC bridging structure endows it with dynamic self-healing capability and pH-responsive release properties [37]. Notably, the hydrogel can capture and store tumor antigens via the amino groups of CMCS, thereby enhancing dendritic cell (DC)- and T-cell-mediated antitumor immune responses [38]. These properties contribute to achieving long-term targeted therapy for pancreatic cancer during the perioperative period and promoting effective closure and repair of POPF.

**Scheme 1:**
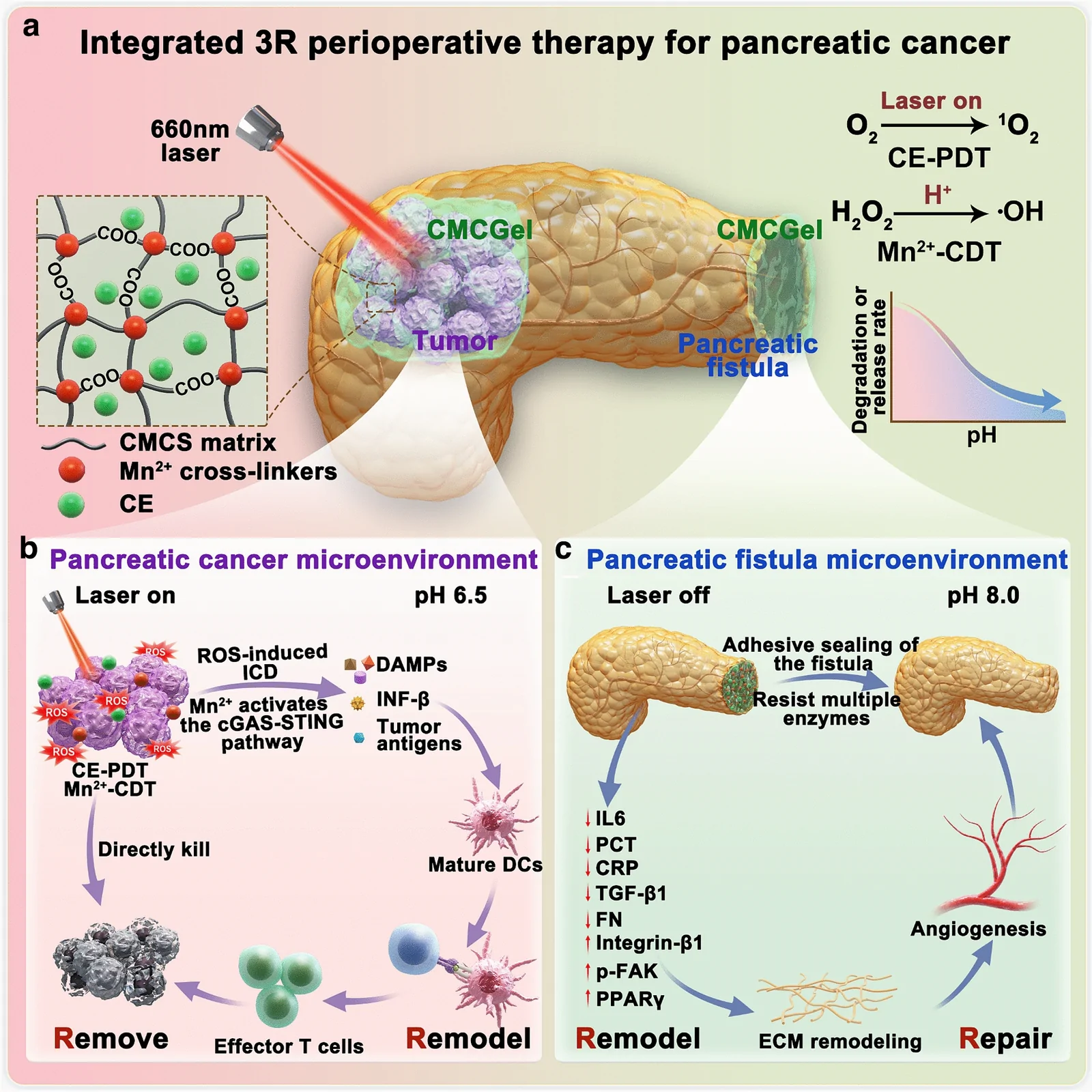
CMCGel hydrogel drives the “3R” strategy for integrated therapy of pancreatic cancer and POPF. **a** Pancreatic cancer patients face the challenges of tumor progression and pancreatic fistula during the perioperative period. **b** Fabrication of CMCGel via Mn^2+^ cross-linking with CMCS and loading of CE. **c** Therapeutic mechanism: In the removal phase, CE-mediated PDT and Mn^2+^-mediated CDT collaboratively generate ROS to eradicate tumor cells. In the remodeling phase, PDT/CDT-induced ICD combined with Mn^2+^ activates the cGAS-STING pathway, promoting DC maturation and T-cell infiltration to initiate systemic antitumor immunity. In the repair phase, CMCGel provides stable adhesion in the enzymatic environment to seal the fistula, whereas CE accelerates healing through its anti-inflammatory properties

To address the clinical challenges associated with perioperative pancreatic cancer progression and POPF, we developed a novel hydrogel system (CMCGel). This system utilizes CMCS as the backbone and Mn^2+^ as the cross-linking agent and encapsulates the photosensitizer CE within its three-dimensional network. Through spatiotemporally controllable PDT combined with its micro-environmental pH-responsive properties, CMCGel represents a programmed therapeutic strategy for perioperative pancreatic cancer control and POPF repair (Scheme 1). Specifically, CMCGel employs two independent yet synergistic regulatory mechanisms: (i) endogenous pH gating, which automatically controls the macroscopic rhythm of CDT, degradation, and release, and (ii) exogenous laser switching, which precisely initiates and terminates PDT on demand. In the acidic tumor microenvironment (pH ~ 6.5) with laser “ON”, CE-mediated PDT and Mn^2+^-mediated CDT generate a synergistic ROS burst, killing tumor cells and inducing ICD—which embodies the “Remove” principle. As the tumor is eliminated and the pH normalizes (to ~ 7.4), Mn^2+^ shifts from CDT to activate the cGAS-STING pathway, while CMCS captures released tumor antigens, collectively promoting DC maturation and T-cell infiltration to remodel the antitumor immune microenvironment—the “Remodel” principle. Upon laser “OFF” and transition to an alkaline POPF environment (pH ~ 8.0), ROS generation ceases; CMCGel provides durable physical sealing because of its adhesiveness and enzyme resistance, whereas CE exerts anti-inflammatory effects to promote fistula repair—the “Repair” principle. A three-tier defense mechanism (maximal primary tumor elimination, sustained systemic immune memory, and physical barrier isolation) mitigates the potential risk of residual tumor progression during the prorepair phase. Through this “pH-dictated rhythm, laser-determined precision” dual-regulation design, CMCGel innovatively established a “3R” therapeutic paradigm that simultaneously addresses multiple perioperative challenges, including tumor control and POPF repair, through a single system. It not only offers a potential management strategy for pancreatic cancer patients but also has significant value for improving therapeutic efficacy and alleviating clinical burden.

## Experimental Section

See the Supporting Information for the experimental details.

## Results and Discussion

### Analysis of Postoperative Complications in Pancreatic Cancer

POPF is a prevalent and serious complication following pancreatic surgery. In 2005, the International Study Group of Pancreatic Fistula (ISGPF) initially defined POPF as the presence of amylase in the drainage fluid on postoperative day three that exceeded three times the upper limit of normal serum levels; POPF was categorized into three grades (A, B, and C) according to clinical severity [39]. To elucidate the impact of POPF on the prognosis of patients with pancreatic cancer, we conducted a systematic review of the relevant literature across the MEDLINE, Embase, Scopus, Web of Science, and Cochrane Library databases. DFS was defined as the interval from tumor resection to recurrence, whereas OS was defined as the period from surgery to death or the last follow-up. The inclusion criteria for study were as follows: (1) patients with pancreatic cancer who underwent surgical treatment; (2) POPF was diagnosed according to the standards set by the ISGPF in either 2005 or 2016; (3) studies that reported the effect of POPF on OS and/or DFS; and (4) studies designed as either prospective or retrospective cohort studies.

Initially, 35 articles were identified, from which 6 studies were ultimately selected for analysis concerning the impact of POPF on survival outcomes [5–10]. The six analyses indicated that POPF significantly reduced DFS (studies 1–6: *p* = 0.002, 0.02, < 0.01, < 0.001, 0.028, 0.031) and OS (studies 1–6: *p* = 0.020, 0.005, < 0.01, 0.002, < 0.001, 0.027). The detailed data are presented in Fig. S1, and the fundamental characteristics of the included studies are outlined in Table S1. These findings consistently suggest that POPF serves as an independent risk factor for postoperative survival in patients with pancreatic cancer, potentially because of mechanisms involving the systemic inflammatory response induced by pancreatic juice leakage, subsequent infection, and the creation of an immunosuppressive microenvironment. At present, the clinical management of postoperative complications in patients with pancreatic cancer is notably limited. Specifically, strategies for managing POPF primarily emphasize mechanical closure and do not possess antitumor properties [14]. Concurrently, traditional antitumor therapies prove ineffective in addressing POPF [16]. Consequently, the development of an integrated therapeutic platform capable of concurrently addressing fistula closure and pancreatic cancer is urgently needed.

### Preparation and Characterization of the CMCGel Hydrogels

As illustrated in Fig. S2a, polyethylene glycol (PEG) was employed as the solvent to extract the active compounds from the broken chlorella powder, resulting in the production of a green CE. With increasing concentration, the color intensity of the CE solution progressively intensified (Fig. S2b). The UV‒Vis spectrum revealed prominent absorption peaks at 412.0 and 667.0 nm (Fig. S2c), which align with the characteristic peaks of chlorophyll [40]. The chlorophyll a content in the CE was quantitatively determined through UV‒visible spectrophotometry, which revealed that chlorophyll a constituted 7.0% of the dry weight of the CE. Notably, over a 28-day observation period, the CE exhibited no significant color fading (Fig. S2d), and its characteristic absorption peaks remained stable at temperatures of 4, 25, and 37 °C (Fig. S2e). These findings demonstrate the high stability of CE and its chlorophyll components, ensuring its efficacy in the sustained treatment of PDT [23].

**Fig. 1:**
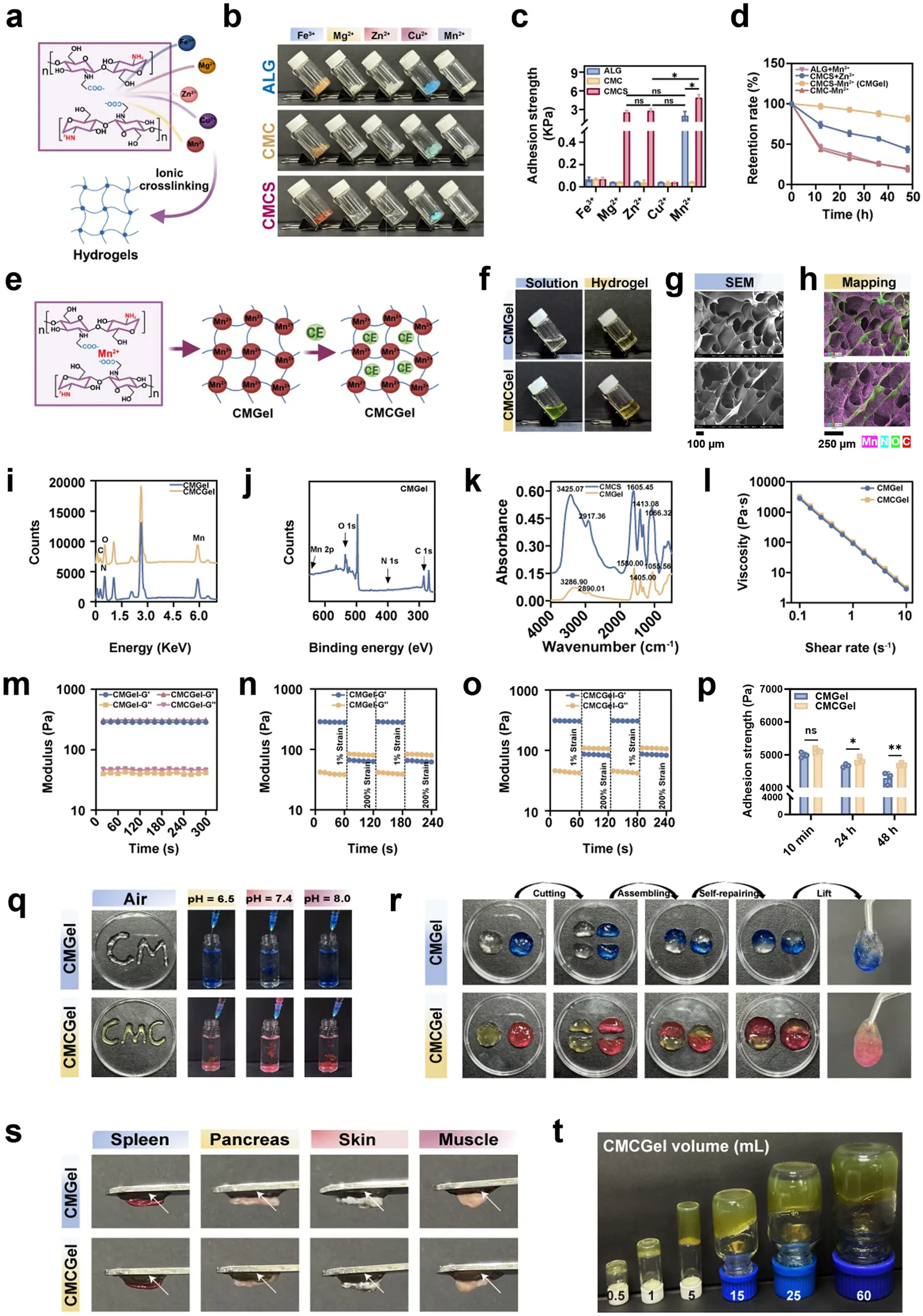
Preparation and characterization of the CMCGel hydrogels. **a** Schematic diagram of the cross-linking mechanism of metal ion‒polysaccharide hydrogels. **b** Photographs of 15 types of metal ion‒polysaccharide hydrogels. **c** Adhesion strength in a trypsin environment. **d** Mass retention rate in a trypsin environment. **e** Schematic diagram of the cross-linking mechanism. **f** Photographs, **g** SEM images (scale bars = 100 μm), **h** elemental mapping images (scale bars = 250 μm), and **i** EDS spectra of CMGel and CMCGel hydrogels. **j** XPS spectra and **k** FTIR spectra of CMGel. The rheological characterization of the two hydrogels includes **l** injectability, **m** elastic modulus and viscous modulus, and **n–o** self-healing properties. **p** Wet-state tissue adhesion strength of the two hydrogels in an APF microenvironment. The two hydrogels** q** injectability photographs in air and PBS, **r** self-healing photographs in air, and **s** adhesion photographs in air (white arrows point to the hydrogels). **t** Scalable preparation photographs of CMCGel from 0.5 to 60 mL. The data are expressed as the means ± SDs (n = 3). *p < 0.05, **p < 0.01, ***p < 0.001, ****p < 0.0001

We undertook the design and systematic screening of 15 distinct metal ion‒polysaccharide cross-linked hydrogels. We utilized five multivalent metal cations (Fe^3+^, Mg^2+^, Zn^2+^, Cu^2+^, and Mn^2+^), which are known for their ability to activate the cGAS-STING signaling pathway [27–29], and cross-linked them with three well-established polyanionic polysaccharides, ALG, CMC, and CMCS [34–36], to construct hydrogel matrices (Fig. 1a, b). By concentrating on adhesion strength and the mass retention rate within a pancreatic enzyme environment as key performance metrics, we determined the optimal formulation that demonstrated both robust adhesion and resistance to pancreatic enzymes. Our results revealed that the adhesion strength (4.89 ± 0.40 kPa) (Fig. 1c) and mass retention rate (81.98% ± 2.93%) (Fig. 1d) were greatest in CMGel synthesized through the cross-linking of CMCS with Mn^2+^ after 48 h of incubation with pancreatic enzymes. This formulation ranked first among the 15 groups and was subsequently selected for further experimentation. Notably, Mn^2+^ confers an “acid-responsive” smart switch to CMGels: within the acidic TME, it catalyzes the conversion of endogenous H_2_O_2_ into toxic ·OH via a Fenton-like reaction, thereby enhancing the antitumor efficacy of CDT for PDT [31]. Conversely, in neutral to alkaline normal tissues, this reaction remains largely inactive, avoiding off-target damage and achieving tumor-selective therapy [31].

As depicted in Fig. 1e, the CE was subsequently combined with the CMCS and subsequently cross-linked with Mn^2+^ to synthesize the CE-loaded CMCGel hydrogel. The gelation process was visually demonstrated through a tilting bottle experiment (Fig. 1f), where the addition of Mn^2+^ induced a transition of both the CMCS solution and the CMCS-CE mixture from a fluid state to a stable solid form, confirming the successful formation of both CMGel and CMCGel. The formulation underwent additional refinement, emphasizing “gel uniformity” (Fig. S3a, b) and “biocompatibility with human umbilical vein endothelial cells (HUVECs)” (Fig. S3c–e). Through process optimization, the preparation protocol for CMCGel was established as follows: A homogeneous mixture was obtained by combining CMCS solution (600 μL, 75.0 mg mL^−1^), Mn^2+^ solution (500 μL, 50.0 mM), and CE solution (200 μL, 487.5 μg mL^−1^), followed by incubation for cross-linking for 30 min to form the hydrogel. Notably, all the reagents were sterilized by ultraviolet irradiation after weighing, and solution preparation and mixing operations were performed within a laminar flow hood. The final concentrations of each component in CMCGel were determined to be 34.6 mg mL^−1^ CMCS, 26.9 mM Mn^2+^, and 75.0 μg mL^−1^ CE Scanning electron microscopy (SEM, Fig. 1g) revealed that both hydrogels exhibited interconnected three-dimensional porous network structures, which are advantageous for the absorption of excess tissue exudate and the controlled release of CE and Mn^2+^ [32, 33]. Quantitative analysis of the porous network structure, as observed in the SEM images (Fig. S4a–c), revealed that compared with CMGel (259 μm, 76.33%), CMCGel significantly reduced the average pore size and porosity (~ 144 μm, 46.00%). This reduction is attributed to the increased solid content of the hydrogel resulting from CE loading, which effectively decreased the pore size and porosity, thereby densifying the framework structure. Moreover, after a storage period of 28 days at 4, 25, and 37 °C, the dry weight of CMCGel remained nearly constant (Fig. S5), indicating excellent stability of the solid content.

We subsequently investigated the cross-linking mechanism of the hydrogel. Elemental mapping (Figs. 1h and S6a) and energy-dispersive spectroscopy (EDS, Figs. 1i and S6b) revealed that both hydrogels contained carbon (C), nitrogen (N), oxygen (O), and Mn. In CMGel, the percentage of C is 47.41% that of N is 2.62%, that of O is 40.25%, and that of Mn is 9.72%, whereas in CMCGel, the percentage of C is 48.19%, that of N is 2.04%, that of O is 40.70%, and that of Mn is 9.07%. X-ray photoelectron spectroscopy (XPS, Figs. 1j and S6c) further confirmed the presence of these elements, with characteristic peaks observed at approximately 642 and 653 eV, corresponding to the spin-orbital splitting peaks of Mn 2*p*_3/2_ and 2*p*_1/2_. This indicates that Mn is present in the + 2 oxidation state, which is essential for cross-linking with the carboxyl groups in CMCSs. Fourier transform infrared spectroscopy (FTIR, Fig. 1k) revealed that the asymmetric stretching vibration peak (1605.45 cm^−1^) and the symmetric stretching vibration peak (1413.08 cm^−1^) of the carboxyl groups in CMCS redshifted to 1580.00 and 1405.00 cm^−1^, respectively, in CMGel, with a reduction in peak intensity. This suggests that Mn^2+^ forms COO-Mn-OOC bridging structures with carboxyl groups through electrostatic and metal coordination interactions. Furthermore, the stretching vibration peak of the hydroxyl groups in CMCS, originally observed at 3425.07 cm^−1^, redshifted to approximately 3286.90 cm^−1^ and decreased in intensity in CMGel. This observation suggests that the hydroxyl groups may have been involved in the cross-linking process through the formation of a limited number of hydrogen bonds. Importantly, the C–O–C stretching vibration peak slightly redshifted from 1066.32 to 1055.56 cm^−1^, with negligible changes in peak width. These findings indicate that preserving the β-(1 → 4)-glycosidic bond structure within the chitosan backbone is essential for maintaining the stability of the hydrogel framework. Zeta potential analysis (Fig. S6d) revealed that compared with CMCS, CMGel exhibited a positive charge. This alteration is attributed to the presence of free amino groups in the CMCS following cross-linking with Mn^2+^, which reversed the overall charge of the system. The resulting positive charge enhances the capacity of the hydrogel to adsorb antigens released by negatively charged tumor cells and improves its adhesion to tissues [22, 38]. In conclusion, the cross-linking and structural stability of the CMCGel hydrogel are achieved primarily through electrostatic and metal coordination interactions between Mn^2+^ and the carboxyl groups in the CMCS, resulting in the formation of COO‒Mn‒OOC bridging structures. Additionally, a limited number of hydrogen bonds contribute to the cross-linking and structural stability of the hydrogel.

The injectability and moldability of the hydrogel allow it to conform effectively to various irregular wound surfaces, including POPF. Its relatively stable mechanical strength, coupled with excellent self-healing properties, is crucial for ensuring effective wound closure [41]. To comprehensively assess the performance of the two hydrogels, their properties were systematically evaluated. First, we characterized the hydrogels via a rheometer and a mechanical tester. Shear-thinning experiments (Fig. 1l) demonstrated that as the angular frequency increased from 0.1 to 10 s^−1^, the viscosity of both hydrogels decreased by two orders of magnitude, indicating their favorable shear-thinning properties and, consequently, excellent injectability. The storage modulus (G’) and loss modulus (G”) test results (Fig. 1m) revealed that during the 300-s test period, the G’ values for both hydrogels were notably greater than the G’ values, with CMGel and CMCGel having G’ values of approximately 283 and 306 Pa, respectively. These findings suggest that both hydrogels retained their state and demonstrated strong mechanical properties because of the stable cross-linking network formed between the CMCS and Mn^2+^. We further assessed the G’ values of the hydrogels following incubation in PBS (pH 6.5, 7.4, and 8.0) and artificial pancreatic fluid (APF, pH 8.0) for 10 min. As shown in Fig. S7a, the G’ values of both hydrogels varied significantly across different pH microenvironments (*p* < 0.01) and increased with increasing pH. Importantly, at pH 8.0, no significant difference in G’ was observed between the two hydrogels when they were incubated in PBS or APF, indicating their resistance to enzymatic degradation. This is attributed to the insensitivity of both the β-(1 → 4)-glycosidic bonds of CMCS and the COO–Mn–OOC cross-linking structure of the hydrogels to the enzymes present in APF [22, 38]. Notably, at pH 8.0, the increase in G’ of CMCGel relative to that of CMGel was markedly greater than that under other pH conditions (*p* < 0.01). These findings indicate that CE enhances the modulus of CMCGel in a weakly alkaline microenvironment-dependent manner. These findings indicate that CE reinforces the modulus of CMCGel in a manner dependent on weakly alkaline microenvironmental triggering. This enhancement arises from the oxidation of polyphenolic moieties in CE to quinone structures under weakly alkaline conditions, followed by Schiff base cross-linking with the amino groups of CMCS, thereby increasing the cross-linking density of CMCGel [23–25]. In the alternating strain scanning test (Fig. 1n, o), both the G’ and G” of the hydrogels exhibited good reversible behavior. Under high shear strain (200%), G’ temporarily fell below G,” indicating partial network disruption; however, when the strain was reduced to a low level (1%), G’ quickly recovered, indicating excellent structural recovery. This repeatable “damage–repair” behavior confirms that the self-healing properties of the hydrogel are excellent, mainly because the diffusion medium provided by the hydrogel in the wet environment for Mn^2+^ makes the COO-Mn-OOC cross-linking structure dynamically reversible [35]. Notably, the addition of CE did not interfere with this dynamic cross-linking mechanism.

Hydrogels intended for POPF treatment must achieve stable and effective adhesion to wet tissue in a pancreatic fluid microenvironment, thereby ensuring reliable sealing of the opening of the fistula [41]. In this study, the adhesion strength of the hydrogel under wet tissue conditions mimicking the pancreatic fluid microenvironment was quantitatively evaluated. Specifically, scratches were created on porcine skin surfaces to simulate tissue wounds, and the hydrogel was uniformly applied to the wound area. The samples were then placed in APF and continuously shaken at 100 rpm at a constant temperature of 37 °C to simulate the physiological environment in vivo. Samples were collected at 10 min, 24 h, and 48 h to evaluate the stability and efficacy of the adhesive performance of CMCGel. As shown in Fig. 1p, both hydrogels exhibited tissue adhesion properties. At the 10-min time point, no statistically significant difference in adhesive strength was observed between the two formulations. However, at 24 h, compared with CMGel, CMCGel demonstrated significantly greater adhesion strength (*p* < 0.05), and this difference further increased at 48 h (*p* < 0.01). Notably, although the adhesive strength of both hydrogels slightly decreased over time, CMCGel maintained an adhesive strength of 4703.80 ± 50.36 Pa at 48 h, representing only an 8.17% reduction from the baseline value recorded at 10 min. These results indicate that the hydrogel cross-linked by CMCS and Mn^2+^ exhibits wet tissue adhesion in the pancreatic fluid microenvironment, whereas the incorporation of CE significantly enhances the adhesive capacity of CMCGel. Further mechanistic studies revealed that the wet tissue adhesion of CMCGel arises from the synergistic effects of multiple mechanisms: the amino and hydroxyl groups of CMCS form electrostatic interactions and hydrogen bonds with tissue proteins [22, 38]; the polyphenolic structures in CE are oxidized to quinone structures in the weakly alkaline environment (pH 8.0) of APF, leading to covalent cross-linking with tissue proteins [23–25]; subsequently, the 3D network of the hydrogel undergoes physical entanglement and mechanical interlocking with the tissue [32, 33]. Collectively, these mechanisms endow CMCGel with excellent wet tissue adhesion properties, indicating its potential for sealing POPF.

We subsequently evaluated the stability of the two hydrogels in the APF microenvironment using the mass retention rate as the assessment metric (Fig. S7b). During the first two days, the mass retention rates of CMGel and CMCGel remained above 87% and 91%, respectively, indicating relatively intact hydrogel structures. This is attributed to the lack of direct degradative effects of trypsin, lipase, and amylase in the APF on the β-(1 → 4)-glycosidic bonds of CMCS and the COO–Mn–OOC cross-linking structures of the hydrogel [22, 38], as well as the limited erosion of the gel skeleton by the fluid environment in the initial phase [41]. From day 3 onward, the hydrogels entered a rapid degradation phase. Compared with the CMGel, the CMGel completely degraded on day 7, whereas the CMCGel completely degraded on day 7.5, indicating a delay of approximately 0.5 days. This difference is attributed to the oxidation of polyphenolic structures in CE to quinone structures in the weakly alkaline environment (pH 8.0) of APF, which subsequently undergo Schiff base reactions with the amino groups of CMCS, forming secondary covalent crosslinks that increase the cross-linking density of CMCGel and thereby delay its degradation [23–25]. Notably, during the critical 3-day postoperative window for POPF prevention as defined by international consensus [5–10, 39], CMCGel demonstrated favorable enzyme tolerance and relatively intact structural integrity; accelerated degradation began on the 4th postoperative day, aligning with the temporal sequence of POPF repair. This functional transition from barrier function to tissue repair prevention avoids the clinical risks of premature failure or foreign body retention, facilitating an integrated therapeutic strategy for POPF sealing and repair.

Furthermore, we comprehensively assessed the injectability, tissue adhesion, and scalability of the synthesis of the hydrogels of the hydrogel. As illustrated in Fig. 1q, CMGel, which was stained with Coomassie Brilliant Blue (blue), and CMCGel, which was stained with Rhodamine B (red), were successfully extruded through a syringe and molded into the shapes of “CM” and “CMC,” respectively. This process clearly demonstrates their exceptional injectability and moldability. Notably, when placed in phosphate-buffered saline (PBS) solutions with varying pH values, the extruded hydrogels retained a stable solid state, highlighting their robust structural stability in moist physiological environments with different pH values. We subsequently assessed the self-healing ability of the hydrogels through a series of experiments involving staining, cutting, recombination, healing, and lifting (Fig. 1r). The findings revealed that the CMGel achieved complete healing within just 3 min after being cut and reassembled, and the healed gel was capable of supporting its own weight while being suspended in air. During this investigation, the diffusion of Coomassie Brilliant Blue dye from the unstained regions to the stained regions provided additional evidence of structural reintegration. Similarly, CMCGel demonstrated comparable self-healing properties. The adhesive performance of the two hydrogels on four distinct tissues, namely, the spleen, pancreas, skin, and muscle, is shown in Fig. 1s. The findings indicated that the hydrogels adhered robustly to each tissue surface under gravitational influence, primarily because of robust interactions between the hydrogel and the tissue surface. Moreover, the hydrogel preparation was successfully scaled up, as depicted in Fig. 1t, with an increase in volume from 500 μL to 60 mL. The gelation process maintained its efficiency, and the hydrogel consistently displayed green coloration. This observation indicates that the cross-linking reaction between Mn^2+^ and CMCS has excellent scalability and stability.

In conclusion, this study introduces an innovative “one-step cross-linking and multifunctional integration” approach for the development of injectable CMCGel hydrogels. By utilizing CMCS as the structural backbone and Mn^2+^ as the dynamic cross-linking center, a reversible COO‒Mn‒OOC bridging network is established through electrostatic‒coordination interactions with the in situ incorporation of CE. This method eliminates the need for toxic initiators or high-energy irradiation, offering a simple and safe process that preserves the properties of the hydrogel during scaled-up production. The hydrogel simultaneously possesses injectability, self-healing ability, tissue adhesiveness, and stability in the pancreatic multienzyme environment, enabling effective sealing of various irregular wounds [41]. Furthermore, its exceptional properties present extensive application prospects for local injection treatments of gastrointestinal diseases under endoscopic ultrasound guidance [17–19]. In the context of drug delivery, the three-dimensional porous network structure of CMCGel not only facilitates efficient drug loading and sustained release but also establishes a conducive microenvironment for cell migration and the healing of POPF [41]. In terms of component design, CE effectively retains active chlorophyll components over an extended period, with its porphyrin structure providing robust support for subsequent PDT [23]. Concurrently, Mn^2+^ initiates CDT to synergize with PDT for antitumor effects only in the acidic TME, thereby minimizing off-target damage to normal tissues [31]. This reaction remains inactive in neutral normal tissues, thereby minimizing off-target damage and ensuring tumor-selective multimodal therapy. CMCGel exhibits superior biocompatibility. Compared with traditional single anticancer drugs [16], POPF sealing materials [14], and suturing techniques [42], CMCGel not only demonstrates superior adaptability to complex wound environments but also effectively addresses the stability challenges posed by the pancreatic multienzyme milieu. These findings highlight its distinct advantages in balancing antitumor and POPF closure therapies. As a result, the CMCGel hydrogel, with its innovative preparation strategy and multifunctional integration capabilities, holds significant promise as a next-generation smart biomaterial for applications in tumor treatment and tissue repair.

### Dual pH/Laser-Responsive Properties of CMCGel Hydrogels

**Fig. 2:**
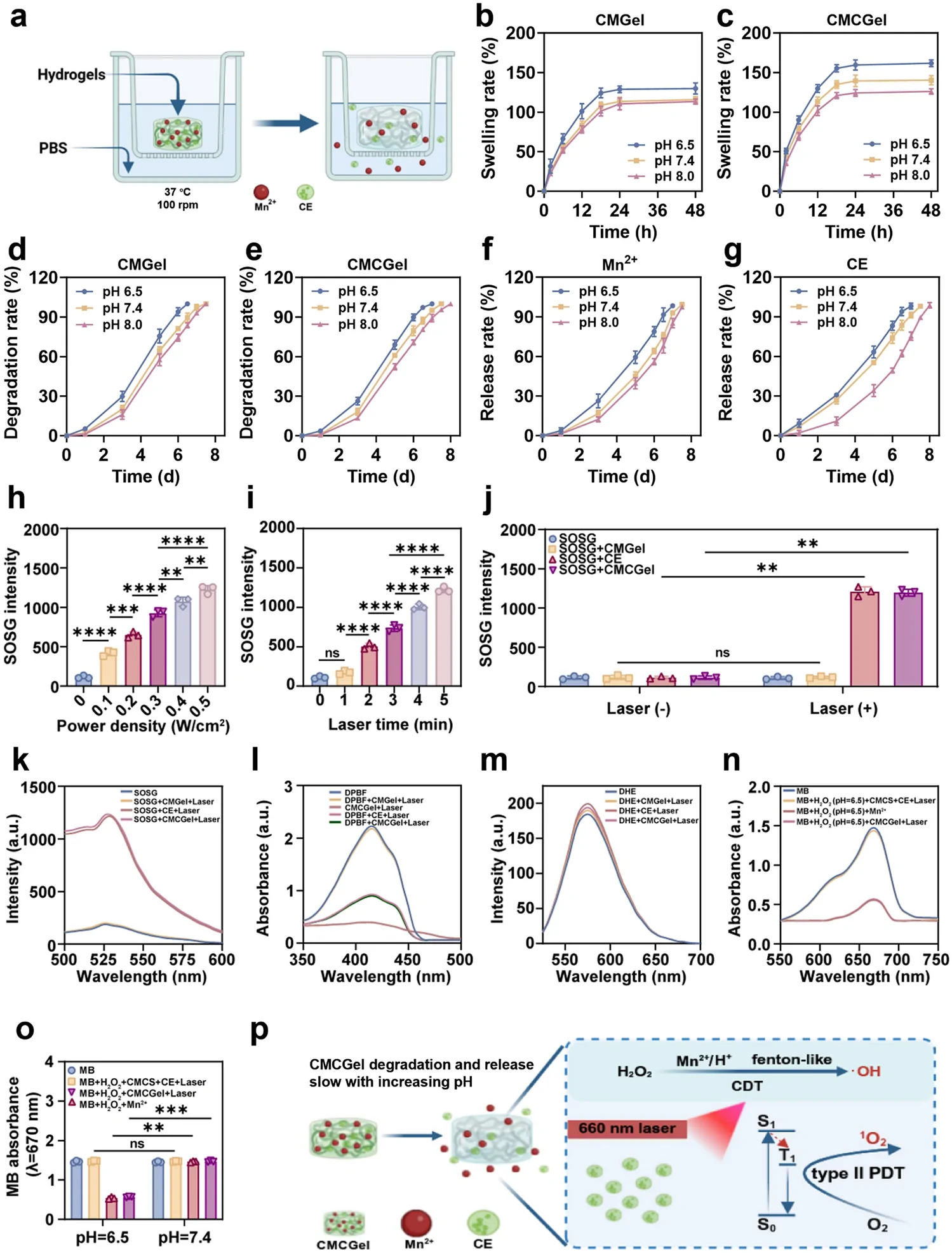
Dual pH/laser-responsive properties of CMCGel hydrogels. **a** Schematic diagram of swelling, degradation, and release behavior. Swelling curves of **b** CMGel and **c** CMCGel; Degradation curves of **d** CMGel and **e** CMCGel. Release curves of **f** Mn^2+^ and **g** CE. Fluorescence intensity of CMCGel with SOSG as a ^1^O_2_ probe at different laser **h** power densities and **i** intervention times. **j** Fluorescence intensity of the SOSG probe after treatment with different hydrogels ± laser (0.5 W cm^−2^, 5 min). **k** SOSG fluorescence spectrum, **l** DPBF absorption spectrum, **m** DHE fluorescence spectrum and **n** MB absorption spectrum after treatment with different hydrogels ± laser (0.5 W cm^−2^, 5 min). **o** Fluorescence intensity of the MB probe after treatment with different hydrogels ± laser (0.5 W cm^−2^, 5 min). **p** Schematic diagram of the degradation, release, and ROS generation mechanisms of CMCGel-mediated PDT and CDT. The data are presented as the means ± SDs (n = 3); *p < 0.05, **p < 0.01, ***p < 0.001, ****p < 0.0001

For hydrogel-based biomaterials designed for medical applications, properties such as swelling, degradation, and drug release serve as fundamental quality indicators [41, 43]. Upon implantation, hydrogels are required to rapidly absorb wound exudate to effectively seal wounds, including POPF [41]. They should subsequently be gradually degraded to accommodate new tissue growth while therapeutic agents are continuously released to eradicate residual tumors, modulate the immune microenvironment, and facilitate tissue repair [41, 43]. As shown in Fig. 2a, both hydrogels were immersed in PBS buffers at pH 6.5 (acidic TME mimetic), pH 7.4 (normal physiological mimetic), and pH 8.0 (POPF microenvironment mimetic), under constant temperature (37 °C) and orbital agitation (100 rpm). These conditions ensure a dynamic fluid environment, better replicating the physiological state of in vivo interstitial fluid. As depicted in Fig. 2b, c, all the hydrogels exhibited rapid liquid absorption and swelling within the initial 2 h, reaching equilibrium at 18 h. Importantly, the swelling rate increased with decreasing pH; at a pH of 6.5, the swelling rate of CMCGel reached 161.76% ± 4.34%, which was 1.15-fold and 1.28-fold greater than those observed in the groups at pH 7.4 and 8.0, respectively. The data in Fig. 2d, e show that the degradation rates of both hydrogels increase in a time-dependent manner and decrease with increasing pH. When day 5 was taken as a representative time point, the degradation ratio of CMCGel at a pH of 6.5 was 69.39% ± 3.58%, which corresponded to 1.14-fold and 1.33-fold greater than that at a pH of 7.4 and 8.0, respectively. This pH-dependent discrepancy was primarily attributed to the pH-susceptible nature of the COO–Mn–OOC coordination cross-linking architecture. Specifically, when the ambient pH exceeded the acid dissociation constant (pKa ~ 4.0) of the carboxyl groups in CMCS, progressive deprotonation of the carboxyl moieties occurred with increasing pH, resulting in a denser coordination network between carboxylate anions and Mn^2+^ via COO–Mn–OOC linkages [37]. Consequently, the susceptibility of the hydrogels to erosion by the liquid milieu diminished, thereby decelerating the degradation kinetics. Furthermore, at a pH of 8.0, the degradation rate of CMCGel was slower than that of CMGel. This phenomenon was attributable to the oxidation of polyphenolic constituents in CE to quinonoid structures under weakly alkaline conditions, which subsequently underwent Schiff base cross-linking with the amino groups of CMCS [23–25]. This secondary cross-linking reaction increased the cross-linking density of CMCGel, thereby retarding its degradation. As shown in Figs. 2f–g and S8, the release behavior of Mn^2+^ and CE is synchronized with the degradation kinetics of CMCGel and decreases with increasing pH. The underlying pH-responsive release mechanism was similar to the aforementioned degradation mechanism. The fundamental principle entailed that as the hydrogel matrix underwent progressive erosion by the liquid environment, the cross-linking structures dissociated incrementally [34–36], leading to the concomitant release of Mn^2+^ and CE. Notably, the pH-responsive degradation and release properties of CMCGel are expected to achieve a balanced dual function of local pancreatic cancer control and POPF repair during the perioperative period while reducing the risk of systemic toxicity.

ROS display a diverse spectrum of reactivities, from highly reactive to more gradual interactions. Notably, singlet oxygen (^1^O_2_), superoxide anion (O_2_•^−^), and ·OH have emerged as significant molecules in antitumor research because of their pronounced cytotoxic properties [23, 31, 44]. To assess the efficacy of CMCGel in generating these ROS through PDT and CDT, we employed four probes for parallel monitoring: singlet oxygen sensor green (SOSG, a ^1^O_2_ probe), 1,3-diphenylisobenzofuran (DPBF, a ^1^O_2_ probe), dihydroethidium (DHE, an O_2_•^−^ probe), and methylene blue (MB, a ·OH probe). With respect to the safe irradiation parameters of 0–0.5 W cm^−2^ and 0–5 min [23], the SOSG detection results (Fig. 2h, i) indicated a linear increase in fluorescence intensity for the CMCGel + laser group with increasing power density and time. Consequently, 0.5 W cm^−2^ and 5 min were established as the standard irradiation parameters for subsequent experiments. As illustrated in Fig. 2j, k, the baseline levels for each group remained constant in the absence of laser irradiation. However, upon laser exposure, the fluorescence intensity in the CE + laser and CMCGel + laser groups increased markedly, reaching values of 1210.74 ± 60.23 and 1195.82 ± 43.58, respectively. No significant difference was observed between the CMGel + laser and blank groups. These findings suggest that the CE effectively facilitates the ability of PDT to generate ^1^O_2_ within the safety parameters of laser irradiation without interference from CMCS or Mn^2+^. DPBF detection (Fig. 2l) confirmed that both CE and CMCGel generated ^1^O_2_ following laser exposure, resulting in notable probe consumption, whereas CMGel remained inactive. Furthermore, DHE detection results (Fig. 2m) indicated that neither the CE nor the hydrogel produced O_2_·^−^ postirradiation, thereby excluding potential interference from Type-I PDT byproducts.

We subsequently proceeded to evaluate the performance of CDT, specifically assessing ·OH production under various pH conditions via the MB + H_2_O_2_ system. As depicted in Fig. 2n, o, the absorbance levels across all the groups remained stable at pH 7.4. However, a significant decrease in the characteristic absorption of MB was observed exclusively in the Mn^2+^ and CMCGel groups at pH 6.5. This indicates that the Fenton-like reaction facilitated by Mn^2+^ is selectively activated, leading to the catalysis of ·OH generation from H_2_O_2_. In conclusion, as depicted in Fig. 2p, CMCGel successfully achieved a “photochemical” dual-modal ROS burst within a single system. The PDT mechanism involves the chlorophyll components in the CE, which possess a porphyrin structure that absorbs energy upon laser stimulation, undergoes electronic excitation, and subsequently transfers energy to oxygen molecules. This process ultimately catalyzes the production of cytotoxic ^1^O_2_ from molecular oxygen. Concurrently, the CDT mechanism involves Mn^2+^ participating in a Fenton-like reaction within an acidic environment, catalyzing the generation of ·OH from H_2_O_2_. The three-dimensional network of the hydrogel functions solely as a “solid-state warehouse” without interfering with the independent catalytic activities of CE and Mn^2+^, thereby ensuring the full retention of PDT-CDT efficacy.

We subsequently evaluated the biosafety of the CMCGel + laser treatment strategy (Fig. S9). As outlined in Fig. S9a, we assessed the cellular safety of CMCGel + laser treatment in HUVECs. The results of the cell counting kit-8 (CCK-8) assay (Fig. S9b) indicated that treatment with either the laser or CMCGel alone had negligible effects on cell viability. Notably, the incorporation of CE in combination with CMCGel significantly improved cell viability (*p* < 0.0001). Although the cell viability in the CMCGel + laser group was marginally lower than that in the CMCGel alone group (*p* < 0.0001), it remained significantly greater than that in the control group (*p* < 0.0001). Subsequent validation using calcein-AM/PI live/dead staining confirmed the superior cellular safety of CMCGel combined with laser treatment, as evidenced by a high percentage of viable cells (99.72% ± 0.08%) (Fig. S9c, d). In summary, the CMCGel and laser approaches exhibited outstanding biosafety for HUVECs. This can be attributed to the gentle laser parameters, the intrinsic safety of the CMCS and Mn^2+^ components, and the enhanced biocompatibility provided by the CE component.

CMGel and CMCGel were subsequently subcutaneously implanted into C57BL/6 J mice to assess their in vivo biosafety. The results of the in vivo degradation study indicated that both hydrogels were completely degraded in the mice by day 6, approximately one day earlier than observed in the in vitro PBS experiments (Fig. S10a). We hypothesize that this accelerated degradation is attributable to the catalytic hydrolysis of high-molecular-weight glucan chains in CMCS by endogenous glucanases present in the mice, in addition to phagocytic clearance by macrophages [38]. Furthermore, following a single local injection of CMCGel, we continuously monitored manganese levels in mouse blood, liver, and kidneys over a 28-day period using inductively coupled plasma‒optical emission spectrometry (ICP‒OES). The results revealed no significant differences compared with those in the PBS control group; the slight peak observed on day 7 was not statistically significant (Fig. S10b). This phenomenon is primarily ascribed to the local administration route, which precludes systemic exposure, and the sustained degradation and release profile of the hydrogel, which ensures localized Mn retention and minimal systemic distribution. Additionally, neither CMCGel nor CMCGel combined with laser irradiation induced significant hemolysis (Fig. S11a, b); no abnormal body weight fluctuations were observed over a 21-day period (Fig. S11c), and hematoxylin–eosin (H&E) staining of major organs (heart, liver, spleen, lung, and kidney) following euthanasia on day 21 revealed no pathological lesions (Fig. S11d). These findings collectively demonstrate that the CMCGel-mediated phototherapeutic strategy exhibits excellent biocompatibility, which is primarily attributed to the following factors: the intrinsic biosafety of individual constituents (CMCS, Mn^2+^, and CE) [23, 29, 38]; the local injectable administration and in situ release characteristics of CMCGel; the localized intervention and noninvasive nature of PDT, which offers high controllability in terms of power density, irradiation duration, and spatial precision; and the acidic TME-specific activation of CDT [17, 31].

In summary, the COO-Mn-OOC bridging structure of CMGel endows it with pH-responsive controllable degradation and release properties. Combined with its CDT initiated exclusively in the acidic TME and precisely controllable PDT, this system can simultaneously meet the dual requirements of local pancreatic cancer therapy and POPF repair during the perioperative period [16, 31, 44]. Notably, CMCGel integrates two orthogonal temporal control mechanisms: endogenous pH-gating governs degradation, release, and CDT kinetics—fast in an acidic TME for the ‘Remove’ phase and slow in neutral/alkaline environments for the ‘Remodel/Repair’ phases—while exogenous laser switching precisely turns PDT on and off as needed. This ‘pH-dictated rhythm, laser-determined precision’ design underpins the programmatic 3R strategy. Specifically, the porphyrin structure inherent in chlorophyll within the CE absorbs energy upon laser exposure and transfers it to O_2_, effectively generating cytotoxic ^1^O_2_ [23]. By precisely controlling the power, duration, and spatial distribution of laser irradiation, CMCGel can achieve targeted PDT for tumor treatment, thereby optimizing the protection of surrounding healthy tissues [17–19]. Mn^2+^ facilitates the conversion of endogenous H_2_O_2_ into highly toxic ·OH radicals through a Fenton-like reaction within the acidic TME, thereby achieving the combined antitumor effects of CDT and CE-PDT [31, 45]. This reaction, however, remains largely inactive in neutral to alkaline normal tissues, thereby minimizing off-target damage and achieving genuinely tumor-selective multimodal therapy [45]. Unlike previous materials that exhibit only singular antitumor [16] or POPF repair [14] functions, CMCGel integrates dual mechanisms: “laser/pH-triggered targeted PDT-CDT” and “physical sealing and healing promotion,” thereby offering both antitumor and tissue repair capabilities. Moreover, owing to its injectability, self-healing properties, tissue adhesion, biodegradability, and sustained release characteristics, CMCGel holds significant promise for clinical translation. It is anticipated to play a pivotal role in the comprehensive treatment of tumors and in soft tissue engineering.

### Antitumor Activity of the CMCGel Hydrogels In Vitro

**Fig. 3:**
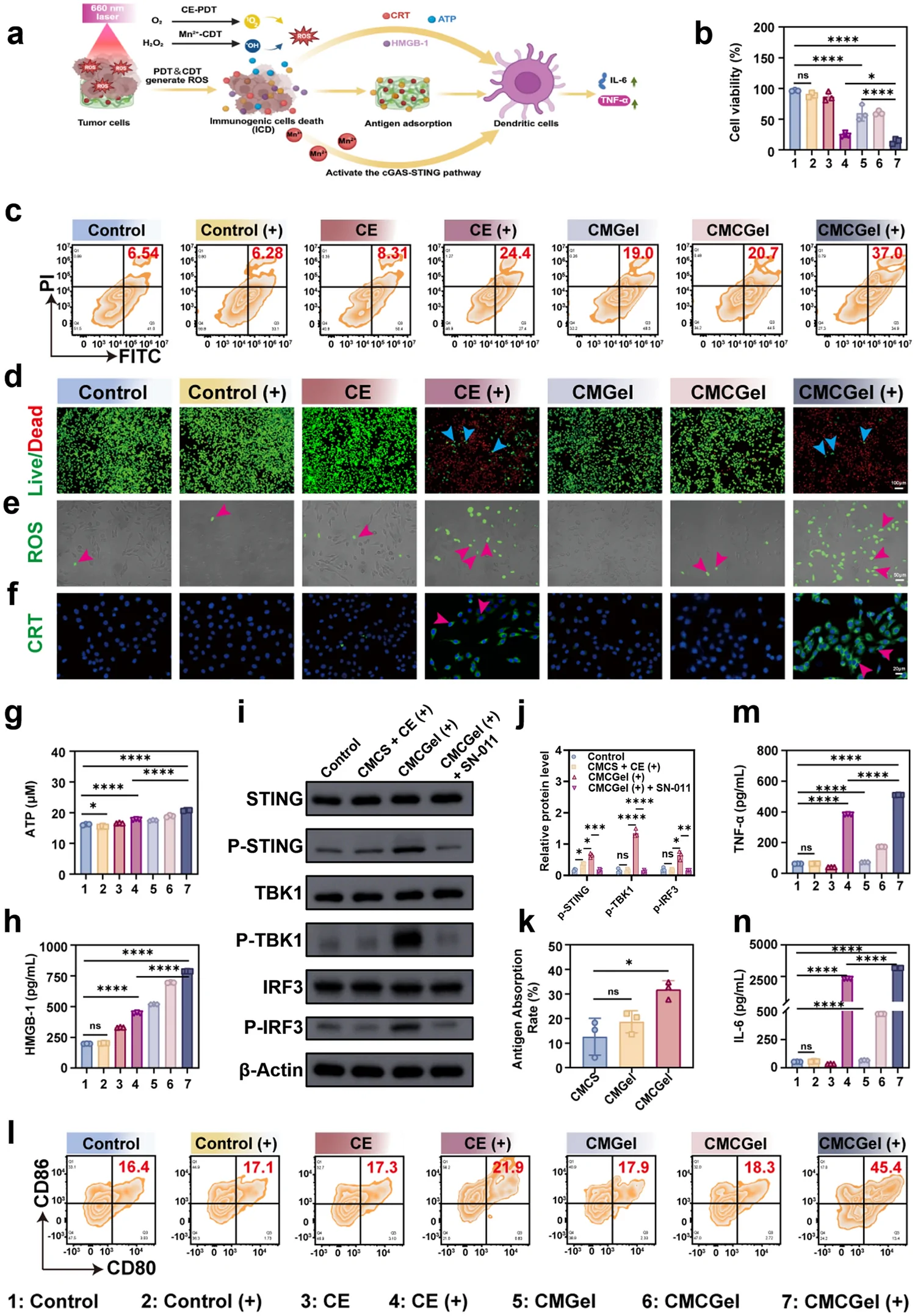
Antitumor activity of the CMCGel hydrogels in vitro. **a** Schematic diagram of the antitumor mechanism of CMCGel through PDT combined with CDT. **b** Cell viability. **c** Apoptosis (as measured by flow cytometry with Annexin V-FITC/PI double staining). **d** Live/dead cells (as measured by Calcin-AM/PI double staining fluorescence; scale = 100 µm). **e** Intracellular ROS levels (as measured by the DCFH-DA probe; scale = 50 µm). **f** CRT levels (as measured by immunofluorescence; scale = 20 µm). **g** ATP levels (as measured by chemiluminescence). **h** HMGB-1 levels (as measured by ELISA). **i-j** STING, p-STING, TBK1, p-TBK1, IRF-3 and p-IRF3 levels (as measured by WB). **k** Antigen adsorption test. **l** DC maturation (CD11c^+^ CD80^+^ CD86.^+^) was analyzed via flow cytometry. **m** TNF-α and **n** IL-6 levels were determined via ELISA. **b, g, h, m**, and **n**: 1 represents the control, 2 represents the control + laser, 3 represents the CE, 4 represents the CE + laser, 5 represents the CMGel, 6 represents the CMCGel, and 7 represents the CMCGel + laser. **c, d, e, f, i, j** and **l**: (+) indicate “+ laser.” The data are presented as the means ± SDs (n = 3); *p < 0.05, **p < 0.01, ***p < 0.001, ****p < 0.0001

The fundamental strategy for controlling tumor progression involves removing tumor cells and remodeling the antitumor immune microenvironment [46, 47]. As depicted in Fig. 3a, the CMCGel + laser system we developed collaboratively generates ROS through PDT and CDT, effectively eradicating tumor cells and inducing ICD, which facilitates the release of DAMPs and tumor antigens. Concurrently, the sustained release of Mn^2+^ from the hydrogel further activated the cGAS-STING signaling pathway. These mechanisms collectively enhance the maturation of DCs.

A CCK-8 assay was used to evaluate the effects of various treatments on the viability of the Panc02 cells. As shown in Fig. 3b, treatments involving either laser or CE alone had negligible effects on cell survival. Conversely, the combination of CE with a laser and the application of CMGel markedly reduced tumor cell viability (*p* < 0.0001), confirming the efficacy of CE-PDT and Mn^2+^-CDT as single modalities. CMCGel combined with laser treatment achieved a tumor inhibition rate of 85.81% ± 3.30%, underscoring the combined effects of PDT and CDT on tumor suppression. These findings were further substantiated through Annexin V-FITC/PI double staining coupled with flow cytometry, which revealed late apoptosis rates of 29.47% ± 4.63% and 24.71% ± 2.99% for the CE + laser and CMGel treatments, respectively, whereas CMCGel + laser treatment increased the late apoptosis rate to 41.03% ± 1.43% (Figs. 3c and S12). Additionally, calcein-AM/PI live/dead staining demonstrated a combined enhancing effect, with a tumor cell death rate of 97.61% ± 1.59% in the CMCGel + laser group, which was 1.19-fold and 50.58-fold greater than those observed in the CE + laser and CMGel groups, respectively (Figs. 3d and S13). A green fluorescent probe, 2′,7′-dichlorodihydrofluorescein diacetate (DCFH-DA), was used to quantify intracellular ROS levels to evaluate the efficacy of PDT and CDT. As shown in Fig. 3e, the fluorescence intensity of cells treated with either the laser or CE alone did not significantly differ from that of the control group. In contrast, the level of ROS production in the CMCGel + laser group was 52.17-fold greater than that in the control group, 3.28-fold greater than that in the CE + laser group, and 32.96-fold greater than that in the CMGel group (Fig. S14). These data indicate that the integration of PDT and CDT significantly enhances ROS production. In summary, these results illustrate that the combined application of CMCGel and laser treatment, through the combined ROS burst generated by PDT and CDT, effectively amplifies the cascade effect, resulting in the efficient eradication of Panc02 cells.

PDT and CDT can induce ICD, thereby transforming dying tumor cells into “endogenous vaccines” [23, 45]. This transformation is contingent upon the release of DAMPs by tumor cells, including calreticulin (CRT), adenosine triphosphate (ATP), and high-mobility group box 1 (HMGB-1), which collectively initiate antitumor immunity [23, 45]. Immunofluorescence analysis revealed that CRT exposure was most pronounced in the CMCGel + laser group, with a 10.05-fold increase compared with that in the control group, a 1.63-fold increase compared with that in the CE + laser group, and a 10.40-fold increase compared with that in the CMGel group (Figs. 3f and S15). Additionally, extracellular ATP levels were highest in the CMCGel + laser group, at 20.71 ± 0.11 μM, which represented 1.17-fold and 1.19-fold increases over those in the CE + laser and CMGel groups, respectively (Fig. 3g). Consistent with these findings, both the immunofluorescence analysis of HMGB-1 (Fig. S16) and the enzyme-linked immunosorbent assay (ELISA, Fig. 3h) results confirmed that HMGB-1 release was also greatest in the CMCGel + laser group. The data presented above confirm that the combination of CMCGel and laser treatment induces ICD by collaboratively generating a burst of ROS through PDT and CDT. This compromises tumor cell integrity and results in the release of a substantial amount of DAMPs. CRT functions as an “eat me” signal, ATP serves as a “find me” chemotactic factor, and HMGB-1 facilitates DC maturation and the secretion of proinflammatory cytokines [47]. These three components operate in a complementary manner, both spatially and temporally, to transform “cold” tumors into “hot” tumors, thereby fully activating the antitumor immune response [47].

Key strategies for enhancing DAMP-mediated DC antitumor immune responses include activating the cGAS-STING signaling pathway and adsorbing tumor antigens to improve their presentation efficiency [26, 29, 38]. To assess the potential of CMCGel to activate the cGAS‒STING pathway and adsorb antigens, we conducted a series of experiments. First, we detected that the concentration of Mn^2+^ in the cytoplasm of tumor cells in the CMCGel group was significantly greater than that in the control group and the CMCS + CE group (Fig. S17a, *p* < 0.001), indicating that the Mn^2+^ released by CMCGel could effectively penetrate and accumulate within tumor cells. Given that cytosolic dsDNA is a critical upstream activator of the cGAS-STING pathway [29], we next measured dsDNA levels under different treatment conditions (Fig. S17b). Compared with the control group, the CMCS + CE + laser (*p* < 0.0001), CMCGel (*p* < 0.05), and CMCGel + laser (*p* < 0.0001) groups all exhibited significantly elevated cytosolic dsDNA levels, with the highest levels in the CMCGel + laser group, followed by the CMCS + CE + laser group and then the CMCGel group. Notably, compared with the CMCGel group, the CMCGel + mannitol (a specific ·OH scavenger) group presented markedly reduced dsDNA levels (*p* < 0.05), directly demonstrating that the ·OH generated by Mn^2+^-mediated CDT is the key upstream event driving dsDNA release. Even in the presence of Mn^2+^, scavenging ·OH substantially attenuated dsDNA release, thereby establishing a direct causal link between the CDT function of Mn^2+^ and subsequent cGAS-STING activation. Western blot (WB) analysis further revealed the dual role of Mn^2+^ (Figs. 3i, j and S18). The CMCS + CE + laser group (PDT alone) exhibited mild activation of the cGAS-STING pathway (increased p-STING levels, *p* < 0.05), indicating that PDT alone can trigger this pathway through ROS-induced dsDNA release. In contrast, the CMCGel + laser group showed the strongest activation, with significantly elevated levels of p-STING (*p* < 0.001), p-TBK1 (*p* < 0.0001), and p-IRF3 (*p* < 0.05), demonstrating that the addition of Mn^2+^ markedly enhances the pathway response. Critically, pathway activation in the CMCGel + laser + SN-011 (a cGAS-STING pathway inhibitor) group was significantly suppressed (*p* < 0.001, *p* < 0.0001, *p* < 0.01), even though this group retained the ability to induce dsDNA release through PDT/CDT. These results unequivocally demonstrate that Mn^2+^ not only promotes dsDNA release through CDT but also acts as a direct agonist to synergistically activate the cGAS-STING pathway together with dsDNA; both functions are indispensable [26, 29]. Furthermore, as illustrated in Fig. 3k, CMCS, CMGel, and CMCGel demonstrated effective antigen adsorption capabilities, with CMCGel exhibiting the highest adsorption capacity at 31.76 ± 2.98 μg mg^−1^. This enhanced adsorption is attributed to the presence of free amino groups in the CMCS, which facilitate antigen adsorption through electrostatic interactions. Additionally, the catechol structure present in the CE significantly augments the antigen adsorption capacity of CMCGel [23, 24]. Collectively, these findings suggest that CMCGel not only effectively activates the cGAS-STING signaling pathway but also has exceptional tumor antigen absorption capabilities. These properties strongly support its potential to synergize with DAMPs to promote DC maturation, thereby enhancing antitumor immune responses [26, 29].

DAMPs and tumor antigens can interact with pattern recognition receptors (PRRs) expressed on the surface of DCs. In addition to the activation of the cGAS-STING signaling pathway by Mn^2+^, these interactions collaboratively trigger both innate and adaptive immune responses [26, 29]. Flow cytometry analyses (Figs. 3l and S19) revealed that, compared with those in the control group, the expression of CD80 and CD86 in the CE + laser and CMGel treatment groups was significantly upregulated (*p* < 0.0001, *p* = 0.0122). Notably, the highest expression level was observed in the CMCGel + laser group, reaching 44.27 ± 1.14% (*p* < 0.0001). These findings suggest that both PDT and CDT facilitate the maturation of DCs, with CMCGel further augmenting this maturation effect through combined mechanisms. Additionally, the levels of TNF-α and IL-6 secreted by DCs were assessed (Fig. 3m, n). Compared with those in the control group, treatments with either laser or CE alone did not result in significant alterations, whereas both the CE + laser and CMGel groups exhibited substantial increases (*p* < 0.0001). The most pronounced increase was observed in the CMCGel + laser group, where the TNF-α and IL-6 levels increased to 510.26 ± 0.92 and 3168.28 ± 0.97 pg mL^−1^, respectively, representing increases of 8.1-fold and 60.5-fold compared with those in the control group. In conclusion, the findings indicate that CMCGel, through the combined mechanisms of PDT and CDT, effectively induces ICD, facilitates the release of DAMPs and antigens, and activates the cGAS-STING signaling pathway. This activation subsequently promotes DC activation and the secretion of proinflammatory cytokines, specifically tumor necrosis factor-alpha (TNF-α) and interleukin-6 (IL-6). These processes collectively initiate a robust antitumor immune response, providing a substantial foundation for inhibiting tumor recurrence and metastasis.

In summary, in vitro studies have demonstrated that the combination of CMCGel and laser treatment effectively eradicates pancreatic cancer cells and induces robust ICD. The subsequent release of various DAMPs facilitates the maturation of DCs and the secretion of proinflammatory cytokines, thereby initiating a strong antitumor immune response that acts as an “immune firewall” to prevent postoperative recurrence and metastasis. Mechanistically, when activated by laser irradiation, the photosensitizer CE produces ^1^O_2_ via the PDT effect, directly destroying tumor cells and inducing ICD [23]. Mn^2+^ enhances the PDT effect through two pathways: First, it catalyzes the conversion of endogenous H₂O₂ into highly toxic ·OH via a Fenton-like reaction in the acidic tumor microenvironment, thereby combining CDT with PDT to generate a burst of ROS, which enhances the direct elimination of tumor cells [31, 45]. Second, Mn^2+^ activates the cGAS-STING signaling pathway, which, in conjunction with DAMPs released by ICD, further promotes DC maturation and enhances antitumor immunity [26, 29]. Furthermore, the CMCGel hydrogel, which is based on the CMCS backbone, has the capacity to effectively load and sustainably release CE and Mn^2+^. Additionally, it can adsorb tumor antigens via its free amino groups, thereby increasing antigen presentation efficiency and augmenting antitumor immunity [38]. This system integrates CE, Mn^2+^, and CMCS in a combined manner, achieving antitumor effects through multiple mechanisms that surpass the simple additive effects of the individual components. Its efficacy is significantly superior to that of conventional systems, which typically focus on either a single ROS burst or the activation of the cGAS-STING signaling pathway [26, 45]. Notably, upon optimizing component concentrations and laser parameters, CMCGel in combination with laser treatment effectively eradicated pancreatic cancer cells while maintaining favorable biocompatibility, underscoring the advantages of precise targeted antitumor therapy [23, 41]. In conclusion, as a hydrogel that integrates PDT, CDT, and cGAS-STING pathway activation therapy, CMCGel offers an innovative, safe, and effective clinical strategy for the comprehensive management of perioperative pancreatic cancer.

### Antitumor Activity of the CMCGel Hydrogels In Vivo

**Fig. 4:**
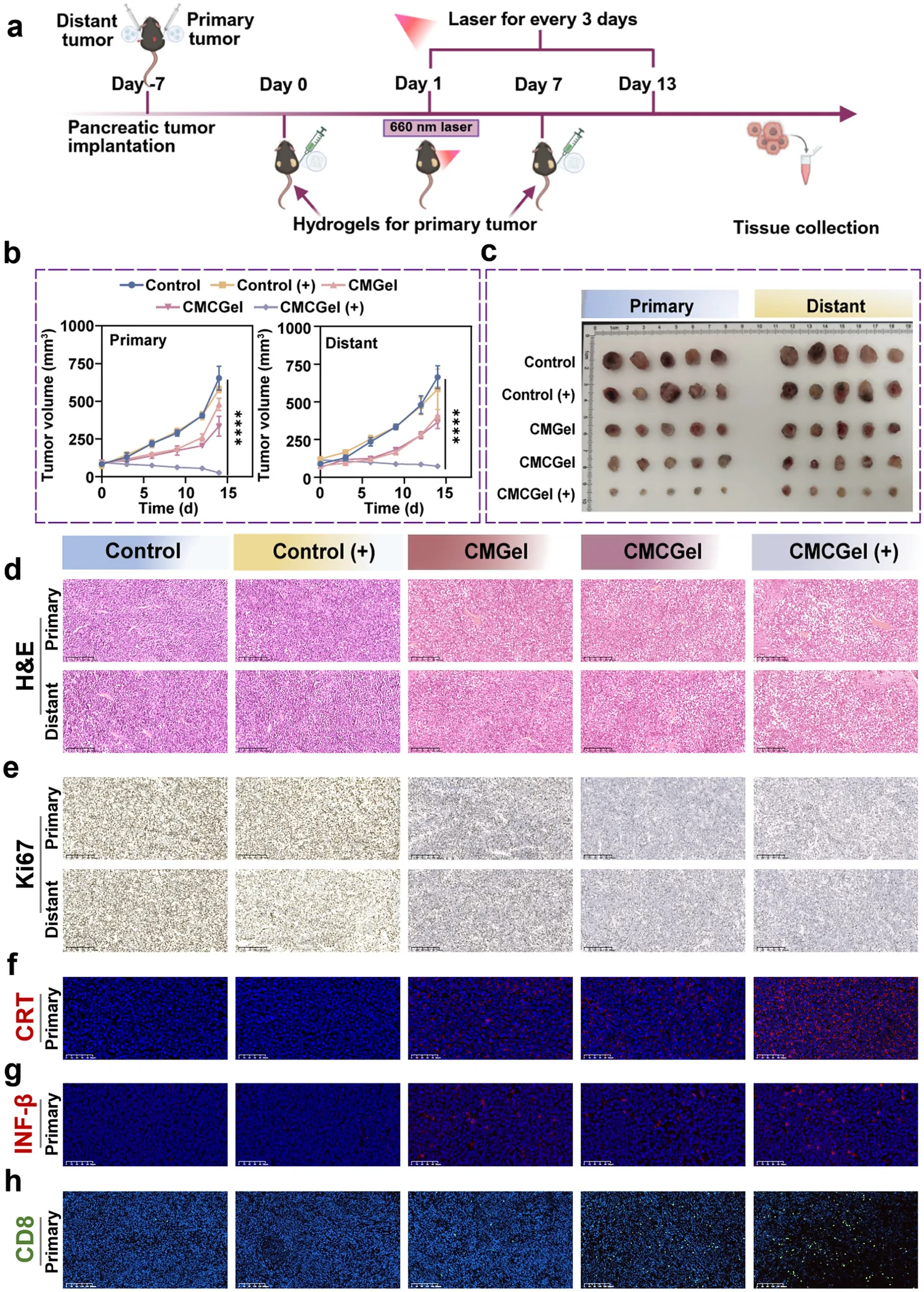
Antitumor activity of the CMCGel hydrogels in vivo. **a** Establishment and treatment of primary and distant pancreatic cancer models in C57BL/6J mice. Changes in **b** volume and **c** photographs of primary and distal tumors after different treatments. **d** H&E staining (scale = 100 µm), **e** Ki67 immunohistochemical staining (scale = 100 µm), **f** CRT immunofluorescence staining (scale = 50 µm), **g** INF-β immunofluorescence staining (scale = 50 µm) and **h** CD8 immunofluorescence staining (scale = 200 µm) of primary tumor tissues after different treatments. The data are expressed as the means ± SDs (n = 5); *p < 0.05, **p < 0.01, ***p < 0.001, ****p < 0.0001

To evaluate the efficacy of CMCGel combined with laser therapy in eradicating tumors and inducing systemic antitumor immunity, we established a bilateral Panc02 tumor-bearing C57BL/6 J mouse model. First, 5 × 10^6^ Panc02 cells were subcutaneously injected into the left and right sides of the mice. Once the tumor volume reached approximately 100 mm^3^, the right-sided tumor (primary tumor) received localized CMCGel injection every seven days and laser treatment every three days, while the left tumor (distant tumor) remained untreated as a control for assessing abscopal effects (Figs. 4a and S20). Tumor growth on both sides was monitored longitudinally, and immunohistochemistry and flow cytometry were performed to analyze local and systemic immune responses.

As shown in Figs. 4b and S21a, on the 14th day of tumor growth, tumor progression was significantly inhibited in the CMGel and CMCGel groups compared with that in the control group, with primary tumor inhibition rates of 26.57% ± 5.56% and 48.71% ± 9.09%, respectively. These findings further corroborate the efficacy of Mn^2+^-CDT. Upon administration of CMCGel in conjunction with laser therapy, the primary tumor volume inhibition rate increased to 96.01% ± 0.21%, underscoring the enhanced cascade amplification effect of PDT and CDT when used together. The primary tumor volume in the CMCGel + laser group was notably smaller than that in the untreated left region, and there was a marked reduction in the degree of tumor surface ulceration (Fig. S21b). Moreover, the final volumetric assessments of both primary and distant tumors (Fig. S21c, d) revealed that the tumor inhibition rates in the CMCGel combined with laser treatment group were 94.38% ± 2.42% and 88.36% ± 4.22%, respectively. Macroscopic images of bilateral tumors in mice further corroborated the potential application of CMCGel in postoperative adjuvant therapy (Fig. 4c).

To elucidate the combined antitumor mechanisms of CMCGel in vivo, we performed comprehensive histological and immunohistochemical analyses on treated tumor tissues. First, we examined the changes in the intratumoral Mn concentration following peritumoral injection of the hydrogel into primary tumors. The results revealed that the manganese concentration gradually increased and peaked within 14 days (Fig. S22), directly demonstrating that manganese can effectively accumulate locally in tumors and maintain a sufficient concentration, thereby providing the material basis for its CDT and activation of the cGAS-STING pathway. On the basis of the effective accumulation of Mn, we further evaluated the direct cytotoxic effects of the treatment. H&E staining demonstrated that the tumor tissues on the treated side in the CMCGel + laser group exhibited extensive necrosis, characterized by typical nuclear shrinkage and cytoplasmic dissolution, along with significant tumor cell ablation and the formation of void areas (Fig. 4d), which intuitively reflects the potent direct cytotoxic effects induced by the combined action of PDT and CDT. Further evaluation of tumor cell proliferation through Ki67 staining revealed a reduction in the Ki67-positive index in the CMCGel + laser group (Fig. 4e), suggesting that this treatment not only effectively eradicates existing tumor cells but also persistently inhibits the proliferation of residual cells, thereby preventing tumor recurrence. In the context of ICD induction, the application of CMCGel combined with laser treatment resulted in a significant increase in the exposure of CRT on the surface of tumor cell membranes (Fig. 4f) and a marked increase in the release of HMGB-1 (Fig. S23). These findings collectively confirm that this therapeutic approach effectively initiates the ICD process, facilitating the conversion of tumor cells from an immunologically “cold” state to a “hot” state. Furthermore, a notable increase in the expression of interferon-β (INF-β) was detected in the CMGel, CMCGel, and CMCGel + laser groups (Fig. 4g), indicating the successful activation of type I interferon pathways. These observations further suggest that the cGAS‒STING signaling pathway, which is mediated by Mn^2+^, plays a pivotal role in modulating innate immune responses. Analysis of immune cell infiltration revealed a significant increase in the density of CD8⁺ T cells within the tumor tissues of the CMCGel + laser group (Fig. 4h), demonstrating that CMCGel combined with laser treatment not only induced localized tumor cell destruction but also effectively activated systemic antitumor immunity. This activation promotes the chemotaxis and infiltration of effector T cells to the tumor site.

**Fig. 5:**
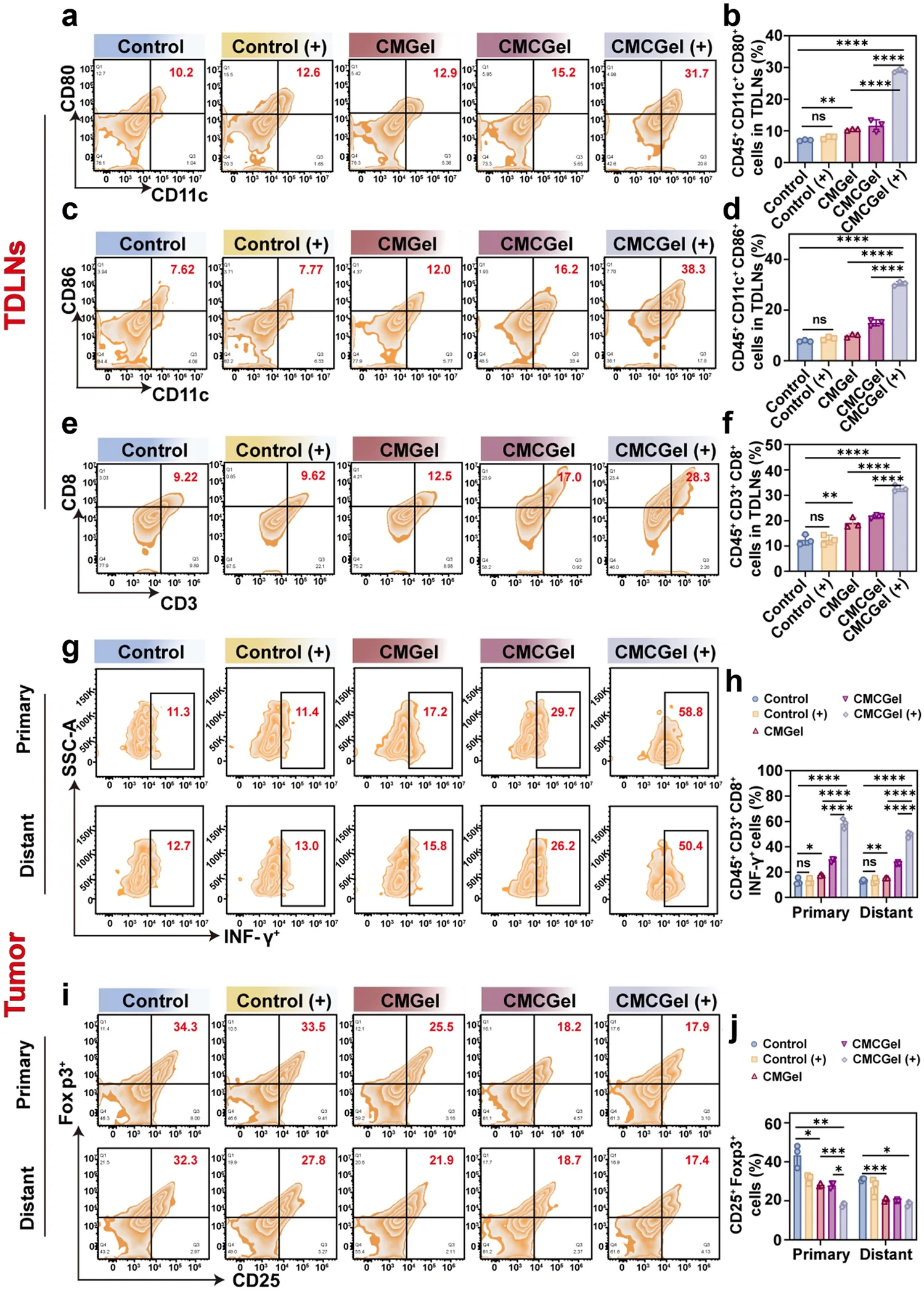
Remodeling the antitumor immune microenvironment of the CMCGel hydrogels in vivo. Flow cytometry analysis of **a-d** DCs (CD45^+^ CD11c^+^ CD80^+^, CD45^+^ CD11c^+^ CD86^+^) and **e–f** CD8^+^ T cells (CD45^+^ CD3^+^ CD8^+^) in TDLNs after different treatments. Flow cytometry analysis of **g–h** TIEs (CD45^+^ CD3^+^ CD8^+^ IFN-γ^+^) and **i-j** Tregs (CD25^+^ Foxp3.^+^) in primary and distant tumor tissues of mice following different treatments. The data are expressed as the means ± SDs (n = 3); *p < 0.05, **p < 0.01, ***p < 0.001, ****p < 0.0001

To systematically assess the efficacy of CMCGel combined with laser treatment in modulating the immune microenvironment and eliciting systemic antitumor immunity, we initially employed flow cytometry to analyze DC maturation and T-cell activation within tumor-draining lymph nodes (TDLNs). The maturation of DCs is a critical step in the initiation of T-cell responses, as it effectively enhances T-cell activation through the upregulation of costimulatory molecules, thereby facilitating systemic antitumor immunity [46, 47]. As shown in Fig. 5a–d, compared with the control, CMGel increased the proportions of CD45^+^CD11c^+^CD80^+^ and CD45^+^CD11c^+^CD86^+^ cells by 1.26-fold and 1.57-fold, respectively. The CMCGel combined with laser treatment further increased these proportions to 31.70% ± 0.39% and 38.31% ± 0.66%, respectively. These findings suggest that CMGel promotes DC maturation through Mn^2+^-mediated CDT and activation of the cGAS-STING signaling pathway, in addition to antigen capture and retention by the free amino groups of the CMCS backbone. The incorporation of CE-PDT significantly enhances the performance of CMCGel in conjunction with laser treatment during this process. Concurrently, an analysis of cytotoxic T lymphocyte (CTL) infiltration (Fig. 5e, f) revealed that the percentage of CD45^+^ CD3^+^ CD8^+^ cells was 19.10% ± 1.64% in the CMGel group, whereas it increased to 32.73% ± 1.01% in the CMCGel + laser group. These findings indicate that CMGels facilitate CTL infiltration by activating the CDT and cGAS-STING signaling pathways, as well as through antigen capture, to improve presentation. The addition of CE-PDT further amplified this effect.

To comprehensively evaluate the immune-modulating capacity of CMCGel combined with laser treatment, we performed an in-depth analysis of CTLs, tumor-infiltrating effector T cells (TIEs) and regulatory T cells (Tregs) within the TME. CTLs play a critical role in tumor eradication, and the abundance of TIEs—which reflect CTL infiltration into tumor sites—directly indicates antitumor immune efficacy [23, 24]. In contrast, Foxp3^+^ Tregs suppress TIE infiltration, and their reduction signifies attenuated immune suppression [23, 24]. The detection results of CTLs (CD45^+^CD3^+^CD8^+^, Fig. S24a, b) and TIEs (CD45^+^CD3^+^CD8^+^INF-γ^+^, Fig. 5g, h) revealed that the infiltration of CTLs and TIEs in the control group was minimal, whereas that in the CMGel group significantly increased for both CTLs and TIEs in primary and distant tumors (*p* < 0.05). In mice treated with CMCGel combined with laser therapy, the proportions of CTLs in primary and distant tumors reached 31.42% ± 0.16% and 26.25% ± 0.37%, respectively, which were 1.40-fold and 1.57-fold greater than those in the CMGel group. Moreover, the proportions of TIEs significantly increased to 58.37% ± 3.16% and 49.57% ± 1.85%, representing increases of 3.31-fold and 3.21-fold, respectively, compared with those in the CMGel group. These results demonstrate that CE-PDT substantially enhances the ability of CMGel to promote CTL and TIE infiltration. Moreover, compared with the control group, the CMCGel + laser group had the lowest proportion of Tregs, with reductions of 52.18% ± 1.57% and 53.87% ± 2.13%, respectively. In summary, the CMCGel + laser strategy effectively reversed the immunosuppressive TME by depleting organic acids and H_2_O_2_ via COO‒Mn‒OOC bonds and Mn^2^⁺-mediated Fenton-like reactions. Furthermore, this treatment activated the cGAS-STING pathway through Mn^2+^ release, captured and retained antigens via free amino groups, and triggered a ROS burst through PDT-CDT synergy. Consequently, it efficiently eliminated tumor cells, reshaped the antitumor immune microenvironment, and initiated a systemic immune response, thereby suppressing tumor recurrence and metastasis.

To distinguish between abscopal effects and immune memory, we designed a tumor “treatment-resection-rechallenge” experiment (Fig. S25a) on the basis of the study by Chao et al. [48]. Notably, we modified the experimental protocol by shortening the time interval for tumor rechallenge. Among these groups, Group 1 was used to verify whether simply tumor growth followed by resection is sufficient to induce protective immune memory; Group 2 served to exclude interference from nonspecific factors such as surgical trauma and anesthesia procedures on immune responses; and Group 3 was the core experimental group and was used to test whether the immune memory induced by CMCGel + laser treatment could persist long term without drug maintenance and effectively defend against new tumor attacks. As shown in Fig. S25b, c, from the left-sided tumor rechallenge on day 20 to the end of the observation period on day 28, the left-sided tumors in group 1 (tumor resection control) and group 2 (sham surgery) exhibited parallel rapid growth trends, with no significant differences in tumor volume at any time point between the two groups (*p* > 0.05). These results indicate that neither simple tumor resection nor surgical procedures alone are sufficient to establish effective systemic immune surveillance capable of defending against new tumor attacks; without an effective immune activation strategy, tumor resection may even lose its protective effect because of the persistence of an immunosuppressive microenvironment [13, 14]. In contrast, left-sided tumor growth in Group 3 (CMCGel + laser treatment) was significantly slower than that in the two control groups (*p* < 0.01). By the experimental endpoint on day 28, the tumor volume was 145.15 ± 30.10 mm^3^ in Group 1 and 107.86 ± 22.28 mm^3^ in Group 2, whereas Group 3 had a volume of only 40.34 ± 36.03 mm^3^. Compared with that in Group 1, the tumor inhibition rate in Group 3 was 75.18% (*p* < 0.0001) (Fig. S25d, e). Importantly, during Days 20–28, Group 3 received no CMCGel injections or laser irradiation, and the tumor inhibitory effect relied entirely on the immune memory established during the treatment period from Days 7–18. These results confirm that CMCGel + laser treatment can induce durable antitumor immune memory independent of continuous drug exposure.

In conclusion, the results of in vitro and in vivo experiments collectively demonstrate that CMCGel combined with laser irradiation effectively eliminates pancreatic cancer cells, induces ICD, promotes DC maturation, and activates T cells, thereby reprogramming the immune microenvironment and triggering a systemic antitumor immune response. Mn^2+^ plays a dual role in tumor clearance and immune modulation: within the acidic TME, it catalyzes the conversion of endogenous H_2_O_2_ into highly toxic ·OH via a Fenton‑like reaction, directly killing tumor cells and inducing ICD [31]; simultaneously, it activates the cGAS–STING signaling pathway, which together with DAMPs released during ICD further enhances DC maturation and antitumor immunity [29]. Upon laser activation, the CE generates ^1^O_2_ through PDT, resulting in a burst of ROS that amplifies tumor cell elimination and immune microenvironment remodeling [23]. Moreover, the CMCS-based hydrogel framework enables efficient loading and sustained release of both CE and Mn^2+^, while its free amino groups adsorb tumor antigens, thereby improving antigen presentation and augmenting antitumor immunity [38]. The combination of CMCGel with laser treatment thus has potent antitumor effects and holds considerable promise for the comprehensive management of pancreatic cancer. By collaboratively generating ROS via PDT/CDT and activating the cGAS-STING pathway with Mn^2+^, this strategy reduces the risk of drug resistance—a major limitation of conventional therapies [26, 45]. Furthermore, the spatially precise application of laser irradiation minimizes off‑target damage to surrounding healthy tissues [23].

### Pancreatic Fistula Repair Activity of the Hydrogels

To evaluate the potential of CMCGel to promote POPF healing, we first examined its effects on the proliferation and migration of rat pancreatic stellate cells (RPSTCs). Pancreatic stellate cells (PSCs) are the core effector cells involved in pancreatic injury repair, orchestrating extracellular matrix (ECM) remodeling and tissue regeneration [41]. As shown in Fig. S26a, compared with the control treatment, CMGel treatment did not significantly affect RPSTC viability at any time point. In contrast, CMCGel significantly promoted RPSTC proliferation, increasing cell viability by 1.33-fold after 72 h of treatment (*p* < 0.01), indicating that the incorporation of CE (rich in polyphenols, proteins, and other bioactive substances) is essential for pancreatic-specific cellular regeneration [23–25]. Scratch migration assays (Fig. S26b, c) further revealed that compared with the control group, the CMCGel-treated group achieved a cell confluence rate of 65.71% ± 3.25% at 24 h, representing a 4.18-fold increase. This enhanced migratory capacity suggests that CMCGel not only supports stellate cell survival but also effectively promotes their directed migration toward the injury site, a critical process for tissue remodeling and fistula closure [41]. Additionally, we evaluated the effects of CMCGel on the proliferation and migration of HUVECs. As shown in Fig. S27a, CMCGel significantly promoted HUVEC proliferation at 48 and 72 h (*p* < 0.0001). Concurrently, CMCGel accelerated HUVEC migration, achieving migration rates of 44.71% ± 0.31% and 68.04% ± 1.33% after 12 and 24 h of treatment, respectively, which were significantly greater than those of the control group (12 h: 13.41% ± 5.98%; 24 h: 33.22% ± 6.87%; Fig. S27b, c). These results confirm that CMCGel accelerates endothelial cell recruitment to the injury site, thereby actively contributing to the healing process [21, 25]. In summary, CMCGel exerts dual pro-regenerative effects on PSCs and endothelial cells, thereby establishing a coordinated cellular basis for ECM remodeling, neovascularization, and functional tissue repair during the critical postoperative phase of POPF healing [21, 25, 41].

**Fig. 6:**
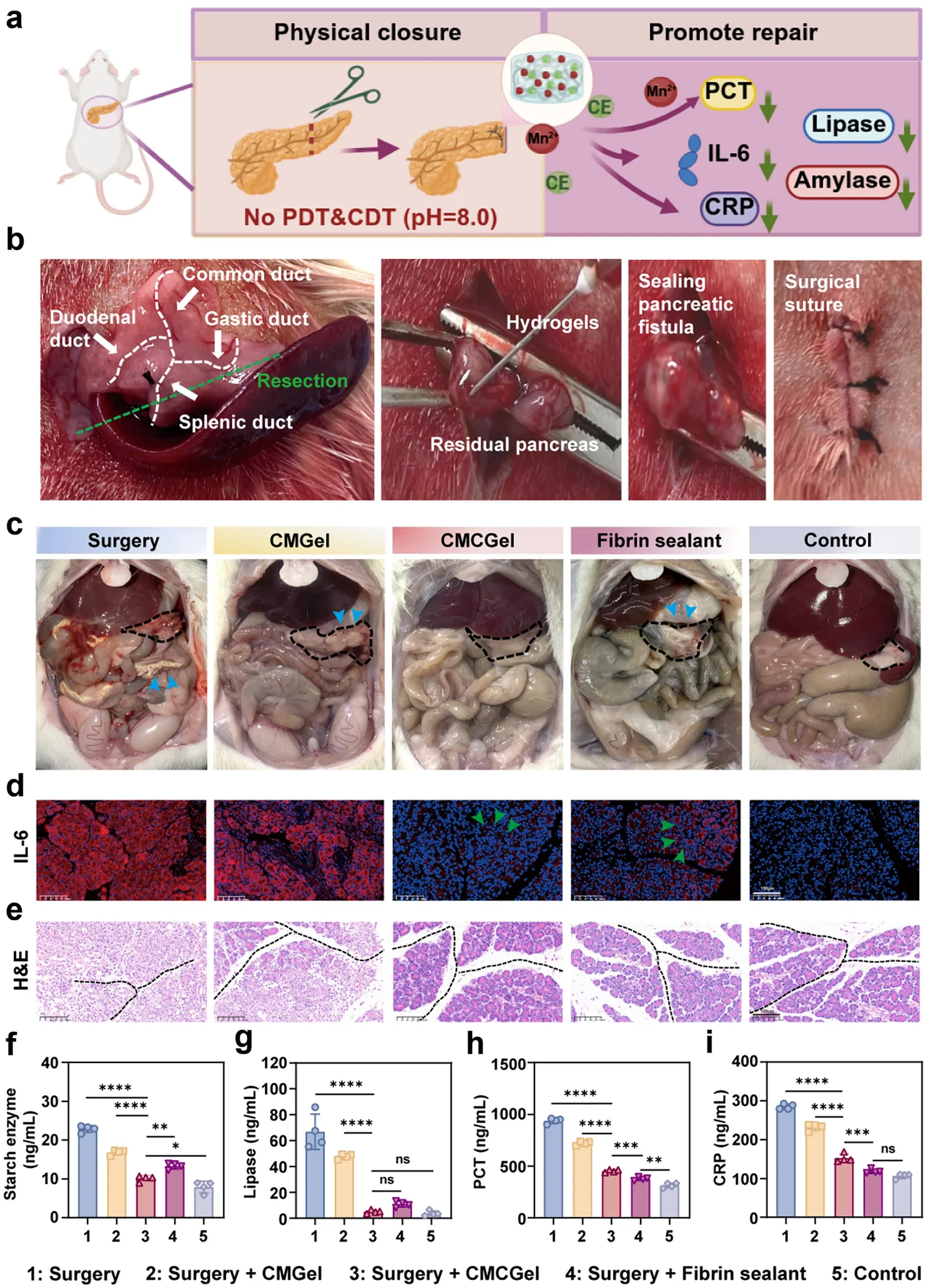
POPF repair activity of the CMCGel hydrogels. **a** Mechanistic diagram of the CMCGel hydrogel for POPF repair. **b** Establishment and treatment of a POPF model in SD rats. The photographs clearly labeled the surgical incision site (subcostal incision in the upper left abdomen), the range of distal pancreatectomy, the hydrogel injection site (remnant stump of the pancreas), and the processes of the pancreatic stump suture and incision suture. **c** Anatomical photograph of the abdominal organs. **d** IL-6 immunofluorescence (scale bars = 100 μm) and **e** H&E staining images (the black dashed line indicates the direction of the pancreatic duct; scale bars = 100 μm) of the pancreatic fistula remnant. **f** Amylase and **g** lipase levels in the peritoneal lavage fluid. **h** PCT and **i** CRP levels in the serum. The data are expressed as the means ± SDs (n = 4); *p < 0.05, **p < 0.01, ***p < 0.001, ****p < 0.0001

To confirm the repair-promoting effects observed in vitro, we established a rat model of POPF to evaluate the in vivo repair efficacy of CMCGel. Fibronectin, a standard agent for the prevention of postoperative pancreatic fistula, was selected for inclusion in one of the experimental groups. This group was further divided into the following subgroups: surgery only, surgery with CMGel, surgery with CMCGel, surgery with fibrin sealant, and a control group that underwent laparotomy alone. The surgical procedure involved transection of the pancreatic parenchyma and the adjacent splenic duct to mimic clinical pancreatic fistula formation, after which the hydrogel was applied to the residual pancreatic tissue for therapeutic intervention (Fig. 6a). During surgery, microsurgical suturing (7–0 Prolene) was carefully performed to achieve precise vascular ligation, thereby minimizing trauma-related confounding effects (Fig. 6b). Postoperative monitoring revealed no overt signs of systemic toxicity, as indicated by stable body weights and normal histology of major organs in H&E-stained sections (Fig. S28a, b). These findings confirm the favorable in vivo biocompatibility of CMCGel, supporting its potential for clinical translation.

On postoperative day 48, anatomical examination revealed extensive intra-abdominal adhesions and prominent saponification spots in the surgery group. Adhesion was moderately reduced in the CMGel and fibrin sealant groups, yet localized saponification spots remained. In contrast, the CMCGel-treated group displayed no detectable leakage or adhesion, with the integrity of the wound site closely resembling that of the control group (Fig. 6c). These observations indicate that CMCGel provides effective mechanical sealing and markedly suppresses postoperative adhesion formation, highlighting its superior barrier function. Analysis of local inflammatory responses via IL-6 immunofluorescence staining further revealed a significantly lower density of IL-6^+^ cells in the CMCGel group than in both the CMGel and fibrin sealant groups (Fig. 6d), suggesting that active components such as CE may attenuate inflammatory factor expression and thereby reduce local inflammation. H&E staining revealed a disorganized pancreatic duct architecture, extensive neutrophil infiltration, and tissue necrosis in the surgery group. Although inflammation was reduced in the CMGel group, the structural alterations persisted. Conversely, the CMCGel group exhibited well-preserved pancreatic duct morphology with near-normal cellular architecture (Fig. 6e). Collectively, these histopathological findings indicate that CMCGel comprehensively promotes fistula closure and structural restoration, establishing a conducive microenvironment for postoperative tissue regeneration.

Amylase and lipase levels in peritoneal lavage fluid are key markers for assessing POPF severity [41]. Compared with the surgery group, the CMCGel group had significantly lower levels of lipase (92.43%) and amylase (55.71%) (Fig. 6f, g), indicating its high efficacy in preventing pancreatic enzyme leakage and alleviating associated biochemical abnormalities. Notably, the reduction in lipase achieved by CMCGel was 24.01% greater than that in the fibrin sealant group, demonstrating superior sealing performance relative to this clinically established agent. In contrast, the CMGel group exhibited only modest decreases in lipase (28.53%) and amylase (26.32%), underscoring the essential contribution of CE to repair efficacy.

In addition to local enzymatic indicators, systemic inflammatory responses were evaluated to further delineate the anti-inflammatory profile of CMCGel. Serum analysis revealed that the procalcitonin (PCT) and C-reactive protein (CRP) levels in the CMCGel group were 453.8 ± 11.30 and 152.50 ± 9.30 ng mL^−1^, respectively, which were significantly lower than those in the surgery group (*p* < 0.0001; Fig. 6h, i). These values correspond to reductions of 51.60% (PCT) and 46.53% (CRP), substantially exceeding the reductions observed in the CMGel group (22.97% and 18.55%, respectively), which further confirms the critical role of CE in mediating anti-inflammatory synergy.

Histopathological scoring provided additional evidence at the tissue level. Adhesion scores in the CMCGel group were 94.74% lower than those in the surgery group—which markedly surpassed the 26.32% reduction seen with CMGel—indicating nearly complete suppression of postoperative adhesion formation (Fig. S29a, c). Similarly, pancreatitis activity scores (Fig. S29b, d) were reduced by 75% in the CMCGel group, which was significantly greater than that in the fibrin sealant group (*p* = 0.0001). Together, these findings demonstrate that CMCGel effectively mitigates pancreatic inflammatory damage and establishes a local microenvironment conducive to wound healing.

This study focuses on the “repair” objective of the 3R strategy and systematically evaluates the reparative effect of the CMCGel hydrogel on POPF. Biologically, the incorporated CE plays a central role [20–25]: In vitro assays revealed that CMCGel effectively promoted the proliferation and migration of RPSTCs, which play a central role in extracellular matrix remodeling and pancreatic tissue repair, as well as in HUVECs, thereby establishing a coordinated cellular basis for extracellular matrix reconstruction, neovascularization, and functional tissue regeneration. In vivo experiments further confirmed that the hydrogel markedly suppressed local IL-6 expression and reduced the expression of systemic inflammatory markers—specifically PCT and CRP—by more than 45%, demonstrating its ability to modulate postoperative inflammation and optimize the repair microenvironment. With respect to mechanical repair, the three-dimensional network formed through Mn^2+^-CMCS coordination endows the hydrogel with superior sealing properties. In animal models, CMCGel adhered robustly to the wound site, leading to a 92.43% reduction in lipase levels and a 55.71% reduction in amylase levels in peritoneal lavage fluid. Furthermore, the adhesion score decreased by 94.74%, indicating effective inhibition of tissue adhesion and pancreatic juice leakage and confirming the reliable physical barrier function of this material. In summary, CMCGel not only modulates inflammation and promotes tissue repair via bioactive CE components but also accomplishes mechanical wound closure through its dynamically cross-linked network. This dual capacity—integrating biological repair with physical barrier reconstruction in the complex postoperative setting—represents a novel and translationally promising strategy for the comprehensive management of POPF after pancreatic cancer surgery.

### Transcriptomic Sequencing Analysis and Mechanistic Validation of CMCGel-Induced POPF Repair

**Fig. 7:**
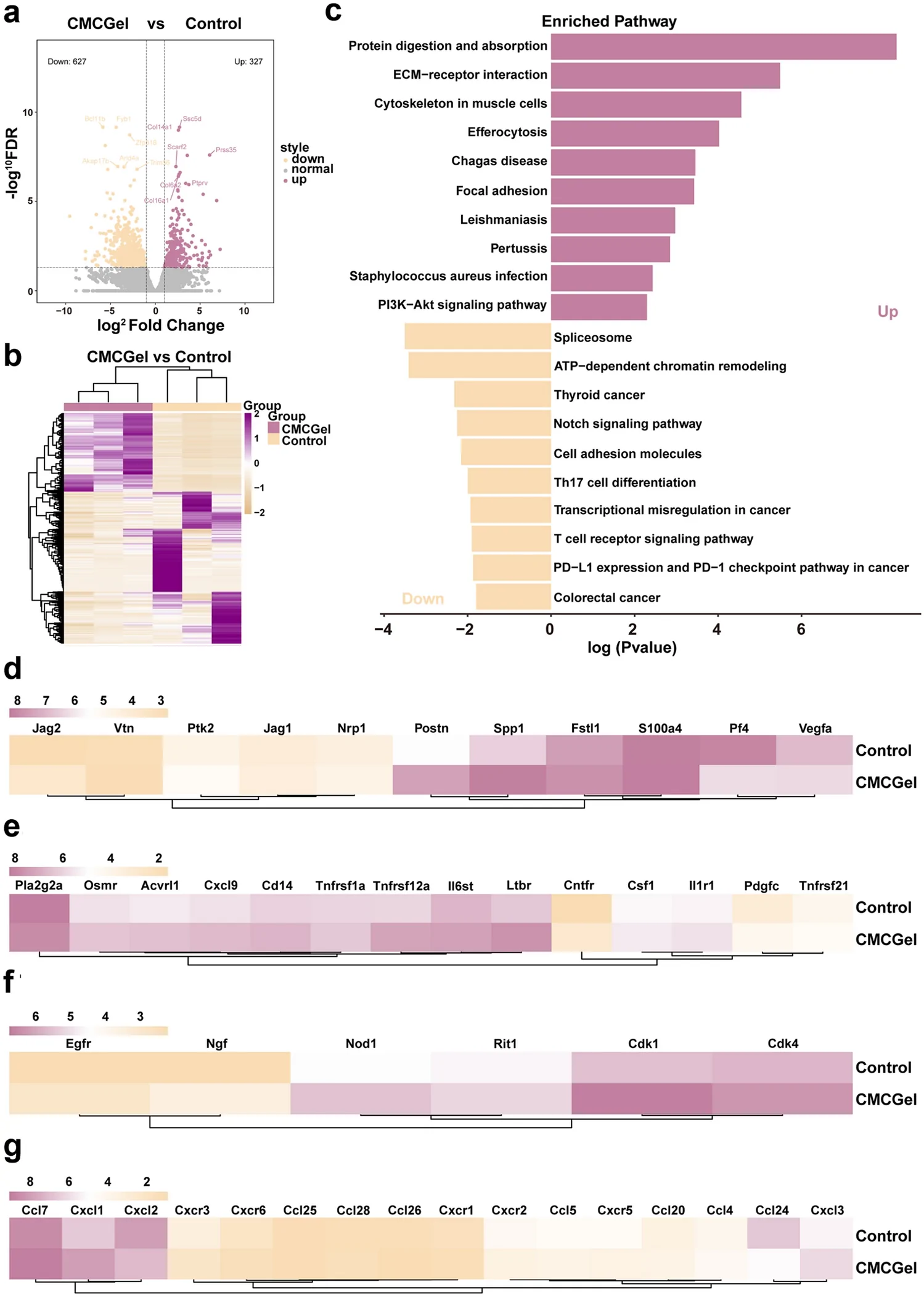
Transcriptome sequencing of the CMCGel hydrogels for in vivo pancreatic fistula repair. Differentially expressed genes (DEGs): **a** Volcano plot, **b** clustering heatmap, and **c** enrichment pathways. Gene expression related to **d** angiogenesis pathway, **e** PI3K‒Akt signaling pathway, and **f** JAK‒STAT signaling pathway. **g** Cytokine expression. The data are expressed as the means ± SDs (n = 3)

To elucidate the molecular mechanisms through which CMCGel modulates inflammatory responses and facilitates tissue repair in vivo, we performed RNA sequencing on residual pancreatic tissues from the POPF rat model and compared the CMCGel group with the surgery group. Applying the screening criteria (|log_2_(fold change)|> 1, FDR < 0.05), we identified 957 differentially expressed genes (DEGs), including 327 upregulated and 630 downregulated genes (Fig. 7a, b). Conversely, a number of upregulated genes are known to perform anti-inflammatory or repair-promoting functions. For example, the serine protease Prss35 can directly cleave and inactivate the proinflammatory chemokine CXCL2 [50]; collagen type XIV (Col14a1), a key structural component of the ECM, promotes ECM remodeling and stabilization upon upregulation [50]; and increased Scarf2 expression has been linked to the amelioration of inflammatory diseases, suggesting a tissue‑protective role [51]. Collectively, these transcriptomic changes indicate that CMCGel specifically activates gene programs related to inflammation suppression and ECM remodeling, providing initial mechanistic insights into its tissue repair capability at the transcriptional level.

We next performed Gene Ontology (GO) functional enrichment analysis to explore the biological significance of the DEGs. As shown in Fig. S30a, pathways associated with ECM organization, including extracellular structure organization, collagen fibril organization, collagen trimer formation, and extracellular matrix binding, were prominently enriched. These terms directly reflect processes essential for ECM assembly, collagen fiber formation, and the establishment of mechanical support [52]. The GO results thus demonstrated that CMCGel treatment robustly activated biological processes linked to ECM construction and tissue structural integrity, further corroborating its role in promoting tissue repair.

To elucidate the pathway-level mechanism underlying these biological processes, we performed Kyoto Encyclopedia of Genes and Genomes (KEGG) enrichment analysis on the DEGs (Figs. 7c and S30b). The results revealed coordinated activation of multiple pathways related to anti-inflammatory and repair-promoting functions. Notably, the focal adhesion pathway was upregulated, enhancing cell–matrix adhesion and thereby promoting tissue–interface maturation and stability [53]. The protein digestion and absorption pathway were elevated, with particular factors contributing to the optimization of protein metabolism and the facilitation of tissue repair. Furthermore, the ECM‒receptor interaction pathway was significantly enriched; its upregulation facilitates bidirectional signaling between the ECM and cells, playing a key regulatory role in tissue repair [54]. Collectively, the results of the KEGG analysis indicate that the reparative effects of CMCGel are not driven by a single pathway but through the integrated regulation of interrelated signaling axes, including cell‒matrix interactions that collaboratively drive the repair process.

Subsequent pathway analysis using the Reactome database yielded consistent findings (Figs. 7c and S30c). Major activated pathways included those involved in dynamic ECM organization [55], balanced collagen metabolism, and integrin-mediated signaling. The results of the analysis also suggested the involvement of immune regulatory pathways, such as interactions between lymphoid and nonlymphoid cells, along with enrichment of immune checkpoint-related signals. Thus, the results of the Reactome analysis not only corroborate the central role of CMCGel in ECM remodeling from another perspective but also highlight its ability to concomitantly modulate the immune microenvironment. Together, these pathway-level insights provide a more comprehensive framework for understanding the integrated “repair-remodeling” strategy orchestrated by CMCGel.

Using RNA-seq analysis of residual pancreatic tissue in the POPF rat model, we investigated the potential signaling pathways and networks modulated by CMCGel in the context of tissue repair and the inflammatory response. CMCGel treatment markedly influenced the expression of several key genes associated with angiogenesis, intracellular signaling, and immune regulation. Specifically, with respect to angiogenesis (Fig. 7d), the expression of proangiogenic genes, including Postn, Jag2, Fgfr1, and Spp1, was significantly upregulated in the CMCGel group, whereas the expression of the angiogenesis-inhibiting factor Pf4 was downregulated. This gene expression profile indicates that CMCGel may facilitate tissue repair by activating proangiogenic pathways, enhancing endothelial cell proliferation and migration, and expediting vascular reconstruction during the tissue repair process [56]. Concurrently, genes associated with the PI3K-AKT signaling pathway, such as Ltbr and Pdgfc, were also significantly upregulated in the CMCGel group (Fig. 7e). The PI3K-AKT signaling pathway serves as a fundamental regulatory mechanism for cell survival, proliferation, and metabolism, and it also facilitates angiogenesis and tissue regeneration, thereby providing the molecular foundation necessary for injury repair [57]. In terms of immune regulation, treatment with CMCGel significantly influenced the expression of genes associated with the JAK-STAT signaling pathway (Fig. 7f). The upregulation of genes such as Adcy2, Pitx2, and Ap2m1 indicates moderate activation of this pathway, which is involved in cell proliferation and immune regulation [58]. Furthermore, the expression levels of various cytokines and chemokines, including Ccl20, Ccl24, Cxcr5, Ccl5, and Cxcr3, were reduced in the CMCGel group (Fig. 7g), suggesting that CMCGel may attenuate local inflammatory responses associated with POPF by inhibiting the chemotaxis and activation of inflammatory cells, thus creating a conducive microenvironment for tissue repair [59].

**Fig. 8:**
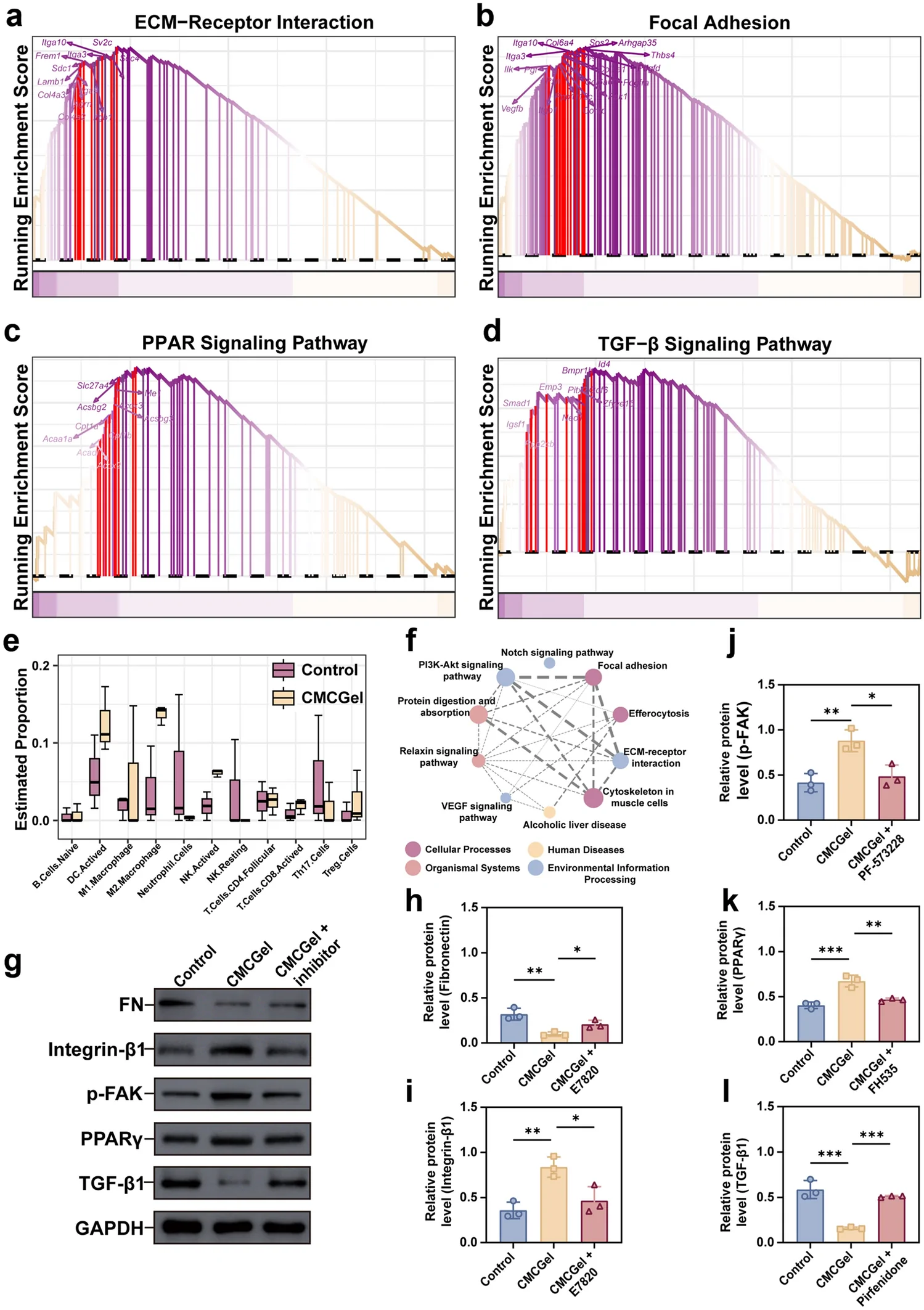
Transcriptomic analysis and experimental validation of CMCGel-regulated signaling pathways. **a–d** GSEA results indicating that the CMCGel group exhibited activation of several pathways: **a** ECM‒receptor interaction, **b** focal adhesion, **c** PPAR signaling pathway, and **d** TGF-β signaling pathway. **e** CIBERSORT analysis, which reveals the relative proportions of various cell types within the overall gene expression data. **f** KEGG network diagram highlighting entries with a *p* value of less than 0.05 selected from the upregulated KEGG results. In this network diagram, the thickness of the lines correlates with the number of overlapping genes, whereas the size of the nodes corresponds to the number of genes enriched in each entry. **g–l** FN, integrin-β1, p-FAK, PPARγ and TGF-β1 levels (as measured by WB). The data are expressed as the means ± SDs (n = 3). *p < 0.05, **p < 0.01, ***p < 0.001, ****p < 0.0001

To comprehensively evaluate the genome-wide impact of CMCGel on gene expression, we performed gene set enrichment analysis (GSEA) (Fig. 8a–d). The results showed that CMCGel treatment broadly activated gene sets associated with repair processes. These include key tissue repair pathways, such as those involved in ECM–receptor interactions, focal adhesion, TGF-β signaling, and PPAR signaling [60]. Notably, the *protein digestion and absorption* pathway, which is closely linked to pancreatic exocrine function, was also significantly upregulated. At the whole-genome level, GSEA robustly demonstrated that CMCGel concurrently suppressed inflammation-related pathways and activated a comprehensive reparative transcriptional program. This program promotes ECM remodeling, enhances cell survival, and facilitates the recovery of tissue function. Moreover, pronounced synergy was observed between anti-inflammatory and repair-related gene modules [61].

While the preceding pathway analyses focused predominantly on molecular functions, tissue repair ultimately depends on functional changes in specific cell populations. To this end, we applied the CIBERSORT algorithm to deconvolute the immune cell infiltration landscape in pancreatic tissues (Fig. 8e) [62]. In control animals, the microenvironment was dominated by proinflammatory M1 macrophages, neutrophils, and Th17 cells. In contrast, CMCGel treatment reshaped the microenvironment toward a reparative state, characterized by the presence of anti-inflammatory M2 macrophages and Treg cells, together with increased infiltration of activated DCs, natural killer (NK) cells, and CD8^+^ T cells. Thus, CIBERSORT-based deconvolution confirmed that CMCGel converts a proinflammatory immune landscape into a reparative landscape centered on M2 macrophages and Tregs [63]. This cellular-level shift aligns precisely with the immune-modulatory patterns predicted by earlier pathway analyses, thereby establishing a coherent evidence chain spanning transcriptional changes, pathway activation, and, ultimately, functional remodeling of the immune microenvironment.

To integrate the key signaling pathways identified above and visualize their functional interplay, we constructed a pathway network based on significantly upregulated KEGG entries (*p* < 0.05) (Fig. 8f). The network highlights several central hubs, including the efferocytosis pathway, which is responsible for clearing apoptotic cells; the PI3K–Akt signaling pathway, which promotes cell survival and proliferation; and the focal adhesion pathway, which is essential for cell anchoring and tissue integrity [64]. This analysis revealed that CMCGel likely activates a coordinated signaling network centered on PI3K-Akt, which functionally links efferocytosis with cell–matrix adhesion. Such integration supports tissue homeostasis by removing damaged debris while reinforcing structural architecture, thereby systematically driving anti-inflammatory and reparative processes.

To validate the transcriptomic sequencing results at the protein level, this study utilized passage 2 RPSTCs to examine the changes in the expression of key proteins in four major signaling pathways identified by GSEA using WB analysis (Fig. 8g). Furthermore, specific pathway inhibitors were used to verify the regulatory effects of CMCGel on these pathways. The inhibitors used included E7820 (which inhibits fibronectin (FN) and integrin-β1), PF-573228 (which inhibits focal adhesion kinase (FAK) kinase activity), pirfenidone (which inhibits TGF-β1), and FH535 (which inhibits peroxisome proliferator-activated receptor gamma (PPARγ)). As shown in Fig. 8h, compared with the control treatment, treatment with CMCGel significantly downregulated the expression of FN, a key glycoprotein in the ECM-receptor interaction pathway, by 69.28% (*p* = 0.0033). Combined treatment with E7820 further reduced the FN level (*p* < 0.05), suggesting that E7820 synergistically enhances the inhibitory effect of CMCGel on the FN. The downregulation of FN may attenuate ECM-integrin signaling, inhibit collagen deposition, and thereby exert an antifibrotic effect [65]. In the focal adhesion pathway (Figs. 8i, j and S31), CMCGel treatment significantly upregulated the expression of integrin-β1 and phosphorylated FAK (p-FAK) by 134.17% (*p* = 0.0075) and 111.30% (*p* = 0.0067), respectively. The addition of PF-573228 markedly suppressed the increase in p-FAK (*p* < 0.01), indicating that FAK kinase activity is a critical step in the activation of this pathway, which may promote cell adhesion, migration, and tissue repair [65]. Furthermore, as shown in Fig. 8k, PPARγ expression increased by 65.93% (*p* = 0.0008) following CMCGel treatment. Cotreatment with FH535 significantly blocked this upregulation (*p* < 0.01), confirming that the regulatory effect of CMCGel on PPARγ may be associated with its role in inhibiting inflammation and promoting reparative macrophage polarization [66]. Conversely, as shown in Fig. 8l, after CMCGel treatment, TGF-β1 expression decreased by 73.21% (*p* = 0.0002). The addition of pirfenidone further reduced TGF-β1 levels (*p* < 0.05), indicating that pirfenidone enhances the inhibitory effect of CMCGel on the TGF-β pathway, which helps suppress fibroblast activation and ECM deposition, thereby exerting a synergistic antifibrotic effect [67]. These protein expression changes are consistent with the GSEA enrichment analysis results. Combined with validation using specific inhibitors, these findings confirm the involvement of multiple signaling pathways and elucidate the molecular mechanism through which CMCGel mediates anti-inflammatory, prorepair, and antifibrotic effects through the coordinated regulation of these pathways.

In summary, through transcriptomic profiling and multidimensional bioinformatics approaches, this study systematically elucidated the molecular mechanisms through which CMCGel promotes tissue repair and modulates inflammation in a postoperative pancreatic fistula model. Transcriptomic analysis revealed specific upregulation of genes linked to anti-inflammatory responses and ECM remodeling (e.g., Prss35 and Col14a1). GO and KEGG enrichment analyses further revealed that CMCGel robustly activated structural-repair pathways, including those involved in ECM organization and focal adhesion, while also implicating epigenetic regulators such as chromatin-remodeling factors. GSEA corroborated that CMCGel both dampens excessive immune activation signatures and induces repair-associated gene modules. At the cellular level, immune deconvolution demonstrated a shift from a proinflammatory milieu dominated by M1 macrophages toward a reparative state enriched with M2 macrophages and regulatory T cells. Signaling network analysis further indicated that CMCGel enhances cell survival and angiogenesis while suppressing inflammatory chemotaxis, principally through modulation of the PI3K-Akt axis, proangiogenic genes, and the JAK-STAT pathway. The results of the WB protein validation align with the transcriptomic data, indicating an upregulation of critical proteins within the focal adhesion pathway, specifically Integrin-β1 and phosphorylated FAK, alongside a downregulation of proteins associated with fibrosis, such as fibronectin and TGF-β1. In conclusion, this study provides a comprehensive knowledge of the integrated molecular mechanisms through which CMCGel facilitates tissue repair and regeneration. This is achieved through the coordinated regulation of extracellular matrix remodeling, anti-inflammatory signaling, and reprogramming of the immune microenvironment, as evidenced by a multitiered chain of evidence encompassing genes, pathways, cells, and proteins.

### 3R Programmatic Treatment of CMCGel: Implications for Pancreatic Cancer and POPF Management

Perioperative management of pancreatic cancer has long faced a central dilemma: how to effectively eradicate tumors while preserving pancreatic function and promoting postoperative healing [3, 14, 15]. Traditional strategies often view antitumor therapy and tissue repair as conflicting objectives—aggressive tumor treatment is accompanied by tissue damage, whereas repair alone cannot address residual tumor burden [14, 15]. CMCGel achieves the programmed integration of antitumor activity and tissue repair through a sequential dual-regulatory mechanism: endogenous pH-gating autonomously regulates the macrorhythm of CDT and degradation/release, while exogenous laser switching precisely controls the on/off of PDT. Their synergy establishes the spatiotemporal logic of the 3R strategy.

The core design of CMCGel utilizes a pH/laser dual-responsive mechanism to achieve programmatic switching of therapeutic modalities. Importantly, this switching does not rely on arbitrary time windows but is governed by two orthogonal control layers. In the acidic TME, the cross-linked COO-Mn-OOC structure undergoes dissociation, accelerating the release of Mn^2+^ and CE; when combined with external laser irradiation, the system simultaneously initiates PDT and CDT, generating bursts of ROS, including ^1^O_2_ and ·OH, directly disrupting tumor cell structures and inducing apoptosis and necrosis. Throughout this process, the dual functionality of ROS is particularly critical: its cytotoxic effects achieve efficient tumor clearance while triggering ICD to release DAMPs, and Mn^2+^ further activates the cGAS-STING signaling pathway, collectively promoting DC maturation and CTL infiltration, laying the foundation for subsequent immune microenvironment remodeling. Upon tumor elimination, the local pH normalized to ~ 7.4, which automatically slowed hydrogel degradation and Mn^2+^ release (Fig. 2d–g) and decreased CDT activity (Fig. 2n, o). At this stage, Mn^2+^ primarily activates the cGAS-STING pathway (Fig. 3i, j), enabling an automatic transition from direct antitumor activity to immune activation without human intervention. Upon termination of laser irradiation and transition to neutral-to-weakly alkaline environments, CMCGel exhibits distinctly different biological behaviors: the COO-Mn-OOC cross-linking structure remains stable, the Fenton-like reactivity of Mn^2+^ significantly decreases, and ROS generation nearly ceases; polyphenolic compounds in CE then exert anti-inflammatory and antifibrotic effects to achieve POPF repair. This laser-switched controlled ROS generation pattern not only ensures maximal efficacy during the tumor clearance phase but also achieves precise protection of normal pancreatic tissue, avoiding nonspecific damage to pancreatic tissue associated with traditional chemotherapy or sustained ROS release systems. Notably, the injectability of CMCGel enables precise delivery to the operative field through minimally invasive approaches, while its excellent self-healing properties and anti-pancreatic enzyme stability (81.98% mass retention at 48 h) ensure durable sealing effects in the complex pancreatic environment. Thus, the degradation, release, and CDT activity of CMCGel are endogenously gated by changes in the tumor and fistula microenvironments, resulting in a programmed macroscopic transition from tumor elimination to immune remodeling and then to fistula repair, whereas exogenous laser switching provides precise human control over the initiation and termination of PDT. This dual “endogenous rhythm, exogenous precision” strategy ensures that immune transition occurs not only through the passage of time but also through specific microenvironmental cues and changes in the level of ROS.

Another key innovation of CMCGel lies in achieving sequential remodeling of the immune microenvironment through a single platform, a process exemplifying the pivotal role of “Remodel” in connecting “Remove” and “Repair.” Critically, Mn^2+^ not only facilitates dsDNA release via CDT but also serves as a direct cGAS-STING agonist, creating a positive feedback loop that amplifies innate immune activation. During the tumor treatment phase, the system establishes a Th1-dominant antitumor immune microenvironment through ICD induced by combined PDT-CDT and Mn^2+^-mediated cGAS-STING pathway activation. In this phase, TIEs infiltration in tumor tissues significantly increased to 58.37%, whereas the proportion of Tregs decreased by 52.18%. This “hot tumor” immune state, characterized by high ROS levels and robust cytotoxic T-cell activity, is crucial for perioperative tumor control. Upon entering the POPF repair phase (laser OFF, neutral/alkaline pH, and notably, when ROS levels decrease because of both the termination of PDT and the automatic silencing of CDT), CMCGel actively shifts the immune microenvironment toward a favorable repair state through the anti-inflammatory and antifibrotic functions of CE. RNA sequencing revealed that POPF residual tissues treated with CMCGel exhibited significant immune phenotype conversion, with increased proportions of anti-inflammatory cells and significant upregulation of multiple prorepair genes, including Prss35, Col14a1, and Scarf2. Critically, this immune transition is not a passive consequence of time but is actively driven by a reduction in ROS levels (as the laser is turned off and the pH normalizes) and the dominant effect of the polyphenolic compounds of CE, which upregulate the activity of the PPAR signaling pathway and downregulate the expression of proinflammatory chemokines. This dynamic remodeling of the immune microenvironment enables the natural transition from antitumor immune responses to tissue repair phases, avoiding mutual interference between the two processes.

We fully recognize the concern that a prorepair environment could provide a survival advantage to any residual tumor cells. CMCGel addresses this risk through a three-tier defense mechanism. First-tier: maximized elimination of the primary tumor. As shown in Fig. 4b, CMCGel + laser inhibited the primary tumor by 96.01%, indicating an extremely low residual burden. Second tier: durable systemic antitumor immune memory. Even during the repair phase (laser OFF, neutral/alkaline pH), previously established antitumor immune memory continues to function. Untreated distal tumors on the opposite side achieved 88.36% inhibition (Fig. 4b), and tissue-infiltrating effector T cells remained detectable in the late stage (Fig. 5g, h). Moreover, a rechallenge model resulted in 75.18% tumor inhibition (Fig. S25e), confirming sustained immune surveillance. Third tier: physical barrier and isolation effect of the hydrogel. CMCGel covers the surgical wound and fistula site, forming a physical barrier that isolates any potential residual tumor cells from prorepair immune signals, preventing them from gaining a survival advantage. These three tiers collectively ensure that the prorepair environment does not promote residual tumor outgrowth.

In-depth analysis of the mechanism of action of CMCGel revealed that antitumor and prorepair processes do not occur in isolation but achieve organic integration through dynamic interactions between therapeutic factor (e.g., ROS) levels and immune states, further corroborating the unity of the 3R strategy. During the tumor clearance phase, high levels of ROS serve not only as direct weapons but also as critical signals for immune initiation. ROS produced by combined PDT and CDT induce ICD in tumor cells to release DAMPs, Mn^2+^ activates the cGAS-STING pathway to amplify type I interferon responses, and the hydrogel captures antigens—these mechanisms collectively provide the necessary conditions for establishing antitumor immunity. To prevent sustained ROS and chronic inflammation from impeding POPF repair or even leading to pathological outcomes such as fibrosis, CMCGel actively reduces ROS levels after tumor clearance through spatiotemporal laser control, resulting in a transition to a repair phase dominated by anti-inflammatory effects and ECM remodeling. Polyphenolic compounds in CE play important roles in this phase, promoting M2 macrophage polarization and tissue-reparative inflammatory responses through activation of the PPARγ signaling pathway. Transcriptomic analysis revealed that CMCGel significantly upregulated the expression of PI3K-Akt signaling pathway-related genes (Ltbr and Pdgfc) and proangiogenic genes (Postn, Jag2, Fgfr1, and Spp1) but downregulated the expression of proinflammatory chemokines (Ccl20, Ccl24, Cxcr5, Ccl5, and Cxcr3), thereby molecularly confirming its ability to promote tissue repair through the coordination of cell survival, vascular regeneration, and inflammation suppression. This precise transition from “proinflammatory antitumor” to “anti-inflammatory prorepair” represents the concentrated manifestation of mutual influence within the 3R strategy.

The 3R programmatic therapeutic strategy of CMCGel provides a novel paradigm for the perioperative management of pancreatic cancer. In traditional treatment, the initiation of adjuvant tumor therapies (e.g., chemotherapy and radiotherapy) is often delayed and difficult to reconcile with POPF prevention [13, 14]; conversely, POPF management cannot achieve tumor control [41]. Through spatiotemporally controllable programming of a single platform, CMCGel achieves simultaneous fulfillment of both objectives: prioritizing tumor clearance and antitumor immune activation in the early perioperative period and subsequently transitioning naturally to POPF sealing and tissue repair without requiring treatment regimen changes or additional patient burden. By employing the dual “endogenous pH gating and exogenous laser switching” mechanism—where the pH dictates the macroscopic rhythm and the laser determines local precision—CMCGel provides clear and justifiable temporal logic for the 3R strategy. The immune transition is not arbitrary but is driven by specific microenvironmental cues (pH changes and reduced ROS levels) and the intrinsic properties of the components (Mn^2+^ switching from a ROS catalyst to a cGAS-STING activator; the anti-inflammatory polyphenols of CE). Furthermore, the three-tier defense mechanism (maximized primary tumor elimination, durable systemic immune memory, and physical barrier isolation) effectively mitigated the potential risk of residual tumor progression during the prorepair phase. This “antitumor–pancreas preservation” balance is rooted in a profound understanding of the essential differences between the pancreatic TME and postoperative repair microenvironments. Moreover, free amino groups in the CMCS backbone confer CMCGel with antigen capture and retention capabilities, both enhancing antitumor immune responses and alleviating excessive inflammatory reactions through clearance of necrotic tissue antigens, creating favorable conditions for tissue repair. In summary, through a pH/laser dual-responsive spatiotemporally controllable design, CMCGel achieves precise temporal control of ROS levels and immune microenvironment states on a single platform, thereby organically integrating antitumor immune remodeling and prorepair tissue regeneration—two seemingly independent processes—providing an important theoretical foundation and experimental basis for the development of next-generation integrated tumor therapeutic strategies.

Despite the definitive efficacy of CMCGel in terms of antitumor activity and tissue repair, its clinical translation still faces several limitations. First, the adhesion performance of the hydrogel in complex microenvironments requires further optimization to meet the high standards of clinical wound dressings. Second, sterilization processes in large-scale production, interbatch consistency, and long-term storage stability are critical issues that need to be resolved before clinical translation. Third, although rat models can meet basic experimental requirements, they cannot fully replicate the complexity of the human pancreatic cancer microenvironment (such as enzymatic activity and inflammatory responses) and human metabolic pathways. Therefore, in future translational research on CMCGel, more systematic and in-depth evaluation is needed to determine its adhesiveness, stability after scaling up, in vivo degradation kinetics and distribution characteristics, and acute and chronic toxicological responses. In the future, by optimizing the therapeutic efficacy of CMCGel, elucidating its molecular mechanisms, and ensuring its biosafety, the clinical translation process of CMCGel in the treatment of pancreatic cancer and other gastrointestinal diseases can be promoted.

## Conclusions

In response to the clinical challenges associated with perioperative pancreatic cancer progression and POPF, we developed CMCGel, an injectable self-healing hydrogel that implements a programmed “Remove**–**Remodel**–**Repair” (3R) strategy. To address concerns regarding temporal control and immune balance, CMCGel employs a dual “endogenous pH gating and exogenous laser switching” mechanism. In the acidic TME (pH ~ 6.5), laser activation generates a synergistic ROS burst via CE-PDT and Mn^2+^-CDT, resulting in potent tumor ablation (96.01% in vivo) and the induction of ICD. As the pH normalizes, Mn^2+^ shifts from CDT to cGAS-STING activation, promoting DC maturation and CD8^+^ T-cell infiltration. Upon laser termination in an alkaline POPF environment (pH ~ 8.0), ROS generation ceases, and CE exerts anti-inflammatory effects on durable fistula sealing (55.71% amylase reduction). A three-tier defense (maximal tumor elimination, sustained immune memory, and physical barrier) mitigates the risk of the prorepair phase. By integrating tumor elimination, immune remodeling, and tissue repair with clear temporal logic, CMCGel offers a promising strategy for the comprehensive perioperative management of pancreatic cancer.

## Supplementary Information

Below is the link to the electronic supplementary material.

Supplementary file1 (DOCX 12793 KB)
