# Supplementary material for: A Manganese-Chlorella Hydrogel for an Integrated “Remove–Remodel–Repair” Strategy in Pancreatic Cancer Therapy
Source: Nanomicro Lett. 2026 Sep 9;19:45. doi: 10.1007/s40820-026-02344-z (PMC13554046; doi:10.1007/s40820-026-02344-z)
Supplement: Supplementary file 1 [file 40820_2026_2344_MOESM1_ESM.docx]

Supporting Information for

**A Manganese-Chlorella Hydrogel for an Integrated “Remove-Remodel-Repair” Strategy in Pancreatic Cancer Therapy**

Tinglin Zhang^1,^ ^4, #^, Zhu Peng^1, 3, #^, Shijin Zhao^2, #^, Xin Jin^6, #^, Yiqi Du^1, 2, 4, 5,^ *, Zhaoshen Li^1, 2, 4, 5,^ *, Kaixuan Wang^2,^ *, Jie Gao^1, 2, 3, 4,^ *

^1^ Changhai Clinical Research Unit, Shanghai Changhai Hospital, Naval Medical University, Shanghai 200433, P. R. China

^2^ Department of Gastroenterology, Shanghai Changhai Hospital, Naval Medical University, Shanghai 200433, P. R. China

^3^ College of Life Science, Mudanjiang Medical University, Mudanjiang 157011, P. R. China

^4^ Shanghai Key Laboratory of Nautical Medicine and Translation of Drugs and Medical Devices, Shanghai 200433, P. R. China

^5^ National Key Laboratory of Immunity and Inflammation, Naval Medical University, Shanghai 200433, P. R. China

^6^ Department of Hepatic Surgery, Fudan University Shanghai Cancer Center, Shanghai Medical College, Fudan University, Shanghai 200032, P. R. China

^#^Tinglin Zhang, Zhu Peng, Shijin Zhao, and Xin Jin contributed equally to this work.

* Corresponding authors. E-mail: [gaojiehighclea@smmu.edu.cn](mailto:gaojiehighclea@smmu.edu.cn) (Jie Gao); [wangkaixuan224007@163.com](mailto:wangkaixuan224007@163.com) (Kaixuan Wang); [zhsl@vip.163.com](mailto:zhsl@vip.163.com) (Zhaoshen Li); [duyiqi@hotmail.com](mailto:duyiqi@hotmail.com) (Yiqi Du)

**S1** **Supplementary Text**

**S1.1 Materials**

***S1.1.1 Reagents***

Carboxymethyl chitosan (CMCS, degree of substitution ≥ 80%, catalog number 83512-85-0), calcium chloride (catalog number 1305-78-8), and polyethylene glycol (PEG 200, catalog number 25322-68-3) were obtained from Macklin (Shanghai, China). Manganese chloride tetrahydrate (MnCl_2_·4H_2_O; catalog number 13446-34-9), alginate (ALG; catalog number 9005-38-3), and carboxymethyl cellulose (CMC; catalog number 9004-32-4) were obtained from Aladdin (Shanghai, China). Anhydrous ferric chloride (catalog number 7705-08-0) and ferrous sulfate (batch number 7782-63-0) were acquired from the National Pharmacaceutical Group Chemical Reagents Co., Ltd. (Hu Shi, China). Chlorella extract powder was obtained from Shanxi Zhenghe Pharmaceutical Co., Ltd. (Shanxi, China). Artificial pancreatic fluid (APF; catalog number XF70001) was acquired from Nanjing XinFan Biotechnology Co., Ltd. (Nanjing, China). Mannitol (catalog number M9647, CAS number 69-65-8) was acquired from Merck KGaA (Darmstadt, Germany). Reagents related to cell culture, including Dulbecco’s modified Eagle’s medium (DMEM, catalog number 11965092), were purchased from Gibco (Thermo Fisher Scientific, USA). Blocking serum derived from sheep (catalog number ZLI-9022) was sourced from Beijing Zhongshan Golden Bridge Biotechnology Co., Ltd. (Beijing, China). A cell counting kit-8 (CCK-8, catalog number C0042) and a Bradford protein assay kit (catalog number P0006) were obtained from Beyotime Biotechnology (Shanghai, China). A high-mobility group box 1 (HMGB-1) ELISA kit (catalog number U96-3601E) was obtained from YOBIBIO (Shanghai, China). Additionally, a mouse interferon beta (IFN-β) ELISA kit (catalog number EM1148) and a mouse tumor necrosis factor alpha (TNF-α) ELISA detection kit (catalog number EM0183) were acquired from FineTest (Wuhan, China). E7820 (catalog number 289483-69-8), PF-573228 (catalog number 869288-64-2), and FH535 (catalog number 108409-83-2) were acquired from MedChemExpress (Shanghai, China). Pirfenidone (catalog number 53179-13-8) and SN-011 (catalog number 2249435-90-1) were acquired from Targetmol (Shanghai, China).

***S1.1.2 Cells***

Mouse pancreatic cancer cells (Panc02, CL-0736) and human umbilical vein endothelial cells (HUVECs, CP-H082) were obtained from Procell (Wuhan, China). The cells were cultured in DMEM supplemented with 10% fetal bovine serum (FBS) and 1% penicillin‒streptomycin. The plants were cultivated in an incubator maintained at 37 °C with 5% carbon dioxide (CO_2_). Upon reaching 80–90% confluence, the cells were passaged approximately every 2–3 days. For the passaging process, a 0.25% trypsin-EDTA solution was used to enzymatically dissociate the cells at 37 °C for approximately 1‒3 min. The digestion was halted when the cells appeared rounded, and the intercellular gaps were visible under a microscope when complete medium supplemented with 10% FBS was introduced. The resulting cell suspension was collected through gentle pipetting and subsequently replated at an appropriate dilution. Additionally, rat pancreatic stellate cells (RPSTCs; SNP-R035) and complete culture medium for RPSTCs (SNPM-R035) were obtained from Sunncell (Wuhan, China).

***S1.1.3 Animals***

Male C57BL/6J mice and Sprague–Dawley (SD) rats, aged 6–8 weeks, were procured from specific pathogen-free (SPF) Biotechnology Co., Ltd. (Suzhou, China). The experimental protocols involving animals received approval from the animal ethics committee of Shanghai Changhai Hospital (Shanghai, China) (Approval No. CHEC (A.E.) 2025--049) and adhered strictly to the institution's guidelines for animal care and use. The animals were maintained in an SPF barrier environment under carefully regulated conditions: a room temperature of 22 ± 2 °C, a relative humidity of 50 ± 10%, and a 12-hour light/dark cycle. The rats were provided unrestricted access to standard laboratory rodent chow and sterilized drinking water. For the surgical and euthanasia procedures, anesthesia was induced *via* inhalation of anesthetic isoflurane. Induction was achieved with a mixture of 3–4% isoflurane (carrier gas: pure oxygen; flow rate, 1.0 L/min), with the concentration subsequently reduced to 1.5–2% to maintain surgical anesthesia. At the conclusion of the experiment, the mice were euthanized by the administration of gradually increasing concentrations of CO_2_. The method involved placing the animals in a euthanasia chamber where CO_2_ was introduced at a rate equivalent to 30% of the chamber’s volume per minute. The CO_2_ concentration was then gradually increased to more than 70% over a period of five min until respiration ceased. Death was subsequently confirmed through cervical dislocation.

**S1.2 Preparation and characterization of hydrogels**

***S1.2.1*** ***Preparation of chlorella extract***

The chlorella extract (CE) solution was formulated by dissolving broken-chlorella powder in a PEG solvent. Specifically, 100 mg of broken chlorella powder was introduced into 100 mL of PEG solution and subjected to continuous stirring at 100 revolutions per minute (rpm) for 4 h at a constant temperature of 80 °C. The mixture was subsequently centrifuged at 10,000 rpm for 10 min, after which the supernatant was collected. To ensure the uniformity, sterility, and storage stability of the solution, it was sterilized *via* a 0.22 μm syringe filter.

***S1.2.2 Characterization of chlorella extract***

The UV‒Vis absorption spectrum of the CE was recorded in the range of 350–750 nm *via* a UV‒Vis spectrophotometer (Shimadzu SSL UV-2600i, Japan) to evaluate its composition and stability. The chlorophyll content in the CE was quantitatively analyzed *via* a chlorophyll a standard (C805046; Macklin, Shanghai, China) as a reference.

***S1.2.3 Preparation of hydrogels***

(1) Preparation of metal ion‒polysaccharide cross-linked hydrogels: Initially, ALG, CMC, and CMCS powders were individually dissolved in ultrapure water to formulate anionic polysaccharide solutions at a concentration of 75 mg/mL. In parallel, solutions of FeCl_3_, MgSO_4_, ZnSO_4_, CuSO_4_, and MnCl_2_ were prepared by dissolving each compound separately in ultrapure water to achieve a concentration of 50 mM for each metal ion. Afterward, 500 μL of each anionic polysaccharide solution was thoroughly mixed with 500 μL of each metal ion solution, yielding a total of 15 distinct experimental groups. Following a 30-min incubation period, the gelation properties of the mixtures were assessed using the inclined bottle method. Notably, all the reagents were sterilized by ultraviolet irradiation after weighing, and solution preparation and mixing operations were performed within a laminar flow hood.

(2) Prescription Optimization of Hydrogels: Based on the two criteria of adhesive strength (the test method is detailed in section S1.2.6) and mass retention rate in a trypsin environment (the test method is detailed in section S1.2.7), the hydrogel synthesized by crosslinking CMCS with Mn^2+^ (designated CMGel) was selected from the aforementioned 15 groups of hydrogels for subsequent experiments. Furthermore, using gel uniformity (Fig. S2A-B) and biocompatibility with human umbilical vein endothelial cells (HUVECs; Fig. S2C-E) as the basis for evaluation, the prescriptions for CMGel and CMCGel hydrogels were ultimately determined.

(3) Preparation of CMGel: First, CMCS powder was dissolved in ultrapure water to prepare a 75 mg/mL CMCS solution. Moreover, MnCl_2_ was dissolved in ultrapure water to prepare a 50 mM manganese ion (Mn^2+^) solution. Subsequently, 600 μL of CMCS solution, 500 μL of Mn^2+^ solution, and 200 μL of ultrapure water were mixed uniformly and allowed to stand for 30 min to crosslink and form CMGel hydrogels. The sterilization procedure was the same as that described in Section S1.2.3 (1). The final concentrations of each component in CMGel were determined to be 34.6 mg/mL CMCS and 26.9 mM Mn^2+^.

(4) Preparation of CMCGel: The procedure was the same as above, except that 200 µL of a 487.5 µg/mL CE solution was used instead of ultrapure water. After crosslinking, the CMGel hydrogel loaded with CE was obtained. The final concentrations of each component in CMCGel were 34.6 mg/mL CMCS, 26.9 mM Mn^2+^, and 75.0 μg/mL CE.

***S1.2.4 Morphology and structural analysis of the hydrogels***

The macroscopic morphology and color variations between the two hydrogels, CMGel and CMCGel, were assessed *via* the inclined bottle test. The cross-sectional morphology of the hydrogels after freeze-drying was examined by scanning electron microscopy (SEM; ZEISS GeminiSEM 300; Oberkochen, Germany) to elucidate their microscopic structures. Quantitative structural analysis of the SEM images was performed *via* ImageJ software (NIH, National Institutes of Health, USA) to determine the average pore diameter, pore distribution, and porosity of the hydrogels. The porosity (%) was calculated by measuring the proportion of black pixels, which is indicative of pore areas, relative to the total pixel count in the binary images:

$Porosity (\%)=\frac{Number of Black Pixels}{Total Number of Pixels}\times100\%$	(S1)

CMCGel was stored at temperatures of 4 °C, 25 °C, and 37 °C for a period of 28 days, during which time changes in its dry weight were monitored to assess the stability and structural integrity of its solid content.

***S1.2.5 Analysis of the crosslinking mechanism of the hydrogels***

The elemental composition and distribution of the two hydrogels, CMGel and CMCGel, were examined *via* SEM (ZEISS GeminiSEM 300, Oberkochen, Germany) in conjunction with energy-dispersive spectrometry (EDS).

The chemical state and coordination environment of the elements within CMGel were investigated through X-ray photoelectron spectroscopy (XPS; Thermo Scientific K-Alpha, Massachusetts, USA). Prior to analysis, the freeze-dried CMGel was affixed to the sample stage *via* a conductive adhesive, and an Al Kα X-ray source was employed at an energy setting of 50 eV.

Fourier transform infrared spectroscopy (FTIR, Thermo NEXUS 670 M, Massachusetts, USA) was used to analyze the functional groups and chemical bonds present in the CMCSs and CMGels. The CMCS powder and the freeze-dried CMGel were each mixed with potassium bromide and ground separately before being pressed into samples, with scanning wavenumbers ranging from 600–4000 cm^-1^.

The zeta potential of the CMCS and the two hydrogels was assessed *via* a zeta potential analyzer (Malvern Zetasizer Nano ZS90, Malvern, UK). For the CMCS powder, a 1 mg/mL solution was prepared by dissolving it in ultrapure water. In contrast, the two hydrogels were freeze-dried, ground, and homogenized to produce micron-sized particles, which were subsequently dispersed in ultrapure water to create 1 mg/mL suspensions for measurement.

***S1.2.6 Analysis of the injection, self-healing and adhesion properties of the hydrogels***

The injectability of the hydrogel was quantitatively assessed *via* a rheometer (NETZSCH, Kinexus Ultra+, Selb, Germany) and qualitatively verified *via* a syringe extrusion experiment. The rheological analysis was performed in frequency scanning mode at a constant angular frequency of 1 rad/s to determine the material’s linear viscoelastic response under dynamic conditions, thereby facilitating predictions of its injectability. For qualitative verification, approximately 1.0 mL of hydrogel was introduced into a syringe equipped with a needle with a 0.5 mm inner diameter and extruded at a steady rate of 5 mm/min. The continuity and stability of the extrusion process were monitored to thoroughly evaluate the hydrogel’s injectability and plasticity potential.

Additionally, the self-healing properties of the hydrogels were quantitatively examined *via* the same rheometer, complemented by a qualitative cutting-staining-recombination-healing-pulling experiment. During the rheological test for self-healing, a cylindrical hydrogel sample with a diameter of 25 mm was positioned between two parallel plates with a 0.5 mm gap. Continuous scanning was conducted at a frequency of 1 Hz and a strain of 1% for a duration of 300 s to measure the storage modulus (G’) and loss modulus (G”), thereby confirming the material’s solid-state characteristics. An alternating strain cycle, consisting of 1% strain for 60 s followed by 100% strain for another 60 s, was subsequently implemented to observe variations in G’ and G”, thereby evaluating the self-healing capacity. In the qualitative experiment, the CMGel was bisected, with one half stained with Coomassie Brilliant Blue, and then rejoined with the unstained half. Observations were made regarding color diffusion and the integrity of the hydrogel upon mechanical separation. For CMCGel, Rhodamine B served as the staining agent, with all the other procedures remaining consistent.

The adhesive properties of the hydrogel were quantitatively assessed *via* a mechanical testing apparatus (HAlDA International, HD-B609C-S, Suzhou, China) and qualitatively validated *via* adhesion experiments involving mouse tissues, including the spleen, pancreas, skin, and muscle. First, the adhesion strength of the hydrogel was quantitatively evaluated under wet tissue conditions mimicking the pancreatic fluid microenvironment. Specifically, scratches were created on the surface of porcine skin (20 × 10 × 2 mm) to simulate tissue wounds, and the hydrogel (20 × 10 × 2 mm) was uniformly applied to the wound area. The samples were then placed in artificial pancreatic fluid (APF, pH 8.0, containing trypsin, lipase, amylase, and a bicarbonate buffer system) and continuously shaken at 100 rpm at a constant temperature of 37 °C to simulate the physiological environment *in vivo*. Samples were collected at 10 minutes, 24 hours, and 48 hours and then fixed onto the surface of two overlapping plates for quantitative testing of adhesion strength. An overlapping shear test was performed at a rate of 10 mm/min, and the peak adhesive strength at the point of separation was documented. For intuitive verification, the hydrogel was applied between tweezers and tissues, with the tissues positioned beneath. The adhesive efficacy of the hydrogel on four distinct tissue types was observed under the influence of gravitational forces.

***S1.2.7 Analysis of the swelling and degradation behavior of the hydrogels***

To investigate the swelling and degradation properties of hydrogels in various physiological microenvironments, in this study, a two-layer experimental apparatus consisting of a Transwell chamber and a Petri dish with the hydrogel placed in the upper layer was developed. To simulate different physiological conditions, the pH of phosphate-buffered saline (PBS) was adjusted to three different values: 6.5 to mimic the tumor microenvironment, 7.4 to represent the normal physiological environment, and 8.0 to mimic the pancreatic fistula microenvironment. Additionally, APF (pH 8.0) conditions were established. All experimental procedures were conducted under constant temperature regulation (37 °C) and orbital agitation (100 rpm) to maintain the hydrogels in a dynamic fluid environment, thereby more accurately recapitulating the physiological conditions of interstitial fluid *in vivo*.

The swelling behavior of the hydrogel was evaluated by measuring its wet weight at various time intervals. Prior to each measurement, the excess surface liquid was carefully removed with lint-free paper. The swelling ratio was determined *via* the following formula:

$Swelling ratio (\%)=\left( \frac{W_{t}-W_{0}}{W_{0}} \right)\times100\%$	(S2)

where w_t_ denotes the weight of the hydrogel after PBS absorption at a given time point and w_0_ represents the initial weight of the hydrogel.

The degradation behavior of the hydrogels was evaluated by determining their dry weight at various time intervals. Prior to each measurement, the samples were freeze-dried and subsequently weighed. The degradation rate was calculated *via* the following formula:

$Degradation rate (\%)=\left( \frac{W_{0}-W_{t}}{W_{0}} \right)\times100\%$	(S3)

where w_0_ represents the initial mass of the hydrogel and w_t_ denotes the mass at a given time point.

The mass retention rate of the hydrogels was also evaluated by determining their dry weight at various time intervals. Prior to each measurement, the samples were freeze-dried and subsequently weighed. The mass retention rate was calculated *via* the following formula:

$Mass retention rate (\%)=\left( \frac{W_{t}}{W_{0}} \right)\times100\%$	(S4)

where w_0_ represents the initial mass of the hydrogel and w_t_ denotes the mass at a given time point.

***S1.2.8 Analysis of the release behavior of Mn******^2+^ and CE from hydrogels***

To investigate the release kinetics of Mn^2+^ and CE from the hydrogel, the same monitoring apparatus and experimental conditions as those described in section S1.2.7 were employed.

The release rate was determined by quantifying the Mn^2+^ and CE contents in the lower PBS solution. The absorbance of Mn^2+^ was measured at a wavelength of 450 nm *via* a Mn^2+^ detection kit in conjunction with a UV‒Vis spectrophotometer (Shimadzu, SSL UV-2600i, Japan). A standard curve correlating the absorbance with the concentration was constructed. The absorbance of the lower PBS at 450 nm was subsequently recorded at various time intervals, and the Mn^2+^ concentration was derived from the standard curve. The release rate of Mn^2+^ was calculated *via* the following formula:

$Mn release rate (\%)=\left( \frac{C_{t}V_{t}}{C_{0}V_{0}} \right)\times100\%$	(S5)

where c_t_ denotes the concentration of Mn^2+^ in the PBS solution, v_t_ represents the total volume of PBS, c_0_ represents the initial concentration of Mn^2+^ in CMCGel, and v_0_ represents the volume of CMCGel.

A standard curve of absorbance versus concentration for CE was constructed at a wavelength of 667 nm *via* a UV‒Vis spectrophotometer (Shimadzu, SSL UV-2600i, Kyoto, Japan). The absorbance of PBS at 667 nm was measured at various time intervals, and the concentration of CE was determined on the basis of the established standard curve. The release rate of CE was subsequently calculated *via* the following formula:

$CE release rate (\%)=\left( \frac{C_{t}V_{t}}{C_{0}V_{0}} \right)\times100\%$	(S6)

where c_t_ denotes the concentration of CE in PBS, v_t_ represents the total volume of PBS, c_0_ represents the initial concentration of CE in CMCGel, and v_0_ represents the volume of CMCGel.

***S1.******2.9 Analysis of the performance of the hydrogel in generating reactive oxygen species through photodynamic therapy and chemodynamic therapy***

To assess the ability of CMCGel to produce reactive oxygen species (ROS) *via* photodynamic therapy (PDT) and chemodynamic therapy (CDT), this study utilized four distinct ROS probes: SOSG and DPBF for singlet oxygen (^1^O_2_), DHE for superoxide anion (O_2_·⁻), and MB for hydroxyl radical (·OH). The hydrogel was coincubated with each probe and subsequently exposed to interventions under rigorously controlled light conditions (power density ≤ 0.5 W/cm^2^, duration ≤ 5 min, within established safety thresholds). The potential and specific types of ROS generated by CMCGel through PDT and CDT mechanisms were then analyzed by quantifying changes in the fluorescence intensity or absorbance of the resultant solutions. Notably, for the detection of ·OH *via* MB, experiments were conducted at various pH values (6.5 and 7.4), with the addition of H_2_O_2_ as a reaction substrate to facilitate the evaluation of the CDT process.

**S1.3 Biocompatibility testing**

***S1.3.1 Cell safety assays***

HUVECs were cultured at a density of 3,000 cells per well in a 96-well plate, with each well containing 100 μL of DMEM for an overnight incubation period. Following this initial culture, the cells were subjected to a 48-hour treatment across various experimental groups: Control, Control + laser, CMGel, CMCGel, and CMCGel + laser (with concentrations of CE: 75.0 μg/mL; Mn^2+^: 26.9 mM; CMCS: 34.6 mg/mL). After irradiation with a 660 nm laser, the medium was replaced with medium containing CCK-8. The cells were subsequently incubated for 1 h, after which the absorbance at 450 nm was quantified *via* a microplate reader (Varioskan Flash, Thermo, Massachusetts, USA).

***S1.3.2 Hemolysis test***

The blood sample was initially combined with PBS and subjected to centrifugation at 1500 rpm for 15 min to facilitate the removal of hemoglobin. The erythrocytes were subsequently resuspended in PBS to achieve a final concentration of 10%. These cells were subsequently allocated to groups and treated with equal volumes of control, control + laser, CMGel, CMCGel, and CMCGel + laser (with concentrations of CE: 75.0 μg/mL; Mn^2+^: 26.9 mM; CMCS: 34.6 mg/mL). The mixture was then incubated at 37 °C for 1 hour. The samples were subsequently subjected to a second centrifugation at 1000 rpm for 5 min. The hemolysis rate in the supernatant was quantitatively assessed by measuring the absorbance at 545 nm *via* a microplate reader (Varioskan Flash, Thermo, Massachusetts, USA).

***S1.3.3 In vivo biodistribution experiment***

To examine the spatiotemporal distribution characteristics of CMCGel *in vivo*, experimental mice were euthanized at predetermined intervals following the administration of 100 μL of the hydrogel. The *in vivo* degradation rate of the hydrogel was determined as outlined in Section **S1.2.7**. Blood samples and major organs, including the liver, spleen, and kidneys, were subsequently collected, accurately weighed, and subjected to digestion in ultrapure water at low temperature for 72 h. Following secondary dilution, the Mn content in the digested samples was analyzed *via* inductively coupled plasma‒optical emission spectrometry (ICP‒OES; Optima 8300, PerkinElmer, Massachusetts, USA) to infer the distribution levels of the hydrogel within the respective tissues.

***S1.3.4 In vivo biocompatibility testing***

The biocompatibility of the therapeutic materials was evaluated through monitoring murine body weight and performing hematoxylin and eosin (H&E) staining of sections of principal organs. Fourteen days after treatment, the mice that were administered various hydrogels were euthanized as previously described, and the primary organs, including the heart, liver, spleen, lungs, and kidneys, were harvested. These organs were then fixed in 4% paraformaldehyde, embedded in paraffin, and sectioned for H&E staining following established protocols.

**S1.4 In vitro antitumor assays**

***S1.4.1 Cytotoxicity assays***

Panc02 cells were seeded into a 96-well plate at a density of 5 × 10^3^ cells per well, with 100 μL of complete DMEM added to each well. Following an overnight incubation, the cells were subjected to a 24-hour treatment across the following experimental groups: Control, Control with laser, CE, CE with laser, CMGel, CMCGel, and CMCGel with laser. The concentrations used were as follows: 75.0 μg/mL CE, 26.9 mM Mn^2+^, and 34.6 mg/mL CMCS. The groups designated for light exposure were irradiated with a 660 nm laser at a power density of 0.5 W/cm^2^ for 5 min during the treatment period. The medium was subsequently replaced with fresh medium containing the CCK-8 reagent. After a 1-hour incubation, the absorbance of each well was measured at 450 nm *via* a microplate reader to evaluate cytotoxicity.

***S1.4.2 Apoptosis assays***

Panc02 cells were seeded in a 6-well plate at a density of 3 × 10^5^ cells per well, with 2 mL of complete DMEM added to each well. Following an overnight incubation, the medium was replaced with fresh medium containing the designated treatments: Control, Control + Laser, CE, CE + Laser, CMGel, CMCGel, and CMCGel + Laser. The concentrations used were CE at 75.0 μg/mL, Mn^2+^ at 26.9 mM, and CMCS at 34.6 mg/mL. The cells were then cultured for an additional 24 h. The groups designated for light exposure were subjected to 660 nm laser irradiation at a power density of 0.5 W/cm^2^ for 5 min during the treatment period. After treatment, the cells were harvested, subjected to trypsin digestion, centrifuged, washed with PBS, and resuspended in Annexin V binding buffer. The cells were subsequently incubated with Annexin V-FITC and PI in the dark at 4 °C for 15 min. Flow cytometry analysis (Beckman CytoFLEX S, California, USA) was conducted to quantify the proportions of apoptotic and necrotic cells.

***S1.4.3 Live/Dead cell staining***

Panc02 cells were seeded at a density of 5 × 10^4^ cells per well in the lower chamber of 24-well plate inserts, with each well receiving 500 μL of complete DMEM. Following an overnight incubation period to facilitate cell adhesion, the cells were subjected to treatments, including the control, control + laser, CE, CE + laser, CMGel, CMCGel, and CMCGel + laser treatments (where CE: 75.0 μg/mL; Mn^2+^: 26.9 mM; CMCS: 34.6 mg/mL), for a duration of 24 h. The cells in the groups designated for light treatment were exposed to 660 nm laser irradiation at an intensity of 0.5 W/cm^2^ for 5 min. The cells were subsequently stained with calcein-AM/PI double-staining reagent for 30 min, followed by washing with PBS. Imaging was performed *via* an inverted fluorescence microscope (Olympus BX51TRF, Tokyo, Japan) to differentiate live cells, indicated by green fluorescence, from dead cells, indicated by red fluorescence.

***S1.4.4 ROS level determination***

To assess the effects of various treatments on intracellular ROS levels in Panc02 cells, a DCFH-DA probe was used in accordance with the manufacturer's guidelines. Panc02 cells were seeded at a density of 5 × 10^4^ cells per well in the lower chamber of 24-well plate inserts, with each well receiving 500 μL of complete DMEM. Following an overnight incubation, the medium was replaced with fresh medium containing the designated treatments (control, control + laser, CE, CE + laser, CMGel, CMCGel, CMCGel + laser; CE: 75.0 μg/mL; Mn^2+^: 26.9 mM; CMCS: 34.6 mg/mL), and the cells were cultured for an additional 24 h. The groups designated for light exposure were subjected to 660 nm laser irradiation (0.5 W/cm^2^, 5 min) during the treatment period. The cells were subsequently incubated with a 10 μmol/L DCFH-DA probe (dissolved in 1 mL of serum-free medium) in the dark for 30 min, followed by washing with PBS. The cells were then imaged *via* an inverted fluorescence microscope (Olympus BX51TRF, Tokyo, Japan) for subsequent analysis.

***S1.4.5 Immunogenic cell death experiments***

Panc02 cells were seeded at a density of 5 × 10^4^ cells per well in the lower chamber of 12-well plate inserts, with 1 mL of complete DMEM added to each well. Following an overnight incubation, the medium was replaced with fresh medium containing various treatments: control, control + laser, CE, CE + laser, CMGel, CMCGel, and CMCGel + laser (CE: 75.0 μg/mL; Mn^2+^: 26.9 mM; CMCS: 34.6 mg/mL). The cells were then cultured for an additional 24 hours. The groups designated for light exposure were subjected to 660 nm laser irradiation (0.5 W/cm^2^, 5 min) during the treatment period. The cells were subsequently incubated with an anti-CRT primary antibody overnight at 4 °C, followed by washing with PBS and incubation with a Cy3-labeled secondary antibody for 60 min. Antibody dilutions are detailed in Table S2. Concurrently, cell supernatants were collected, and HMGB-1 concentrations were measured using ELISA kits. Moreover, the cells were lysed, and ATP levels were assessed with a bioluminescence-based assay kit.

***S1.4.6*** ***cGAS-STING signaling pathway activation assays***

Panc02 cells were seeded at a density of 5 × 10^4^ cells/well in the lower chamber of 12-well Transwell plates, with 1 mL of complete DMEM added per well. After overnight incubation, the medium was replaced with fresh medium containing different treatments: control, CMCS-CE, and CMCGel (CE: 75.0 μg/mL; Mn^2+^: 26.9 mM; CMCS: 34.6 mg/mL). The samples were subsequently prepared according to the instructions provided with the Mn^2+^ detection kit. Instrument detection details are described in section S1.2.8. The Mn^2+^ concentration in the cytoplasm was calculated on the basis of its UV‒Vis absorption‒concentration standard curve.

To measure the level of double-stranded DNA (dsDNA) in the cytoplasm, the same cell culture protocol as described above was used. The experimental treatment groups were as follows: control, CMCS + CE + laser, CMCGel, CMCGel + mannitol (·OH scavenger), and CMCGel + laser (CE: 75.0 μg/mL; Mn^2+^: 26.9 mM; CMCS: 34.6 mg/mL; 660 nm laser: 0.5 W/cm^2^, 5 min). The cytoplasmic dsDNA level was subsequently determined using the PicoGreen dsDNA quantification method. Specifically, cells were treated with digitonin to disrupt the plasma membrane while preserving the nuclear membrane, thereby releasing the cytoplasmic contents. After centrifugation, the supernatant was collected to remove nuclear debris. The supernatant was then mixed with PicoGreen working solution at a 1:1 ratio and incubated for 5 minutes at room temperature in the dark. Finally, fluorescence was measured using a microplate reader (excitation at 485 nm, emission at 528 nm), and the total amount of cytoplasmic dsDNA was calculated.

To detect cGAS-STING pathway activation-related protein expression by WB, the same cell culture protocol as described above was used. Notably, the experimental treatment groups were as follows: control, CMCS + CE + laser, CMCGel + laser, and CMCGel + laser + SN-011 (a cGAS-STING pathway inhibitor); CE: 75.0 μg/mL; Mn^2+^: 26.9 mM; CMCS: 34.6 mg/mL; 660 nm laser: 0.5 W/cm^2^, 5 min. The cells were subsequently lysed with RIPA lysis buffer, and protein concentrations were determined using a BCA kit. The proteins were separated by sodium dodecyl sulfate‒polyacrylamide gel electrophoresis, transferred to membranes, and incubated overnight at 4 °C with primary antibodies against STING, p-STING, TBK1, p-TBK1, IRF3, p-IRF3, and β-Actin. Antibody dilutions are detailed in Table S2. After the membranes were incubated with horseradish peroxidase-conjugated secondary antibodies for 1 h at room temperature, they were imaged using a chemiluminescence imaging system (GE Healthcare, ImageQuant LAS 4000, USA).

***S1.4.7 Protein adsorption and antigen capture assays***

One milliliter of CMGel or CMCGel hydrogel was accurately dispensed into separate 5 mL centrifuge tubes. Three milliliters of a solution containing 5% fetal bovine serum (FBS) was subsequently added to each tube to facilitate protein absorption, with the FBS solution serving as the baseline control (m_c_). All the samples were incubated in a constant temperature shaker set at 37 °C with agitation at 100 rpm for a duration of 6 h. Following incubation, the samples were centrifuged at 10,000 rpm for 3 min to separate the hydrogels from the unadsorbed proteins. The protein concentrations in the supernatants of both the treated group (m_t_) and the control group (m_c_) were quantified *via* the Bradford protein assay. The protein adsorption rate was determined *via* the following formula:

$Antigen absorption rate (\%)=\left( \frac{m_{c}{- m}_{t}}{m_{c}} \right)\times100\%$	(S7)

***S1.4.8 Dendritic cell activation assays***

To generate bone marrow-derived dendritic cells (BMDCs), culture medium comprising 500 mL of RPMI-1640, 50 mL of FBS, 5 mL of penicillin‒streptomycin, and 2 μL of β-mercaptoethanol was used. Following the euthanasia of C57 mice, the femurs and tibias were aseptically excised, and the bone marrow cavities were flushed with PBS. The cells were subsequently collected *via* centrifugation at 1500 rpm for 5 min. The resulting cell pellet was resuspended in 3 mL of complete DMEM and seeded into a culture dish containing 10 mL of medium supplemented with 20 ng/mL granulocyte‒macrophage colony‒stimulating factor (GM‒CSF). The cells were incubated at 37 °C with 5% CO_2_, and the medium was changed every 48 h. To assess dendritic cell maturation, BMDCs were seeded in the lower chamber of a transwell system (Corning 3422, pore size 8 μm) and exposed to the supernatants of Panc02 cells treated with various conditions: control, control + laser, CE, CE + laser, CMGel, CMCGel, and CMCGel + laser (where CE: 75.0 μg/mL; Mn^2+^: 26.9 mM; CMCS: 34.6 mg/mL). Following a 24-h coculture period, the groups designated for light exposure were subjected to laser irradiation (0.5 W/cm^2^ for 5 min) and subsequently cultured for an additional 24 h. The BMDCs were subsequently harvested for flow cytometric analysis to determine the proportions of mature DCs (CD11c^+^ CD80^+^ and CD11c^+^ CD86^+^). Additionally, the concentrations of TNF-α and IL-6 in the supernatants were quantified *via* ELISA.

**S1.5 In vivo antitumor assays**

***S1.5.1 Establishment and treatment of simultaneous primary and distal tumor-bearing mouse models***

In this study, bilateral subcutaneous tumor models were established in C57BL/6J mice, with a primary tumor on the right side and a contralateral distant tumor on the left. First, 5×10^6^ Panc02 cells were injected subcutaneously into both flanks. Once the tumor volume reached approximately 100 mm^3^, the mice were randomized into five groups: control, control + laser, CMGel, CMCGel, and CMCGel + laser. For the CMGel and CMCGel groups, 100 μL of the corresponding hydrogel (75.0 μg/mL of CE, 26.9 mM of Mn^2+^, and 34.6 mg/mL of CMCS) was administered peritumorally around the right primary tumor every 7 days. Concurrently, the right primary tumor underwent local 660 nm laser irradiation at a power density of 0.5 W/cm^2^ for 5 minutes per session, which was delivered every 3 days. Notably, the left distant tumor received no intervention throughout the study, enabling systemic evaluation of the inhibitory effects of local treatment on both the primary and the untreated distant tumor.

***S1.5.2 Establishment and treatment of tumor rechallenge mouse models***

The mouse strain, tumor cell line, single-inoculation cell number, CMCGel formulation and single-injection volume, and laser treatment parameters used in this model were all consistent with those described in S1.5.1. The experiment comprised three groups, with the specific protocols as follows:

Group 1 (Tumor Resection Control Group): On day 1, tumor cells were subcutaneously inoculated on the right side of the mouse to establish a primary tumor; on day 7, the right-side tumor volume reached approximately 100 mm^3^; on day 18, the right-side tumor was completely resected; on day 20, tumor cells were inoculated on the left side of the mouse to simulate tumor rechallenge; and on day 28, the mice were euthanized, and the left-side tumors were excised.

Group 2 (sham surgery control group): On the right side of the mouse, on day 18, sham surgery was performed on the right side (blank control); on day 20, tumor cells were inoculated on the left side of the mouse; on day 28, the mice were euthanized, and the left-side tumors were excised.

Group 3 (CMCGel combined with laser treatment group): On day 1, tumor cells were subcutaneously inoculated on the right side of the mouse to establish a primary tumor; on day 7, the right-side tumor volume reached approximately 100 mm^3^; CMCGel combined with laser treatment was administered from day 7 to day 17; on day 18, the right-side tumor was completely resected; on day 20, tumor cells were inoculated on the left side of the mouse to simulate tumor rechallenge; no treatment was administered from day 20 to day 28; and on day 28, the mice were euthanized, and the left-side tumors were excised.

***S1.5.3 Monitoring of tumor volume***

The body weights and tumor sizes of the mice were measured every 48 h to systematically evaluate the therapeutic response. The tumor volume was calculated according to the following formula:

$Tumor volume (\mathrm{mm}^{3})=0.5 \times a \times b^{2}$	(S8)

where a is the longest diameter of the tumor and b is the shortest diameter.

***S1.5.4 Immune response assays***

Fourteen days post-treatment, the mice were euthanized to facilitate a comprehensive evaluation of the *in vivo* antitumor immune response. The tumor tissues were harvested, and samples embedded in paraffin were selected for subsequent H&E staining, immunofluorescence staining (e.g., CRT, HMGB1, INF-β, and CD8), and immunohistochemical (e.g., Ki67) analysis. Antibody dilutions are detailed in Table S2. To further elucidate the immune cell composition, both primary and metastatic tumor tissues, along with tumor-draining lymph nodes (TDLNs), were collected. The TDLNs were mechanically dissociated using a 70 μm cell strainer for 3–5 min, followed by washing with precooled PBS and centrifugation at 1400 rpm for 5 min to isolate the cells. The supernatant was discarded, and specific antibody panels were used for staining: mature DCs were identified *via* antibodies against CD45, CD11c, CD80, and CD86, and cytotoxic T lymphocytes (CTLs) were distinguished *via* antibodies against CD45, CD3, and CD8. Tumor-infiltrating effector T lymphocytes (TIEs) were detected *via* an antibody panel specific for CD45, CD3, CD8, and IFN-γ. Regulatory T cells (Tregs) were characterized and analyzed through the application of antibodies against CD25 and Foxp3.

**S1.6 In vivo and in vitro repair assays**

***S1.6.1 Cell migration assays***

A scratch assay was used to evaluate cell migration ability. Specifically, HUVECs were seeded at a density of 5 × 10^5^ cells per well in a 6-well plate, with 2 mL of complete medium added to each well. After overnight culture to form a confluent monolayer, a uniform scratch with a width of approximately 0.5–1 mm was made in the cell layer *via* a 200 μL sterile pipette tip. The detached cells were removed by gently rinsing twice with PBS, after which the medium was replaced with low-serum DMEM containing 2% FBS. The cells in the control, CMGel, and CMCGel groups were treated. The scratch area was observed and imaged under an inverted microscope at 0, 6, 12, and 24 h postcratch to assess cell migration. In addition, RPSTC was cultured in dedicated complete culture medium (SNPM-R035), and the rest of the experimental procedures were the same as those described above. The cell migration images were analyzed *via* ImageJ software, and the healing rate was calculated *via* the following formula:

$\mathrm{Healing} rate (\%)=\left( \frac{initial trace area - final trace area}{initial trace area} \right)\times100\%$	(S9)

***S1.6.2 Establishment and treatment of the pancreatic fistula rat model***

A rat model of pancreatic fistula was developed to systematically assess the reparative effects of CMCGel. Male Sprague–Dawley rats, aged 6–8 weeks, were procured from SPF Biotechnology Co., Ltd. (Suzhou, China). The experimental design comprised five distinct groups: the control group (undergoing only laparotomy), the surgery group (subjected to pancreatic duct resection without subsequent treatment), the surgery + fibrin sealant group (undergoing pancreatic duct resection followed by fibrin sealant treatment, serving as the clinical standard control), the surgery + CMGel group (undergoing pancreatic duct resection followed by CMGel treatment), and the surgery + CMCGel group (undergoing pancreatic duct resection followed by CMCGel treatment, with concentrations of CE at 75.0 μg/mL, Mn^2+^ at 26.9 mM, and CMCS at 34.6 mg/mL). These groups were strategically designed to evaluate the effects of surgical trauma, the natural progression of pancreatic fistula, the efficacy of existing treatment materials, and the effects of CE.

Each group consisted of five randomly assigned rats. Prior to surgical intervention, the rats underwent a 24-hour fasting period and were anesthetized *via* intraperitoneal administration of tribromoethanol (5 mL). Following the induction of anesthesia, the rats were placed in the supine position, and a 3-cm longitudinal incision was made along the left upper abdominal anterior line. Methylene blue solution was applied locally to the incision site to delineate the surgical field. A distal pancreatectomy accompanied by a splenectomy was subsequently conducted; the pancreas was transected, and an appropriate hydrogel was applied to the pancreatic transection surface. During the procedure, the splenic vessels were routinely ligated to mitigate bleeding risk. The abdominal wall and skin incisions were then closed using interrupted sutures with 3–0 suture material. Postoperatively, the rats were immediately placed on an electric heating pad to maintain body temperature and were provided with a liquid diet to facilitate recovery.

***S1.6.3 In vivo promotion of pancreatic fistula repair***

During the establishment and treatment of the pancreatic fistula rat model, the body weights of the rats were monitored daily, and their survival status was systematically observed. Forty-eight hours post-surgery, the rats were euthanized *via* isoflurane inhalation anesthesia in conjunction with CO_2_ asphyxiation, and the remaining pancreatic tissues were harvested for subsequent analysis. Peritoneal lavage fluid, collected two days post-surgery, was utilized to quantify lipase and amylase levels *via* ELISA, enabling the assessment of inflammation associated with pancreatic fistula. The pancreatic stump tissues were decalcified with EDTA, followed by paraffin embedding and sectioning for H&E staining. This facilitated the observation of ductal structural integrity and degree of fibrosis, as well as IL-6 immunohistochemical staining, to identify inflammatory cell infiltration, thereby providing a comprehensive evaluation of the repair-promoting efficacy of the materials. Antibody dilutions are detailed in Table S2. Concurrently, heart, liver, lung, and kidney tissues were subjected to H&E staining to evaluate systemic toxicity risk in accordance with ISO 10993 standards.

***S1.6.4 RNA sequencing and data analysis***

The initial step involved subjecting the raw sequencing data to quality assessment and preprocessing. Fastp was used to eliminate low-quality bases and adapter sequences, thereby yielding high-quality clean data. HISAT2 was subsequently employed to align the sequencing reads to the reference genome, whereas SAMtools facilitated the processing of BAM files to calculate alignment efficiency and assess the distribution of reads across genomic regions. Gene expression quantification was conducted *via* HTSeq, which counts the number of reads uniquely aligned to each gene, resulting in a raw expression matrix (read count). This matrix was then normalized to the TPM values. Intersample correlation analysis and principal component analysis (PCA) were performed on the normalized expression matrix to assess the consistency of the biological replicates and to discern differences between groups. Differential expression analysis was conducted *via* DESeq2, which employs screening criteria of |log_2_(fold change)| > 1 and a false discovery rate (FDR) of less than 0.05 to identify differentially expressed genes. Subsequent analyses included the identification of differential alternative splicing events *via* CASH, the detection of fusion genes *via* STAR-Fusion, and the identification of SNP/InDel variants through SAMtools mpileup. The outcomes were functionally annotated to dissect transcriptomic features across multiple dimensions. All the analytical workflows were executed within the R or Python programming environments.

***S1.6.5 Validation of the pancreatic fistula repair mechanism***

Passage 2 RPSTCs were used to validate the mechanism of pancreatic fistula repair. Cells were treated with control, CMCGel, and CMCGel + the following inhibitors: E7820 for fibronectin (FN) and integrin-β1, PF-573228 for FAK kinase activity, pirfenidone for TGF-β1, and FH535 for PPARγ. Protein expression was subsequently analyzed by WB. Cells were lysed in RIPA buffer, and protein concentrations were determined using a BCA assay. Proteins were separated by sodium dodecyl sulfate‒polyacrylamide gel electrophoresis (SDS‒PAGE) and transferred to membranes, which were then incubated overnight at 4 °C with primary antibodies against FN, integrin-β1, p-FAK, PPARγ, TGF-β1, and GAPDH. Antibody dilutions are detailed in Table S2. After the membranes were incubated with horseradish peroxidase-conjugated secondary antibodies at room temperature for 1 h, they were visualized using a chemiluminescence imaging system (GE Healthcare, ImageQuant LAS 4000, USA).

**S1.7 Statistical analysis**

Data visualization and statistical analyses were conducted *via* GraphPad Prism version 8.0.2 (GraphPad Software, San Diego, CA, USA). The results are expressed as the means ± standard deviations (SDs) and were evaluated *via* unpaired t tests or one-way ANOVA, followed by Tukey’s post hoc test, as appropriate. Statistical significance was determined at a threshold of *p* < 0.05. The data are denoted as follows: * *p* < 0.05; ** *p* < 0.01; *** *p* < 0.001; n.s., not significant (*p* > 0.05).

**S1.8 Synergy Analysis**

***S1.8.1 Valeriote formula for the synergy ratio***

The synergy ratio R was calculated as follows:

$R=\left( \frac{E_{\mathrm{observed}}}{E_{\mathrm{expected}}} \right)$	(S10)

where E_observed_ is the experimentally observed effect of the combination and E_expected_ is the expected additive effect, which is calculated as follows:

$E_{\mathrm{expected}}=\left( E_{A}+E_{B}-(E_{A} \times E_{B}) \right)$	(S11)

E_A_ and E_B_ represent the fractional effects (0–1) of individual treatments A and B, respectively. R > 1 indicates synergism, R = 1 indicates additivity, and R < 1 indicates antagonism.

For *in vivo* data where a separate PDT-alone group was not included, the *in vivo* PDT effect (E_PDT_) was estimated from the *in vivo* CDT effect (E_CDT_ = 0.2657) using the PDT/CDT potency ratio observed *in vitro* (65%/55% = 1.18): E_PDT_ = E_CDT_ × 1.18

***S1.8.2 ROS synergy index***

The ROS synergy index was calculated as the ratio of the observed ROS level to the expected additive ROS level:

$ROS synergy index=\left( \frac{\mathrm{ROS}_{\mathrm{observed}}}{\mathrm{ROS}_{\mathrm{expected}}} \right)$	(S12)

where ROS_expected_ = ROS_PDT_ + ROS_CDT_ − ROS_control_ (with ROS_control_ normalized to 1).

***S1.8.3 ICD synergy index***

The ICD synergy index for individual markers (e.g., CRT exposure) was calculated similarly:

$ICD synergy index=\left( \frac{\mathrm{Marke}_{\mathrm{observed}}}{\mathrm{Marke}_{\mathrm{expected}}} \right)$	(S13)

where Marke_expected_ = Marke_PDT_ + Marke_CDT_ − Marke_control_

***S1.8.4 Mechanistic and Comprehensive Synergy Evaluation***

To further substantiate the synergistic effect between PDT and CDT beyond the quantitative Valeriote formula, we evaluated synergistic amplification across multiple mechanistic levels, including ROS generation, ICD, and immune activation. Table S3 summarizes the synergy indices or fold-enhancements for key parameters.

To provide a holistic assessment, Table S4 integrates the evidence from direct/indirect calculations and multi-level mechanistic data to classify the strength of synergy.

**S2 Supporting Figures**

**Fig. S1** Correlation analysis between the occurrence of POPF in pancreatic cancer patients and their DFS and OS. The numbers within the hexagons refer to the corresponding reference numbers. The results revealed that the occurrence of POPF reduces the disease-free survival (DFS) and overall survival (OS) of patients, indicating that POPF is an unfavorable factor affecting patient prognosis. **p* < 0.05, ***p* < 0.01, ****p* < 0.001.

**Fig. S****2** Preparation and stability analysis of the CE. a) Schematic diagram of CE preparation. b) Photographs and c) UV‒vis absorption spectrum of the CE. The stability of the CE solution was characterized *via* d) photographs and e) absorbance at 667 nm. The data are presented as the means ± SDs (n = 3).

**Fig. S****3** Formulation optimization of CMCGel hydrogels. a, b) Optimization of the CMGel formulation (red horizontal lines indicate no gelation, red boxes indicate nonuniform gelation, green boxes indicate uniform gelation, “√” denotes successful gel formation, “×” denotes failure to form a gel, “uneven” indicates nonuniform gelation, and “even” indicates uniform gelation). c) Schematic illustration of the hydrogel treatment of HUVECs. The safety of HUVECs in d) CMGel (1 represents 34.6 mg/mL CMCS + 26.9 mM Mn^2+^, 2 represents 34.6 mg/mL CMCS + 30.8 mM Mn^2+^, 3 represents 46.2 mg/mL CMCS + 38.5 mM Mn^2+^) and e) CMCGel. The data are presented as the means ± SDs (n = 3); **p* < 0.05, ** *p* < 0.01, *** *p* < 0.001, **** *p* < 0.0001.

**Fig. S4** Structural analysis of the hydrogels. Pore size distribution diagram of a) CMGel and b) CMCGel. c) Quantitative diagram of the porosity of the hydrogels. The data are presented as the means ± SDs (n = 3); * *p* < 0.05, ** *p* < 0.01, *** *p* < 0.001, **** *p* < 0.0001.

**Fig. S5** Stability of the dry weights of the CMCGel hydrogels. The data are presented as the means ± SDs (n = 3).

**Fig. S****6** Analysis of the crosslinking mechanism of the hydrogels. a) Elemental mapping images and b) atomic percentages of CMGel and CMCGel (scale bars = 250 μm). c) XPS spectra of CMGel. d) Zeta potential of CMCS, CMGel, and CMCGel. The data are presented as the means ± SDs (n = 3).

**Fig. S7** Stability of hydrogels in different microenvironments. a) Storage modulus in different microenvironments and b) mass retention rate in the APF (pH 8.0) microenvironment of the two hydrogels. The data are presented as the means ± SDs (n = 3); * *p* < 0.05, ** *p* < 0.01, *** *p* < 0.001, **** *p* < 0.0001.

**Fig. S8** a) Mn^2+^ and b) CE concentration–absorbance standard curves.

**Fig. S****9** Safety of CMCGel + laser in HUVECs. a) Schematic illustration of the hydrogels + laser treatment of HUVECs. The safety of HUVECs under different treatments was assessed by b) a CCK-8 assay and c, d) live/dead cell staining (scale = 100 μm). The data are presented as the means ± SDs (n = 3); * *p* < 0.05, ** *p* < 0.01, *** *p* < 0.001, **** *p* < 0.0001.

**Fig. S10** *In vivo* degradation and distribution behavior of the hydrogels. a) *In vivo* degradation behavior of the hydrogels in C57BL/6J mice. b) The Mn contents in the blood, liver, and kidney were determined *via* ICP‒OES. The data are presented as the means ± SDs (n = 3); * *p* < 0.05, ** *p* < 0.01, *** *p* < 0.001, **** *p* < 0.0001.

**Fig. S1****1** *In vivo* biosafety assay*.* a) Photograph and b) quantitative analysis of the hemolysis of different hydrogels. c) Body weights and d) representative H&E-stained images of the major organs of C57BL/6J mice implanted with different hydrogels (scale = 100 μm). The data are presented as the means ± SDs (n = 3); * *p* < 0.05, ** *p* < 0.01, *** *p* < 0.001, **** *p* < 0.0001.

**Fig. S12** Quantitative analysis of apoptotic Panc02 cells after different treatments. The data are presented as the means ± SDs (n = 3); * *p* < 0.05, ** *p* < 0.01, *** *p* < 0.001, **** *p* < 0.0001.

**Fig. S1****3** a) Immunofluorescence (scale = 100 μm) and b) quantitative analysis of live and dead Panc02 cells after different treatments. The data are presented as the means ± SDs (n = 3); * *p* < 0.05, ** *p* < 0.01, *** *p* < 0.001, **** *p* < 0.0001.

**Fig. S14** a) Immunofluorescence (scale = 50 μm) and b) quantitative analysis of ROS in Panc02 cells after different treatments. The data are presented as the means ± SDs (n = 3); * *p* < 0.05, ** *p* < 0.01, *** *p* < 0.001, **** *p* < 0.0001.

**Fig. S15** a) Immunofluorescence (scale = 50 μm) and b) quantitative analysis of CRT in Panc02 cells after different treatments. The data are presented as the means ± SDs (n = 3); * *p* < 0.05, ** *p* < 0.01, *** *p* < 0.001, **** *p* < 0.0001.

**Fig. S16** a) Immunofluorescence (scale = 50 μm) and b) quantitative analysis of HMGB-1 in Panc02 cells after different treatments. The data are presented as the means ± SDs (n = 4); * *p* < 0.05, ** *p* < 0.01, *** *p* < 0.001, **** *p* < 0.0001.

**Fig. S17** a) Mn^2+^ concentration in the cytoplasm and b) dsDNA concentration of Pan02 cells after different treatments. The data are presented as the means ± SDs (n = 3); * *p* < 0.05, ** *p* < 0.01, *** *p* < 0.001, **** *p* < 0.0001.

**Fig. S18** STING, p-STING, TBK1, p-TBK1, IRF-3 and p-IRF3 levels (as measured by WB). 1 represents the control, 2 represents the CMCS +CE + laser, 3 represents the CMCGel + laser, and 4 represents the CMCGel + laser + SN-011 (cGAS-STING pathway inhibitor). The data are presented as the means ± SDs (n = 3).

**Fig. S19** DC activation assays. a) Schematic diagram of the DC maturation experiment. After the hydrogels were cocultured with Panc02 cells for 24 hours, the supernatant was collected and further cocultured with primary BMDCs for 24 hours. The BMDCs and the supernatant were subsequently collected separately. The proportion of CD11c^+^ CD80^+^ CD86^+^ cells was detected *via* flow cytometry, and the levels of IL-6 and TNF-α in the supernatant were measured *via* ELISA. b) Flow cytometry analysis of mature DCs (CD11c^+^ CD80^+^ CD86^+^ cells) *in vitro*. The data are presented as the means ± SDs (n = 3); * *p* < 0.05, ** *p* < 0.01, *** *p* < 0.001, **** *p* < 0.0001.

**Fig. S****20** Schematic diagram of the laser treatment of tumor-bearing mice. The primary tumor region (right side) of C57BL/6J mice was irradiated with a 660 nm laser (power density: 0.5 W/cm^2^; duration: 5 min).

**Fig. S21** Evaluation of the therapeutic effects on primary and distal pancreatic cancer in C57BL/6J-treated tumor-bearing mice after different treatments. a) Individual volume changes in primary and distal tumors. b) Representative images of tumor-bearing mice at the end of treatment (tumors indicated by yellow circles). Final volumes of c) primary and d) distal tumors. The data are presented as the means ± SDs (n = 5); * *p* < 0.05, ** *p* < 0.01, *** *p* < 0.001, **** *p* < 0.0001.

**Fig. S22** The Mn contents in the primary tumor were determined *via* ICP‒OES. The data are presented as the means ± SDs (n = 3); * *p* < 0.05, ** *p* < 0.01, *** *p* < 0.001, **** *p* < 0.0001.

**Fig. S23** Immunofluorescence (scale = 50 μm) of HMGB-1 in primary pancreatic cancer in C57BL/6J tumor-bearing mice after different treatments. The data are presented as the means ± SDs (n = 3).

**Fig. S24** a, b) Flow cytometry analysis of CTLs (CD45^+^ CD3^+^ CD8^+^) in primary and distant tumor tissues of mice following different treatments. The data are expressed as the means ± SDs (n = 3); **p* < 0.05, ***p* < 0.01, ****p* < 0.001, *****p* < 0.0001.

**Fig. S25** CMCGel + laser therapy inhibits tumor rechallenge. a) Schematic diagram of the establishment and treatment procedure of the tumor “treatment-resection-rechallenge” mouse model (C57BL/6J). Group 1 (the tumor resection control group): On day 1, tumor cells were inoculated on the right side; on day 7, the tumor volume reached approximately 100 mm^3^; on day 18, the right-sided tumor was completely resected; on day 20, tumor cells were inoculated on the left side; and on day 28, the tumor was sacrificed, and the left-sided tumor was excised. Group 2 (sham surgery control group): On day 1, PBS was injected on the right side; on day 18, sham surgery was performed; on day 20, tumor cells were inoculated on the left side; on day 28, the tumors were euthanized, and the left-sided tumors were excised. Group 3 (CMCGel + laser therapy group): On day 1, tumor cells were inoculated on the right side; on day 7, the tumor volume reached approximately 100 mm^3^; from day 7 to day 17, CMCGel combined with laser therapy was administered; on day 18, the right-side tumor was completely resected; on day 20, tumor cells were inoculated on the left side to simulate tumor rechallenge; and from day 20 to day 28, no treatment was given; on day 28, the tumor was sacrificed, and the left-side tumor was excised. b) Individual curves of left-sided rechallenge tumor volume over time for each group. c) Curves of the average left-sided rechallenge tumor volume over time for each group. d) Quantification and e) photographs of the final volume of left-sided rechallenge tumors at the experimental endpoint on day 28 for each group. The data are expressed as the means ± SDs (n = 5); **p* < 0.05, ***p* < 0.01, ****p* < 0.001, *****p* < 0.0001.

**Fig. S26** Ability of the hydrogels to promote RPSTC proliferation and migration. a) The effects of different hydrogels on RPSTC proliferation were detected *via* the CCK-8 method. The effects of different hydrogels on RPSTC migration were evaluated *via* b) cell images (scale = 200 μm) and c) quantitative analysis. The proliferation and migration of HUVECs in CMCGels. The data are presented as the means ± SDs (n = 3); * *p* < 0.05, ** *p* < 0.01, *** *p* < 0.001, **** *p* < 0.0001.

**Fig. S27** Ability of the hydrogels to promote HUVEC proliferation and migration. a) The effects of different hydrogels on promoting HUVEC proliferation were detected *via* the CCK-8 method. The effects of different hydrogels on promoting HUVEC migration were evaluated *via* b) cell images (scale = 200 μm) and c) quantitative analysis. The proliferation and migration of HUVECs in CMCGels. The data are presented as the means ± SDs (n = 3); * *p* < 0.05, ** *p* < 0.01, *** *p* < 0.001, **** *p* < 0.0001.

**Fig. S28** a) Changes in body weight and b) H&E staining images (scale = 100 μm) of major organs (heart, liver, lung, and kidney) in rats after treatment for pancreatic fistula with different treatments. The data are presented as the means ± SDs (n = 4).

**Fig. S2****9** Assessment of inflammation in the fistula stumps of rats after different treatments for pancreatic fistula. a) Adhesion and b) inflammatory area scoring system. c) Adhesion and d) inflammatory area scores with different treatments. The data are presented as the means ± SDs (n = 4); * *p* < 0.05, ** *p* < 0.01, *** *p* < 0.001, **** *p* < 0.0001.

**Fig. S30** Transcriptome sequencing of the CMCGel hydrogel for *in vivo* pancreatic fistula repair. a) GO function enrichment results, b) KEGG function enrichment results, and c) Reactome enrichment results.

**Fig. S31** FN, integrin-β1, p-FAK, PPARγ and TGF-β1 levels (as measured by WB). The data are expressed as the means ± SDs (n = 3). 1 represents the control, 2 represents the CMCGel, and 3 represents the CMCGel + inhibitor. The inhibitors used included E7820 (which inhibits fibronectin (FN) and integrin-β1), PF-573228 (which inhibits focal adhesion kinase (FAK) kinase activity), pirfenidone (which inhibits TGF-β1), and FH535 (which inhibits peroxisome proliferator-activated receptor gamma (PPARγ)). The data are presented as the means ± SDs (n = 3).

**S3 Supporting Tables**

**Table S1** Correlation analysis between the occurrence of POPF in pancreatic cancer patients and their DFS and OS

| Reference number | The primary endpoint was overall survival | Treatment | Number of cases | POPF grading | Incidence of POPF | Follow-up duration | Average / Median age (years) of POPF occurrence / Non-POPF occurrence | Comparison of survival duration between POPF occurrence and non-POPF occurrence | *P* value | References |
| --- | --- | --- | --- | --- | --- | --- | --- | --- | --- | --- |
| 1 | DFS | curative pancreatectomy | 52 | ≥ grade B POPF | 13% (n = 7). | 2000 days | 65 (61–78) VS 68 (49–85) | POPF was an independent risk factor for worse hazard ratio 5.856 | *p* = 0.002 | PMID: 33160278 |
| 2 | DFS | PD | 942 | CR-POPF, Severe POPF | CR-POPF: 9.56% (n = 90), Severe POPF: 4.35% (n = 41). | 16.5 (1.45 - 46.13) months | N/A | 16 VS 21 months | *p* = 0.02 | PMID: 29685757 |
| 3 | DFS | PD was performed in 73 patients (60 %), DP in 47 (38 %), and total pancreatectomy in 2 (2 %). | 122 | POPF | 16% (n = 20) | 60 months | 68 (48–84) VS 70 (43–84) | N/A | *p* < 0.01 | PMID: 27704248 |
| 4 | DFS | PD, DP, or duodenum-preserving pancreatic head resection | 340 | CR-POPF (grade B or C) | CR-POPF: 17.9% (n = 61), POPF grade B: 8.8% (n = 30), POPF grade C: 9.1% (n = 31). | 150 months | POPF B: 62.5 (21–81), POPF C: 70 (46–82), Without POPF: 64 (15–86) | N/A | *p* < 0.001 | PMID: 34086792 |
| 5 | DFS | pancreatectomy | 1397 | grade A, grade B, grade C | Total POPF: 23.4% (n = 327), grade A: 9.9% (n = 139), grade B: 10.6% (n = 148), grade C: 2.9% (n = 40). | 120 months | POPF B: 66 (45–90), POPF C: 66 (51–78), Without POPF: 67 (27–91) | 7.4 VS 12.9 months | *p* = 0.028 | PMID: 27083480 |
| 6 | DFS | pancreatic resection for adenocarcinoma | 767 | grade B, grade C | Total POPF: 10.7% (n = 82), grade B: 8.7% (n = 67), grade C: 2% (n = 15). | 96 months | 65.2 ± 10.8 VS 67.4 ± 10.5 | 8.0 vs 15.73 months | *p* = 0.031 | PMID: 33526357 |
| 1 | OS | curative pancreatectomy | 52 | ≥ grade B POPF | 13% (n = 7). | 2000 Days | 65 (61–78) VS 68 (49–85) | POPF was an independent risk factor for worse hazard ratio 7.097 | *p* = 0.020 | PMID: 33160278 |
| 2 | OS | PD | 942 | CR-POPF, Severe POPF | CR-POPF: 9.56% (n = 90), Severe POPF: 4.35% (n = 41). | 16.5 (1.45 - 46.13) months | N/A | 22 VS 25 months | *p* = 0.005 | PMID: 29685757 |
| 3 | OS | PD was performed in 73 patients (60 %), DP in 47 (38 %), and total pancreatectomy in 2 (2 %). | 122 | POPF | 16% (n = 20) | 60 months | 68 (48–84) VS 70 (43–84) | N/A | *p* < 0.01 | PMID: 27704248 |
| 4 | OS | PD, DP, or duodenum-preserving pancreatic head resection | 340 | CR-POPF (grade B or C) | CR-POPF: 17.9% (n = 61), POPF grade B: 8.8% (n = 30), POPF grade C: 9.1% (n = 31). | 150 months | POPF B: 62.5 (21–81), POPF C: 70 (46–82), Without POPF: 64 (15–86) | N/A | *p* = 0.002 | PMID: 34086792 |
| 5 | OS | pancreatectomy | 1397 | grade A, grade B, grade C | Total POPF: 23.4% (n = 327), grade A: 9.9% (n = 139), grade B: 10.6% (n = 148), grade C: 2.9% (n = 40). | 120 months | POPF B: 66 (45–90), POPF C: 66 (51–78), Without POPF: 67 (27–91) | 9 VS 23.6 months | *p* < 0.001 | PMID: 27083480 |
| 6 | OS | pancreatic resection for adenocarcinoma | 767 | grade B, grade C | Total POPF: 10.7% (n = 82), grade B: 8.7% (n = 67), grade C: 2% (n = 15). | 96 months | 65.2 ± 10.8 VS 67.4 ± 10.5 | 20.22 vs 26.33 months | *p* = 0.027 | PMID: 33526357 |

Note: N/A indicates “Not applicable”, PD: pancreaticoduodenectomy, POPF: postoperative pancreatic fistula, CR-POPF: clinically relevant - POPF, DP: distal pancreatectomy.

**Table S2** Antibodies used in this study

| **Antibody** | **Application** | **Dilution** |
| --- | --- | --- |
| CRT | IF | 1:100 |
| HMGB1 | IF | 1:200 |
| CD8α | IF | 1:100 |
| IFN-β | IF | 1:200 |
| IL-6 | IF | 1:500 |
| STING | WB | 1:1,000 |
| p-STING | WB | 1:1,000 |
| TBK1 | WB | 1:1,000 |
| p-TBK1 | WB | 1:1,000 |
| IRF3 | WB | 1:1,000 |
| p-IRF3 | WB | 1:1,000 |
| β-Actin | WB | 1:5,000 |
| GAPDH | WB | 1:5,000 |
| Histone H3 | WB | 1:2,000 |
| FN (Fibronectin) | WB | 1:1,000 |
| Integrin-β1 | WB | 1:1,000 |
| p-FAK | WB | 1:1,000 |
| PPARγ | WB | 1:1,000 |
| TGF-β1 | WB | 1:1,000 |
| HRP-conjugated Goat Anti-Rabbit (secondary) | WB | 1:3,000 |
| HRP-conjugated Goat Anti-Mouse (secondary) | WB | 1:3,000 |
| Cy3-conjugated Goat Anti-Rabbit (secondary) | IF | 1:400 |

**Table S3** Mechanistic Synergy Evidence for CMCGel-Mediated PDT-CDT Combination: From Molecular ROS Amplification to Systemic Abscopal Effects

| **Parameter** | **PDT Alone** | **CDT Alone** | **Expected Additive** | **Combination** | **Synergy Index** |
| --- | --- | --- | --- | --- | --- |
| ROS Generation (Fig. 3e) | ~15.9× | ~1.6× | ~17.5× | 52.17× | 2.98 |
| CRT Exposure (Fig. 3f, S15) | 6.17× | 0.97× | 7.14× | 10.05× | 1.41 |
| DC Maturation (Fig. 3l, 5a-d) | Moderate | +57% | — | 38.31% | 2.4× vs CDT |
| TIEs (Primary Tumor) (Fig. 5g) | Low | ~17.6% | — | 58.37% | 3.31× vs CDT |
| TIEs (Distant Tumor) (Fig. 5h) | None (untreated) | ~15.4% | — | 49.57% | 3.21× vs CDT |
| Distant Inhibition (Fig. S20d) | None (untreated) | None (untreated) | 0% | 88.36% | Abscopal Effect |

**Table S4** Comprehensive Synergy Assessment Across Multiple Levels of CMCGel-Mediated PDT-CDT Combination Therapy

| **Level of Analysis** | **Method** | **Synergy Evidence Strength** |
| --- | --- | --- |
| *In vitro* cell inhibition | Valeriote | Weak synergy (R = 1.02) |
| *In vivo* tumor inhibition | Indirect inference | Strong synergy (R = 1.93) |
| ROS generation | Fold comparison | Strong synergy (2.98×) |
| ICD induction | CRT/ATP/HMGB-1 | Moderate synergy (1.4–1.6×) |
| Immune activation | TIEs/Tregs | Strong synergy (3.31×) |
| Systemic effect | Distant inhibition | Strongest synergy (emergent, from none) |
